# Photoabsorption of 1–2 nm molecular Ce-oxo nanoclusters *versus* ceria: intervalence charge transfer but no size effects†

**DOI:** 10.1039/d5sc00905g

**Published:** 2025-05-28

**Authors:** Stephen E. Brown, Sebastian D. Pike

**Affiliations:** a Department of Chemistry, University of Warwick CV4 7AL Coventry UK sebastian.pike@warwick.ac.uk

## Abstract

The absorption spectra of a series of Ce-oxo clusters (molecular nanoparticles) with sizes ranging from 3 to 100 Ce atoms per cluster are reported. The O 2p to Ce 4f charge-transfer absorption onset in these systems is unaffected by size and almost identical to that of 5 nm CeO_(2−*x*)_ particles and other Ce based materials such as the metal–organic framework Ce-UiO-66. This clearly demonstrates that in CeO_2_ based systems, with highly localized 4f LUMO orbitals, quantum confinement effects are not influential on electronic structure. Importantly, this allows the use of ultrasmall ceria particles in (visible light) photochemical applications without detrimental band-gap enlarging size-effects. Changes in colour in CeO_2_ materials and these Ce-oxo clusters are instead clearly attributed to the presence of (defective) surface sites. Surface Ce(iii) sites in mixed-valence clusters contribute a Ce(iii)/Ce(iv) intervalence charge transfer transition in the visible region, which affects their colour. Ce_24_ clusters with a range of aliphatic and aromatic carboxylate ligands are studied, to compare the effect of surface ligands on the electronic structure. Ligand influence on the absorption spectra is only observed when highly conjugated (naphthyl) or strongly electron donating substituents (anisole or aniline) are present.

## Abstract 2

The absorption spectra of 1–2 nm molecular Ce-oxo clusters show no size effects relative to larger nanoparticles of CeO_(2−*x*)_, but an additional intervalence charge transfer band is present when Ce(iii) sites are present in the cluster.

## Graphical abstract

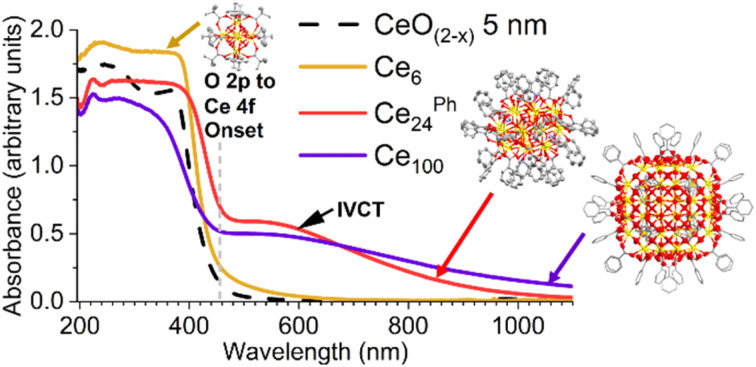

## Introduction

Cerium dioxide is an important earth-abundant n-type semiconducting material widely used in catalytic converters,^1^ self-cleaning materials, water purification,^2^ catalytic antioxidants in medicine,^2,3^ and (photo)catalysis, with recent interest in the use of ultrasmall (<3 nm) CeO_2_ particles.^4,5^ CeO_2_ adopts the fluorite structure in which each Ce is coordinated by eight O atoms in a cubic coordination geometry, with Ce–O distances of 2.37 Å and Ce⋯Ce distances of 3.87 Å. Whilst the majority of Ce atoms exist in the Ce(iv) oxidation state, the material naturally exists in a non-stoichiometric form with formula CeO_(2−*x*)_, with oxygen vacancies and associated Ce(iii) sites that are concentrated on the surface of particles.^5–8^

To effectively use these systems for photocatalytic applications it is essential to have a good understanding of the electronic structure and light absorption properties. The major electronic excitation in CeO_2_ is an oxygen 2p (valence band) to cerium 4f charge transfer (oxygen to metal charge transfer, OMCT) transition.^9^ The contracted empty 4f orbitals of Ce are localized (atomic-like), leading to a very narrow ‘band’ of energy states.^10–12^ Band gap charge transfer transitions are responsible for the photoactivity in wide-band gap metal oxide semiconductors and can lead to effective charge separation and redox reactivity. Following photoexcitation of CeO_(2−*x*)_, the excited electron rapidly (<2 ps) couples with phonons (vibrations) to generate a small polaron state (distorted lattice directly around the generated Ce^3+^ state), and conductivity within CeO_(2−*x*)_ is believed to occur *via* a small polaron hopping mechanism.^10,13,14^

The colour of ceria materials varies from white to pale yellow, depending on the synthetic process, with varying values for the band gap reported (indirect gap 2.5–3.3 eV, direct gap 3.2–3.6 eV), noting that the method of analysis can give different values.^10,15–17^ Yellow colours imply the ability to absorb blue light, *e.g.* lower energy photons can perform electronic excitations. These lower energy processes are suggested to involve defective surface sites,^18^ sometimes termed the ‘Urbach tail’, attributed to surface disorder, and may be dependent on Ce^3+^ content.^10,12^ The presence of Ce(iii) sites, with a 4f^1^ electron configuration, may affect the electronic spectra, as the 4f electrons may be excited using low energy (visible light) *via* intervalence charge transfer processes.^9^ Further excitation from a 4f^1^ (polaron) state to the empty 5d band is expected to require higher energies of 3.2–3.7 eV in the UV range.^10^ In this article the spectra of defective nano-ceria is compared with well-defined molecular Ce-oxo clusters, where any ‘defects’ are clearly resolved in the precise atomic structure, providing a more detailed understanding of these phenomena.

With increasing interest in ultrasmall (high surface-area-to-volume) nanomaterials, it is important to understand how the properties of materials change with size. Nanoscaling commonly effects the electronic properties of a semiconductor material, as the valence and conduction bands becomes less continuous and narrower through the process of quantum confinement.^19^ The result of quantum confinement is typically an enlargement of the band gap and blue-shift of the absorption onset. Quantum confinement effects are expected to occur once the size of a nanoparticle shrinks below the exciton Bohr-radius of the material. For some low band gap materials, such as CdS, this effect is observed over a wide size range (particles <20 nm) resulting in a large range of band gaps for differently sized particles. The exciton Bohr-radius of CeO_(2−*x*)_ is not well defined, however, in other semiconductors with similar band-gaps size effects are only observed at very small sizes (ZnO < 7 nm, TiO_2_ <2 nm).^20,21^ To date, reports of quantum confinement effects in CeO_2−*x*_ are inconsistent, with some reports suggesting a blue shift in small nanoparticles (*e.g.* 2.9 eV for ∼6 nm particles shifting to ∼3.1 eV for 3–4 nm particles, by measuring the indirect allowed transition),^22,23^ whilst other reports indicate a slight red-shift in the band gaps relative to bulk CeO_(2−*x*)_ for ultrasmall particles (*e.g.* 2.5 eV for 6–8 nm particles^17^).^12^ Other studies suggest no quantum confinement effect in fully oxidized CeO_2_ nanoparticles, ranging from ∼7 nm to ∼1.5 nm.^24,25^ The consequence of an unchanging absorption onset with size is significant, as improving the surface-area by nanoscaling CeO_(2−*x*)_ would not result in reduced solar light absorption.

A further study shows that the optical properties of ∼2 nm CeO_(2−*x*)_ nanoparticles quantitatively correlate with the value of *x* and the valence state of the Ce atoms.^26^ The optical density of the particles decreases (bleaches) as they become reduced, resulting in a weaker absorbance (which could erroneously be assigned as a blue shift in onset). These results are confirmed by other reports, which suggest that the optical density decreases as Ce(iii) forms at the surface, essentially decreasing the volume of the CeO_2_ core.^24,27^ It is also noteworthy that nanoscaling of CeO_(2−*x*)_ leads to an increase in the lattice parameter once below 20 nm (0.45% larger at 6 nm), which may be caused by an increased proportion of Ce(iii) + oxygen vacancies or due to increased strain at the surface sites which coordinate with adsorbed species.^12,28^

Considering the variable chemical state of CeO_(2−*x*)_ nanoparticles *via* their (size-dependent) interaction with local environment, it may not be sensible to apply a classical interpretation of quantum confinement which considers a change in properties on shrinking volume of a consistent chemical state.^29^ It is also noteworthy that particle shape and well-defined facets in larger nanoparticles of CeO_2_ (*e.g.* 9–100 nm) may also effect their UV/visible absorption spectra and reported band gap.^30,31^

One challenge in characterising nanoparticle systems is their inherent polydispersity and a lack of understanding at the atomic level. In contrast, precisely defined molecular metal-oxo clusters provide exceptional understanding of atom connectivity in monodisperse systems. Recently, fascinating nanoscale Ce-oxo clusters (or molecular nanoparticles) have been discovered, with up to 100 Ce atoms (2.4 nm Ce–O maximum core diameter).^32–37^ These remarkable structures exhibit the CeO_2_ fluorite lattice in their Ce–O cores (Fig. S1†) which is then supported by surface organic ligands such as anionic carboxylates and neutral N-donor ligands (similar to surfactant stabilized nanoparticles).^38^ Reported structures exhibit differing degrees of Ce(iii) occupancy varying from 0–40% of Ce atoms,^34,39^ identified from bond length analysis (bond valence sum calculations) of their molecular crystal structures. Furthermore, even smaller Ce-oxo clusters, such as Ce(iv)_6_O_4_(OH)_4_(carboxylate)_12_,^40^ or similar,^41,42^ are highly relevant for this discussion, especially due to their common use in important metal–organic framework (MOF) structures (*e.g.* Ce-UiO-66) and in photocatalysis.^40,43–46^ In these molecular systems, almost the entirety of the structure is ‘surface’ and ligand orbitals significantly contribute to the electronic structure.^47^ Therefore, it is important to consider whether the ligand can introduce mid-gap (HOMO) orbitals which may reduce the HOMO–LUMO gap (which is the molecular equivalent of the band gap), this is more likely for ligands with conjugated structures with high lying filled orbitals, and such ligands are commonly studied for their relevance to dye-sensitised materials.^48,49^ Ce-oxo clusters ranging from Ce_6_–Ce_38_ and bulk CeO_2_ have been previously studied by X-ray absorption spectroscopy, to show the transition from a surface dominated molecule to a molecular nanoparticle, containing core Ce atoms in a cubic crystal field more closely resembling CeO_2_.^37^ This indicated the average crystal field splitting (Δ*O*_h_) of the 5d orbitals increases with particle size, but interestingly the lower energy 2p–4f X-ray absorptions were consistent across the size range, indicating the energy of the 4f orbitals remains consistent.

In this study we compare the electronic absorption (UV/visible) spectra of a range of differently sized Ce-oxo clusters varying from 3–100 Ce atoms which have differing supporting ligands, shapes and Ce(iv)/Ce(iii) ratios and contrast these with typical CeO_(2−*x*)_ materials and MOFs, all using consistent methods. The results show a negligible change in the oxygen to metal (Ce 4f) charge-transfer onset across all size ranges, with all materials maintaining an indirect absorption onset of 2.6–2.9 eV. The lack of quantum confinement contrasts to studies of other metal-oxides,^20,50^ and is of relevance to findings which suggest that highly localized charge transfer excitons can be formed in metal oxide materials.^51^

## Results and discussion

### Spectroscopic results

**Fig. 1:**
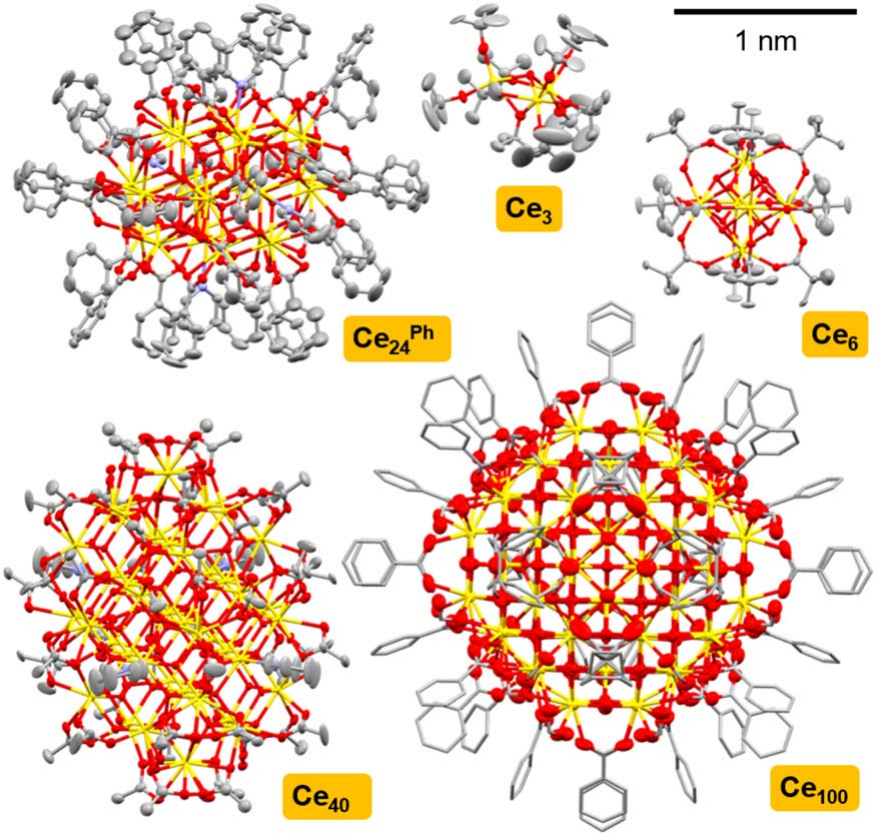
Single crystal X-ray structures of Ce_3_,^53^Ce_6_,^54^Ce_24_^Ph^,^34^Ce_40_,^32^Ce_100_^33^ to scale. Ellipsoids at 50% probability, H-atoms omitted for clarity, C atoms shown as capped sticks in Ce_100_. Ce = yellow, O = red, N = blue, and C = grey.

A series of Ce-oxo clusters were prepared as crystalline materials using literature routes, ranging from small cerium-(oxo)-alkoxide species [Ce(iv)_3_O(O^*t*^Bu)_10_] (Ce_3_),^52^ through high symmetry [Ce(iv)_6_O_4_(OH)_4_(O_2_C^*t*^Bu)_12_] (Ce_6_),^35^ to larger ‘molecular nanoparticles’ [Ce_22_(iv)Ce_2_(iii)_4_O_28_(OH)_8_(O_2_CPh)_30_(py)_4_] (Ce_24_^Ph^),^32,33^ [Ce_38_(iv)Ce(iii)_2_O_56_(OH)_2_(O_2_CMe)_44_(MeCO_2_H)_2_(py)_4_] (Ce_40_)^32^ and [Ce(iv)_92_Ce(iii)_8_O_149_(OH)_18_(O_2_CPh)_60_(H_2_O)_20_](O_2_CPh)_8_(NO_3_)_8_ (Ce_100_)^33^ (Fig. 1 and S3–S11, Table S2†). Structures were confirmed by single-crystal X-ray diffraction and bulk powders contain the expected C, H, N content by elemental analysis (Table S1†) (note that purity of air-sensitive Ce_3_ was instead determined *via* solution NMR spectroscopy, Fig. S3†). The PXRD pattern of Ce_6_, implies a trace impurity of larger clusters may also be present (Fig. S5†). In Ce_3_ the Ce atoms adopt octahedral coordination geometries (face-sharing octahedra, Ce⋯Ce distances 3.527 Å), whilst square antiprismatic (eight-coordinate) geometry is found in Ce_6_ (edge-sharing square antiprisms, Ce⋯Ce distances 3.764–3.769 Å), and the larger clusters all mimic the cubic coordination geometry of the fluorite structure at their core, although surface Ce(iv) sites vary in coordination geometry (for Ce_24_^Ph^, Ce⋯Ce distances range 3.7–3.9 Å).^32,33^ In Ce_24_^Ph^, Ce_40_ and Ce_100_ Ce(iii) atoms are also present, and these have ten-coordinate geometries and are found exclusively on the cluster surface.^32,33^

**Fig. 2:**
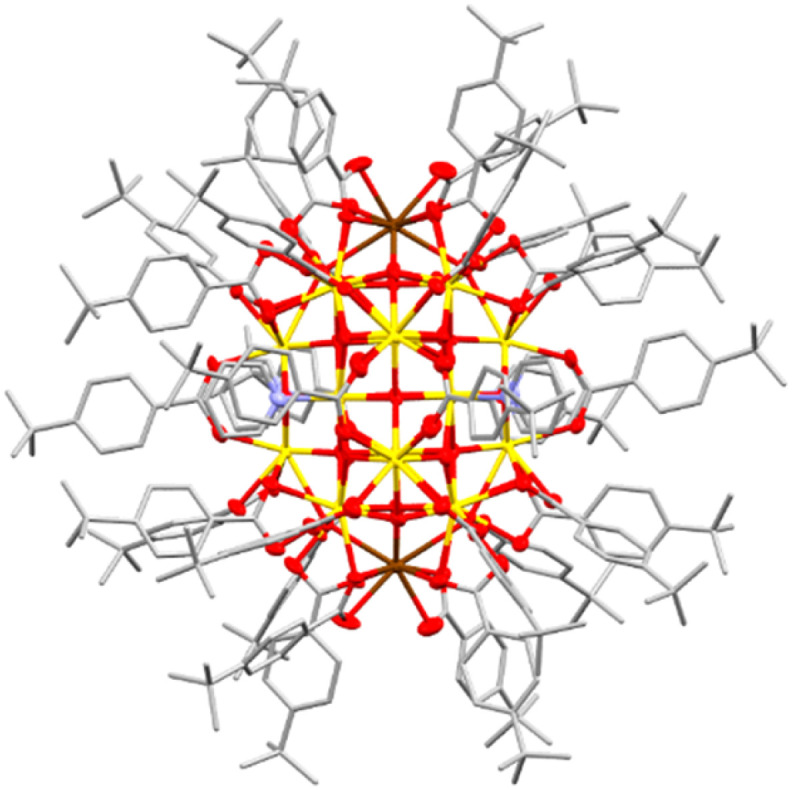
Single crystal X-ray structure of Ce_24_^Ph^*^t^*^Bu^. Ellipsoids at 50% probability, H-atoms omitted for clarity, C atoms shown as capped sticks. Ce(iv) = yellow, Ce(iii) = brown, O = red, N = blue, and C = grey.

**Fig. 3:**
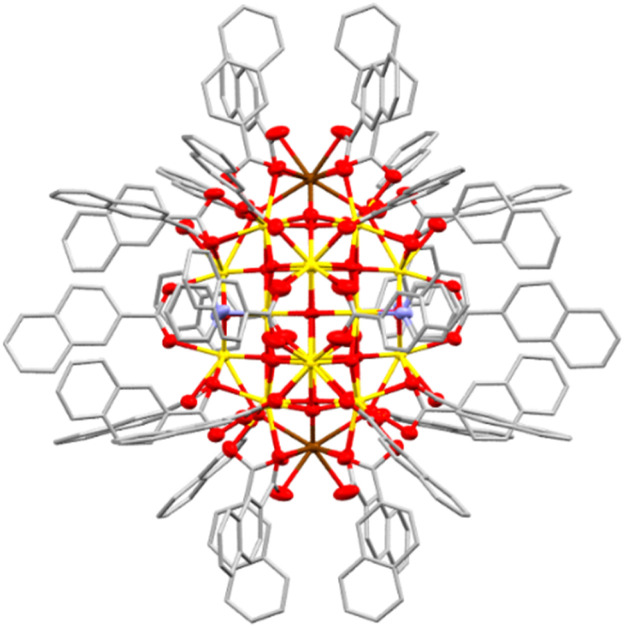
Single crystal X-ray structure of Ce_24_^2Nap^. Ellipsoids at 50% probability, H-atoms and non-coordinated pyridine molecules omitted for clarity, C atoms shown as capped sticks. Ce(iv) = yellow, Ce(iii) = brown, O = red, N = blue, and C = grey.

To expand this series in order to evaluate the effect of surface ligands, a set of novel Ce_24_ clusters were prepared with the same overall formula as Ce_24_^Ph^ but with different carboxylate ligands (O_2_CR: R = *n*-C_3_H_7_, Ce_24_^But^; *n*-C_5_H_11_, Ce_24_^Hex^; *p*-C_6_H_4_Me, Ce_24_^PhMe^; *p*-C_6_H_4_^*t*^Bu, Ce_24_^Ph^*^t^*^Bu^; *p*-C_6_H_4_F, Ce_24_^PhF^; *p*-C_6_H_4_OMe, Ce_24_^PhOMe^; *p*-C_6_H_4_NH_2_, Ce_24_^PhNH2^; 1-naphthyl, Ce_24_^1Nap^, 2-naphthyl, Ce_24_^2Nap^). All structures form with isostructural Ce_24_ cores (Fig. 2, 3 and S12–S17†). Where enough ligand information could be resolved in the single crystal X-ray structure (Ce_24_^Ph^, Ce_24_^PhMe^, Ce_24_^PhF^, Ce_24_^PhOMe^, Ce_24_^1Nap^, Ce_24_^2Nap^) bond valence sum analysis confirmed the expected location of two surface Ce(iii) sites on opposite ends of the cluster core (Fig. 2 and 3, Tables S2–S7†).^55^ The structures with aliphatic carboxylates, Ce_24_^But^ and Ce_24_^Hex^ demonstrate that benzoate ligand pi interactions are not essential for structure assembly. Assessing bulk purity of these paramagnetic compounds (which are typically insoluble in inert solvents) is challenging, especially as crystallinity is lost upon drying the samples (see Fig. S19†), however, elemental analysis of these new compounds is mostly in excellent agreement with the expected formula (Table S1†). Further analysis of bulk purity and near IR spectroscopy is included in the ESI.†

The synthetic process that generates these Ce_24_ clusters consists of dissolving 24 equivalents of carboxylic acid in pyridine/water (10 : 1 ratio), generating a basic solution of pyridinium carboxylate. To this, four equivalents of Ce(iii)(NO_3_)_3_(H_2_O)_6_ is added, most likely forming [Ce(iii)(carboxylate)_3_]_*x*_ structures in solution (ESI Note 1†), it is crucial that these species remain solubilized at this stage.^56^ Next, one equivalent of (NH_4_)_2_Ce(iv)(NO_3_)_6_ is added and the reaction mixture layered with acetone. The reaction solution slowly darkens from yellow to brown and Ce_24_ compounds crystallise. The crystalline yield is typically below 20% total Ce (or <100% Ce(iv) reagent), therefore, we hypothesise that the [Ce(iii)(carboxylate)_3_]_*x*_ solution is important for allowing the clusters to form, but that the predominantly Ce(iv) clusters are mostly built from Ce(iv) atoms from (NH_4_)_2_Ce(iv)(NO_3_)_6_.

**Fig. 4:**
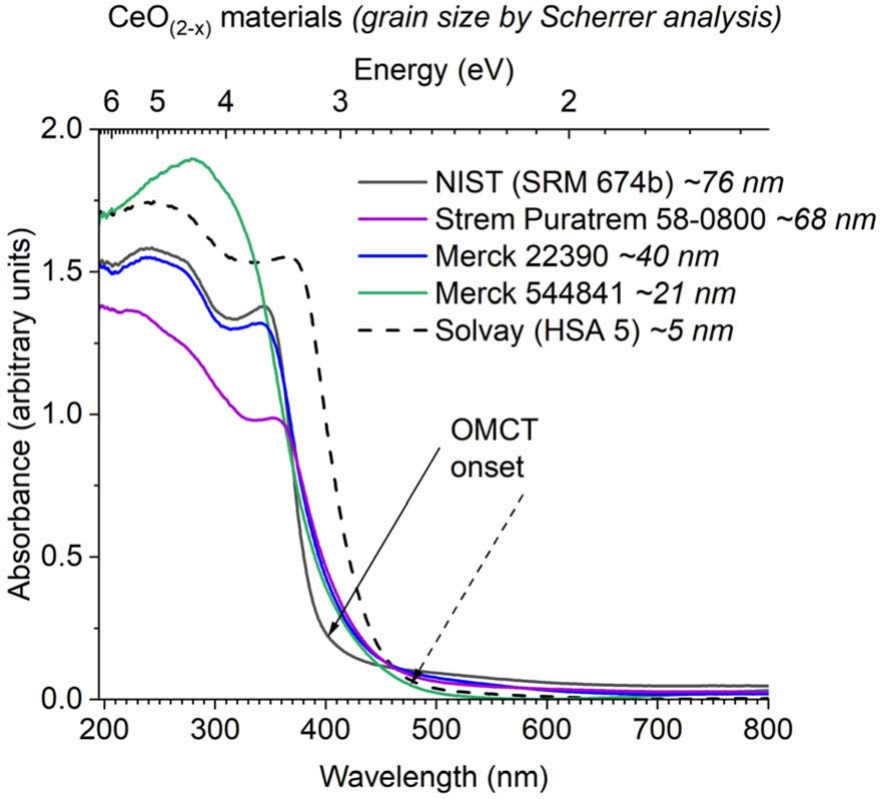
Diffuse reflectance UV/vis spectra of commercial ceria (nano)materials. Arrows show calculated onset value from Tauc's analysis for largest and smallest particles.

The diffuse reflectance UV/visible spectra of a range of commercially available ceria materials were collected (Fig. 4), these materials all display expected powder-X-ray diffraction patterns for ceria (Fig. S22, Table S8†), and no other metals (except trace Al) were detected above the detection limit of X-ray fluorescence spectroscopy (Table S9†). In this study all absorption onsets are calculated using Tauc's method of plotting (*F*(*R*_∞_)*hν*)^1/*n*^*vs. hν*, with *n* = 3 for an indirect forbidden (2p to 4f) process (Fig. S23†), it is worth noting the potential error (∼±0.1 eV) in these calculations, and always worth visually comparing collected spectra rather than only the ‘onset’ value.^57^ The data demonstrates the differing absorption onsets in ceria materials (Fig. 4); the NIST standard CeO_2_ (∼75 nm grain size, low defect density) is white with an onset of 3.1 eV. In contrast, a high surface area (HSA) sample (Solvay HSA 5, ∼5 nm particles) is yellow and displays a red-shifted onset at 2.6 eV. Other ‘standard’ ceria samples are pale yellow and show similar onsets to the HSA sample, calculated to be 2.6–2.8 eV, but with a spectral profile which more resembles the NIST sample at higher energies. Therefore, we suggest the highly crystalline NIST sample and the high surface area HSA sample act as the outliers, with ‘standard’ ceria behaving like a defective version of the NIST sample.

**Fig. 5:**
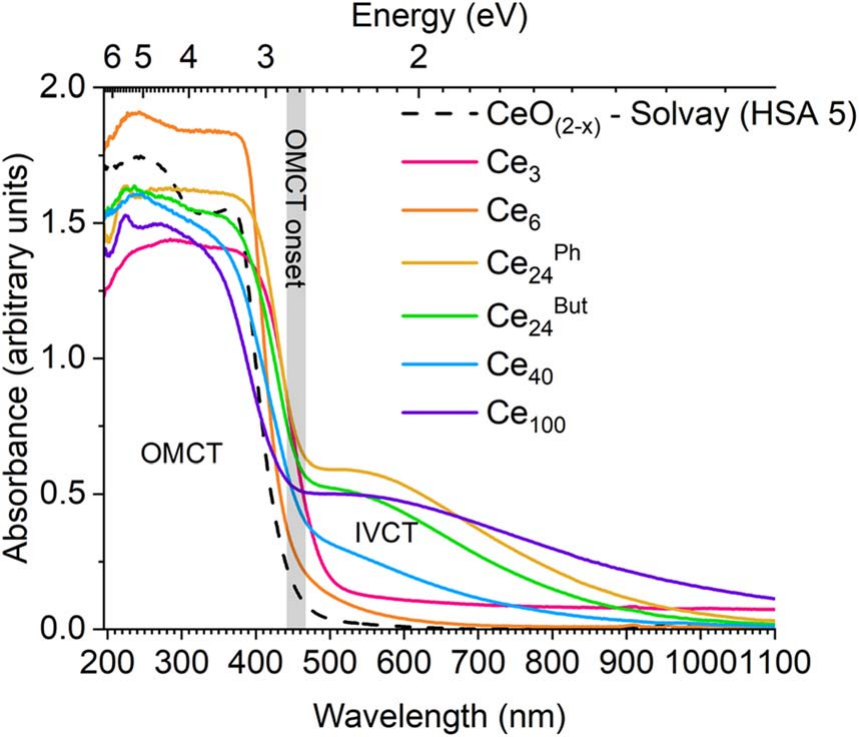
Diffuse reflectance UV/vis spectra of Ce-oxo clusters in comparison to high surface area CeO_(2−*x*)_. Note the steep increase, attributed to the OMCT absorption, begins at a similar value for all solid compounds except Ce_3_ (onset region determined by Tauc's analysis highlighted in grey).

The absorption spectra of Ce-oxo clusters was collected and compared to 5 nm ceria (Fig. 5). The spectra clearly show that the major OMCT excitation (with ‘band-gap like’ O 2p to Ce 4f character) begins at very similar onset values for all of these compounds (range; 2.7–2.8 eV, Table S10†), and the spectra are remarkably similar to HSA ceria. A small difference is noted for yellow/orange Ce_3_ (onset 2.4 eV) which has only oxo and alkoxide ligands and a different coordination geometry at Ce.

Ce_3_ and pale yellow Ce_6_ only have absorptions in the OMCT region, noting that these are only expected to contain Ce(iv) sites, therefore, there is no possibility of intervalence charge transfer (IVCT), 4f → 5d or 4f → ligand excitations in these cases. In contrast, the molecular nanoparticles Ce_24_^Ph^, Ce_24_^But^, Ce_40_ and Ce_100_ are brown, with additional absorptions (centered at ∼525–550 nm) at lower energies than the OMCT band. This absorption in the visible region correlates with the Ce(iii) content in these mixed valence compounds; Ce_24_^Ph^, Ce_24_^But^, and Ce_100_ are reported to have 8% Ce(iii), whilst Ce_40_ has 5% Ce(iii) and consequently has a weaker absorption in the visible region. The nature of these visible excitations is attributed to a metal to metal charge transfer process, *i.e.* a Ce(iii)/Ce(iv) IVCT. Visible-light IVCT excitations from Ce(iii) sites have previously been proposed as the cause for darkening of CeO_2_@ethylene glycol nanoparticles under UV irradiation, with colour bleaching when subsequently heated under air.^58^ To confirm that this excitation is not 4f to ligand (carboxylate/pyridine π*) based (*i.e.* metal to ligand charge transfer (MLCT)), or 4f to 5d, the coordination polymer [Ce(iii)(O_2_CCH_2_^*t*^Bu)_3_Py]_∞_ was studied. This compound contains only 9-coordinate Ce(iii) sites that are coordinated to carboxylate and pyridine ligands. [Ce(O_2_CCH_2_^*t*^Bu)_3_Py]_∞_ exhibits a charge transfer absorption onset at 2.7 eV (N.B. commercial Ce(acetate)_3_ hydrate absorbs from 3.0 eV), but no further absorptions are observed in the visible region, discounting the presence of 4f MLCT or 4f–5d excitations (Fig. S24–S27†) in Ce(iii) only systems.

**Fig. 6:**
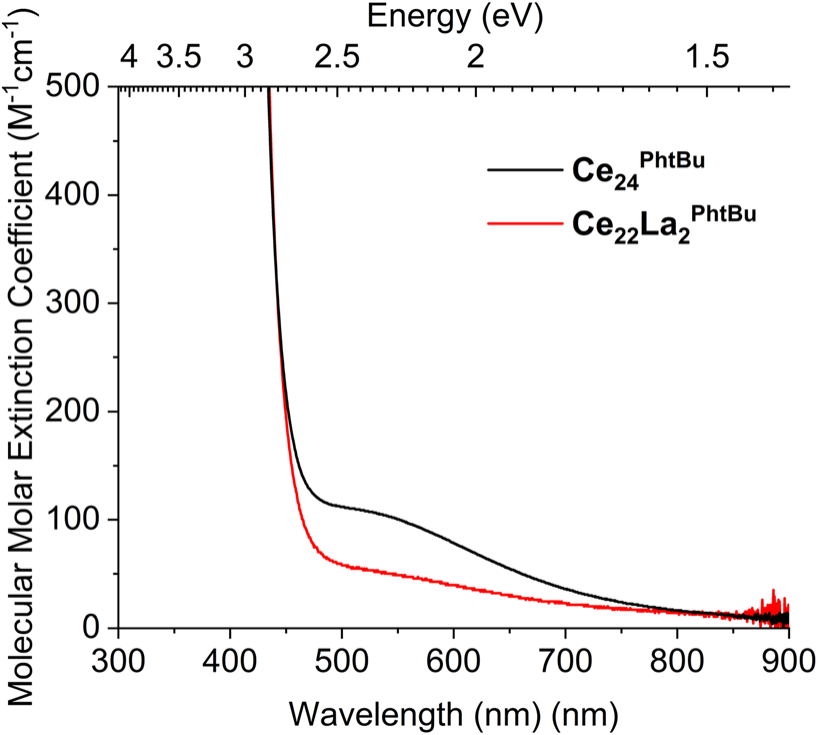
Solution UV/vis spectra of Ce_24_^Ph^*^t^*^Bu^ and Ce_22_La_2_^Ph^*^t^*^Bu^.

To further characterize these compounds and the IVCT excitation, the solution UV/visible spectrum of the readily soluble compounds Ce_3_ and Ce_24_^Ph^*^t^*^Bu^ were collected in toluene, and THF for the latter (Ce_3_ 0.9 mM, Ce_24_^Ph^*^t^*^Bu^ 1.3 mM, Fig S28 and S29†). The OMCT onset in the solution spectrum of Ce_24_^Ph^*^t^*^Bu^ shows good agreement to the solid-state data (solid, 2.69 eV; toluene, 2.71 eV; THF, 2.69 eV, Fig. S30†), which is also very similar to that of Ce_3_ in solution (toluene, 2.69 eV). The onset of Ce_3_ is blue-shifted relative to when in the solid-state, perhaps due to its very small size and the significant structural changes possible on solvation (Fig. S31†). The Ce_24_^Ph^*^t^*^Bu^ data is similar to previous reports of the solution UV spectra of Ce_24_^Ph^ and Ce_100_ in DMF solvent, but with greater confidence that THF or toluene do not partake in any redox reactivity or structural rearrangement.^33^ The IVCT band appears as a weak feature (*e*^max^: toluene, 100 M^−1^ cm^−1^; THF, 140 M^−1^ cm^−1^) in the solution spectrum of Ce_24_^Ph^*^t^*^Bu^, consistent with the pale brown colour of the solution (Fig. 6 and S28†), and requires a mM concentration to be observed – noting that weaker peaks are more prominent in non-linear solid-state UV/vis absorption data in comparison to the linear absorption scale for solution data. Gaussian peaks were fit to the OMCT and IVCT regions of the spectra suggesting the IVCT signal is somewhat dependent on solvent (Fig. S32,† THF, max. 513 nm, FWHM 288 nm; toluene, max. 555 nm, FWHM 220 nm). This broad, weak, solvent-polarity dependent feature in the visible region is consistent with a Robin–Day class II system, in which there is some localisation of valence character in the system but a low activation barrier for interconverting between valence states. This agrees with the bond valence sum analysis of these (low-symmetry) Ce_24_ compounds, in which surface sites corresponding to Ce(iii) are identified (Tables S2–S7†). In higher symmetry Ce-oxo clusters, the location of well-defined Ce(iii) sites is less obvious, and these may be better described as Robin–Day class III systems, with lower energy IVCT transitions.^36,59^ IVCT in lanthanide species is rare, with few clearly defined examples of M(iii)/M(iv) IVCT,^36,59,60^ however, the UV/visible spectrum of intermediate-valence [Ce_2_(IO_3_)_6_(OH_0.44_)] shows a similar broad feature at 625 nm,^59^ and the mixed valence cluster Ce(iii)_4_Ce(iv)_6_O_8_(acac)_14_(CH_3_O)_6_(CH_3_OH)_2_, with 40% Ce(iii) displays a strong broad feature in its solid-state UV/vis spectrum. Furthermore, recent interest in M(iii)/M(ii) systems (*e.g.* f_13_/f_14_ configurations in Yb systems) also show broad spectral features (*e*^max^ 258 M^−1^ cm^−1^ at 600 nm, and *e*^max^ 150 M^−1^ cm^−1^ at 725 nm).^61,62^ The four Ce(iii)⋯Ce(iv) distances in Ce_24_^Ph^*^t^*^Bu^ range 3.73–3.80 Å. A (partial) metal–metal bond is identified in a mixed-valence molecule with Dy(ii)–Dy(iii) distance of 3.71 Å,^63^ noting that this bond forms from the large 5d orbitals, in contrast, molecules with Yb(ii)⋯Yb(iii) distances of 2.95 and 3.58 Å, with only f-orbital occupancy, do not exhibit metal–metal bonds,^61,62^ and, therefore, we do not expect any 4f-based metal–metal bonding in these M(iii)/(iv) oxo clusters.

We hypothesised that replacing the two Ce(iii) sites in Ce_24_^Ph^*^t^*^Bu^ with La(iii) (without an f electron) should remove the IVCT band and result in a yellow compound. The synthetic process was repeated but using La(NO_3_)_3_(H_2_O)_6_ instead of Ce(iii)(NO_3_)_3_(H_2_O)_6_, whilst retaining (NH_4_)_2_Ce(iv)(NO_3_)_6_ as the Ce(iv) source. The resultant solution remained yellow, without darkening to brown, and produced yellow crystals which gave the expected single crystal structure for Ce_24_^Ph^*^t^*^Bu^. Inductively coupled plasma optical emission spectroscopic analysis of the product revealed a good match for the expected La and Ce content in a cluster with formula [Ce_22_(iv)La_2_(iii)_4_O_28_(OH)_8_(O_2_CC_6_H_4_Bu)_30_(py)_4_], Ce_22_La_2_^Ph^*^t^*^Bu^, (La wt%, found 3.15%, calc. 2.90%; Ce wt%, found 30.3%, calc. 32.19%). Solution UV-visible spectroscopy confirmed that the IVCT band for Ce_22_La_2_^Ph^*^t^*^Bu^ was significantly reduced in this mixed metal compound, consistent with the requirement for Ce(iii) for the IVCT absorption (Fig. 6). The same approach was also used to prepare ‘Ce_22_La_2_^Ph^’ as a yellow material (ESI Note 3†).

Due to the high surface content in these compounds, ligand effects are surely important. Mashima and co-workers previously calculated the HOMO of acetate capped Ce_6_ clusters [Ce_6_O_4_(OH)_4_(O_2_CR)_12_] to be composed of a combination of oxo, hydroxo and carboxylate O orbitals, whilst in the benzoate analogue the phenyl pi orbitals become the HOMO states, but with the O-based orbitals only 0.1 eV lower in energy. In both cases the Ce 4f orbitals contribute to the LUMO states, and the major contribution to the absorption spectrum is O 2p to Ce 4f charge transfer (OMCT). In our study, switching between benzoate and aliphatic carboxylates has no effect on the absorption spectrum, confirming that the pi system of benzoate does not play a major role. Furthermore, the similarity of Ce_3_, which only has oxo and alkoxide ligands, is consistent with an O centered HOMO state without requirement for carboxylate.

**Fig. 7:**
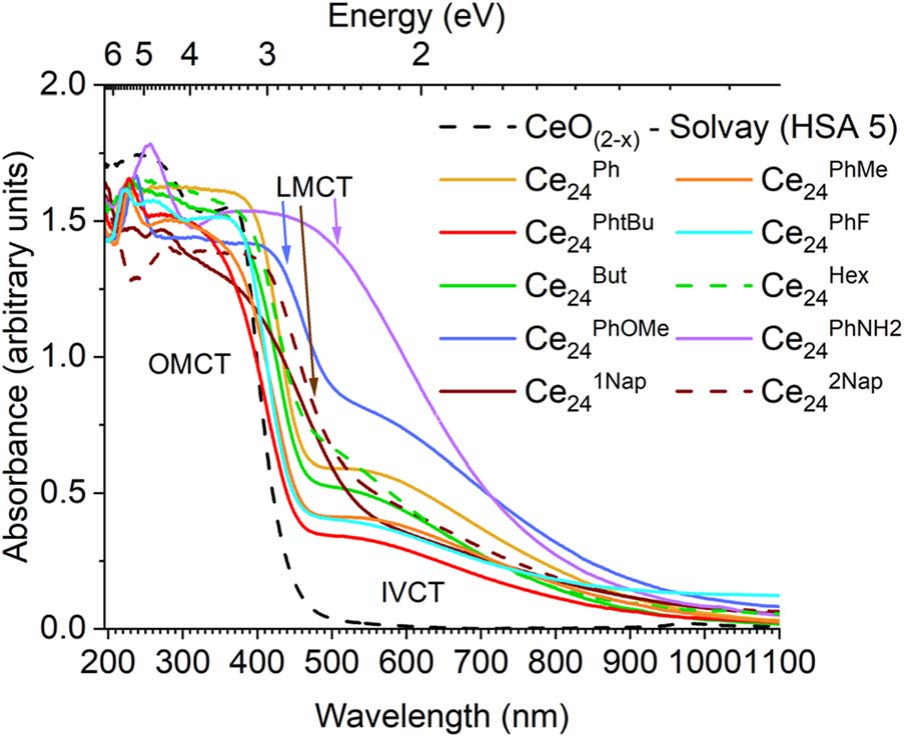
Diffuse reflectance UV/vis spectra of Ce_24_-oxo clusters in comparison to high surface area CeO_(2−*x*)_.

The impact of substituents in the *para* position of benzoate was probed, along with extending the pi system to naphthoate ligands (Fig. 7 and S33†). The OMCT absorption onset remains similar for electron withdrawing (Ce_24_^PhF^, 2.7 eV) or weakly donating (Ce_24_^PhMe^, 2.7 eV; Ce_24_^Ph^*^t^*^Bu^, 2.7 eV) *para* substituents. However, the absorption becomes slightly shifted for moderately donating (Ce_24_^PhOMe^, 2.5 eV) substituents; and is significantly different for the strongly donating *para*-aminobenzoate ligand (Ce_24_^PhNH^2, 1.2 eV, dark red colour), and is now better termed ligand to metal charge transfer (LMCT). Using larger conjugated pi systems in the naphthoate clusters, also causes a lower energy LMCT onset (Ce_24_^1Nap^, 2.3 eV, Ce_24_^2Nap^, 2.4 eV), in these scenarios the ligand pi system is likely to contribute significantly to the HOMO and/or LUMO. These are well known ligand effects, used in dye sensitised metal oxide materials and MOFs, and allow for tailoring of the visible light absorption properties, but with impact on the location and redox potentials of photoexcited charges.^40,47,48,64,65^

**Fig. 8:**
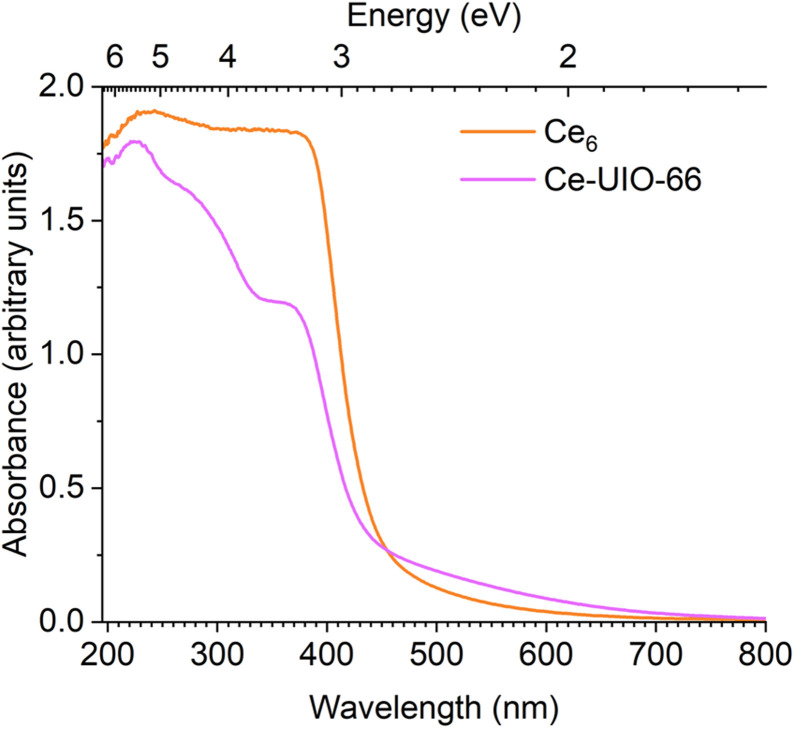
Diffuse reflectance UV/vis spectra of Ce6 cluster in comparison to a sample of Ce-UiO-66.

Finally, to consider the effect of double ended carboxylate linkers, the spectrum of Ce_6_ was compared with the MOF Ce-UiO-66 in which the pivalate ligands are replaced with terephthalate linkers (Fig. 8).^43,48^ The MOF exhibits a similar absorption onset, if slightly blue shifted, to the individualised cluster (Ce-UiO-66, 2.9 eV, Ce_6_, 2.7 eV), however, it is noteworthy that literature absorption onset values for Ce-UiO-66 vary (down to 2.5 eV) and show dependence on the amount of defects in the MOF.^48,66^ This batch of MOF, prepared in DMF but without added modulators, has been previously reported to have 22% missing linkers,^67^ the spectra shows a tail into the visible region, consistent with previously reported defective Ce-UiO-66.^43,66^ This implies the presence of Ce(iii) defects in the sample that accompany missing linkers and that contribute visible light IVCT contributions to the spectrum.

### Discussion

The OMCT absorptions of the Ce_6_–Ce_100_ clusters are almost identical to ∼5 nm (HSA ceria) nanoparticles. Whilst the presence of an ‘Urbach tail’ (from defective surface sites) is used to explain the visible absorption in ceria, this cannot be used for well-defined crystalline molecular systems. Instead, the surface sites and oxidation states are well described, allowing for a greater level of understanding of the excitations involved.

It is remarkable that the OMCT excitation in this range of clusters and nanomaterials retains at a consistent absorption onset. It might be expected that as the particles become smaller, due to a diminishing number of atomic orbitals contributing to the valence and conduction bands, the bands would become more discreet (molecule like) and, therefore, narrower, resulting in a larger band gap (OMCT onset) as is observed in other metal oxides. In CeO_2−*x*_ the lowest unoccupied energy states are built from combinations of contracted 4f orbitals. These 4f orbitals are not strongly affected by crystal field, which may explain similarities between species with different coordination geometries.^37^ Since adjacent 4f orbitals overlap poorly, the range of energy states from in and out of phase combinations of these 4f orbitals may be very small, essentially leading to a series of near degenerate energy, rather localized, states. Upon reducing the number of contributing 4f states, these energies remain close to unchanged, so that the lowest unoccupied energy states remain similarly positioned for all sizes.^37^ It is noteworthy that in studies of other wide band gap d^0^/d^10^ metal oxides such as ZnO, it has been found that the majority of quantum confinement effects originate from an upward shift in the conduction band minimum, and that the top of the valence band energy stays relatively fixed.^68^

**Fig. 9:**
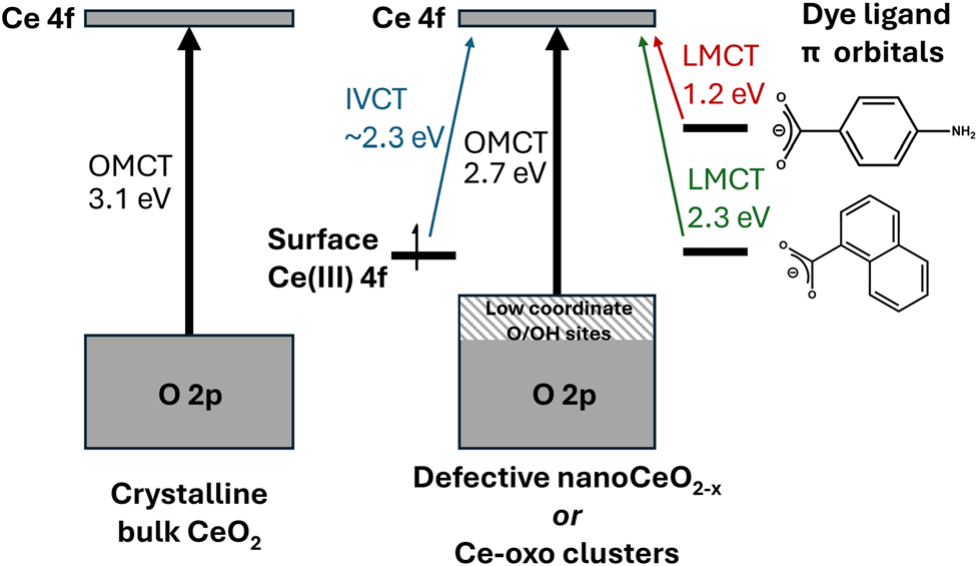
Diagram proposing the major contributions to the absorption spectra of CeO_2_ and (dye-sensitised) mixed valence Ce-oxo clusters. Note that dye ligands may also contribute to LUMO position.

From the comparison of (∼5 nm) nanosized and larger ceria particles (Fig. 3), it is evident that defects and surface sites play an important role in the absorption spectra, but cause smaller (more defective) particles to have red-shifted absorption onsets compared to large bulk particles.^6,18^ Low coordinate O/OH sites may contribute to the highest energy states in the valence band (Fig. 9).^69^ Theoretical studies of wide band gap group 2 metal oxides find that the frontier orbital energies are located on undercoordinated edge or corner atoms, and in materials exhibiting these effects, band shrinking (quantum confinement effects) is masked by the presence of these size-independent surface energy levels.^70^ Combining a steady Ce 4f energy position, with a surface and/or defect dominated valence band maximum goes some way to explain why there is no observed quantum confinement effect in ceria as both frontier orbital positions remain stable with changing size (Fig. 9).

In the structurally precise Ce-oxo clusters studied here the presence of surface Ce(iii) does not change the OMCT absorption onset but does introduce a IVCT transition. Whilst this IVCT signal is very broad, its maxima at ∼550 nm (2.3 eV) is larger than may be expected for moving an electron from a Ce(iii) site in CeO_(2−*x*)_ to the unoccupied 4f band (∼1 eV),^6^ which perhaps reflects the specific coordination environments of Ce atoms in these low-symmetry molecules. It is noteworthy that the term ‘oxygen vacancies’ is not a good descriptor for discussing the electronic structure of these mixed valence clusters, as Ce(iii) sites are adequately charge balanced by the surface ligand environment. It will be fascinating to see whether targeting this visible-light IVCT can lead to productive photoredox chemistry. Characterisation of visible excitations in these systems is important as it helps to describe the nature of electronic transitions that contribute to the ‘Urbach tail’ observed in defective ceria nanomaterials and visible absorption in oxygen deficient ceria.^8,10,12,18^ An enhanced understanding of the contributing factors for visible light excitation of ceria materials is essential for their deployment in sunlight driven photocatalytic processes.

## Conclusions

The UV/visible spectra of a range of differently sized molecular Ce-oxo clusters from a Ce_3_ alkoxide cluster to molecular nanoparticles up to 2.4 nm in core diameter are recorded, and all show consistent OMCT absorption onsets compared to 5 nm CeO_(2−*x*)_ particles. This clearly shows that the quantum confinement effect is not observed in ceria particles, and that the frontier unoccupied orbital is best described as a localised 4f orbital on Ce(iv). The presence of Ce(iii) sites introduces IVCT transitions across the visible region, and causes the compounds to appear brown-coloured. Introducing dye ligands such as *para*-aminobenzoate or naphthoate, alters the electronic structure such that the frontier orbitals incorporate the organic pi system, and, therefore, the resulting LMCT absorption onset occurs at lower energies (Fig. 9).

A consistent electronic structure for nanoceria down to even the smallest sizes of Ce-oxo clusters is very important for photocatalysis as it shows that abundant blue light (*e.g.* from sunlight) can be used to excite these structures regardless of their size, noting that solar spectral irradiance drops sharply once into the UV region. Therefore, ultrasmall ceria, which can be designed for solution processibility, should be useful for high scale processes that use natural light such as photocatalytic water splitting or in self-cleaning materials.^40,46^ Furthermore, added visible light absorption is possible upon introduction of Ce(iii) sites as moderate energy excitation below the band gap allows promotion from Ce(iii) to the Ce(iv) core (IVCT). This opens up possibilities for designing materials with ultrasmall ceria particles or Ce-oxo clusters with optimal surface area and atom economy but without reducing their performance in absorbing sunlight.

## Data availability

ESI (experimental methods, supporting characterisation and spectra, X-ray crystallography details and analysis).† All raw data is deposited in the University of Warwick's database, WRAP at https://wrap.warwick.ac.uk/191521/. CCDC 2418676–2418679, 2418699–2418701 and 2418814 contain the supplementary crystallographic data for this paper.

## Author contributions

S. D. Pike conceived and supervised the project. S. E. Brown conducted all experimental work. Both authors wrote the manuscript.

## Conflicts of interest

There are no conflicts to declare.

## Supplementary Material

SC-OLF-D5SC00905G-s001

SC-OLF-D5SC00905G-s002
