# Supplementary material for: Photoabsorption of 1–2 nm molecular Ce-oxo nanoclusters versus ceria: intervalence charge transfer but no size effects
Source: Chem Sci. 2025 May 28;16(25):11659–68. doi: 10.1039/d5sc00905g (PMC12127974; doi:10.1039/d5sc00905g)
Supplement: SC-016-D5SC00905G-s001 [file SC-016-D5SC00905G-s001.pdf]

Supplementary Information (SI) for Chemical Science.
This journal is © The Royal Society of Chemistry 2025

Photoabsorption of 1-2 nm Molecular Ce-oxo Nanoclusters Versus Ceria: Intervalence Charge Transfer but No Size Effects.

Stephen E. Brown,[a] Sebastian D. Pike*[a]

[a]Department of Chemistry, University of Warwick, CV4 7AL, Coventry, UK

Contents

Experimental Details	2
Supporting Note 1. Discussion of the synthesis of Ce24 clusters	4
Synthesis and characterisation of compounds	4
Discussion of Bulk Purity	11
Supporting Figures and Tables	14
Supporting Note 2. NIR signals	47
Supporting note 3. Preparation of Ce22La2Ph cluster	48
Crystallography details	49
Crystallography table	51
References	54

Experimental Details

Cerium ammonium nitrate and cerium nitrate hexahydrate were purchased from Fluka and Sigma Aldrich respectively. X-ray fluorescence confirms that no other metals (other than trace Al) are present in these starting materials. Ceria materials were used directly from suppliers, Ce-UIO-66 was prepared by Dr. Baiwen Zhao using a reported literature procedure without added modulators, details of characterisation of this batch of sample can be found in the published article by Walton et al.[46]

Solvents were used as received from suppliers, except when air/moisture-free conditions were required in the preparation of $\mathbf{Ce_3}$.

Air/moisture-free conditions

'Anhydrous' toluene and THF was purchased from Sigma Aldrich. All air-free solvents were degassed by three freeze-pump-thaw cycles or by bubbling with N_2 for 30 minutes and stored over 4 Å molecular sieves. d_8 toluene, d_6 benzene, d_8 THF were dried by stirring over CaH_2 and vacuum distilled using grease-free trap-to-trap apparatus, before storing over 4 Å molecular sieves under nitrogen. Molecular sieves were activated before use by heating under vacuum (>150°C) until the head space partial pressure was less than 10^{-1} mbar. Moisture analysis of prepared solvents was conducted by Karl Fischer spectrometric analysis: heptane, 6.3 ppm; toluene, 5.6 ppm; pyridine, 8.8 ppm; THF, 10.7 ppm.

UV-visible spectroscopy

Solution UV-visible spectroscopy was recorded using a Implen NanoPhotometer C40 or a Shimadzu 2600i, using a bespoke Young's tap cuvette for air free analysis.

Solid-state (SS) UV-visible-NIR spectroscopy was carried out on a Shimadzu 2600i fitted with an integrating sphere attachment. Samples were prepared in a quartz window cell with the sample placed inside a 1 mm deep PTFE washer and the cell sealed with PTFE tape on the threads. For $\mathbf{Ce_3}$ a 0.5 mm deep quartz cell sealed with a quartz plate by vacuum grease inside the glovebox was used to ensure air-tight conditions; the resulting spectrum was similar to $\mathbf{Ce_3}$ recorded in the standard cell. The Kubelka-Munk function was used to derive absorption over scattering and subtracted from a background of $BaSO_4$ (collected in the same cell as the experiment). The sample of $BaSO_4$ is shared between measurements to minimise variations in the measurement of the background.

The absorption onset is described by: $A = B(h\nu - Eg)^n / h\nu$ Where A is absorption, B is the absorption constant for the transition, Eg is the energy gap in eV and $h\nu$ is the photon energy. Absorption was modelled with a cubic dependence on increasing energy and the exponent n is given a value of 3 (for a forbidden direct transition, noting the O 2p to Ce 4f transition involved). A plot of $(Ah\nu)^{1/3}$ vs $h\nu$ gives a straight line. The onset region of solid-state data was fitted with a straight line with a R^2 value of ≥ 0.99. The onset of solution data was fitted with a line from abs = 2 towards the onset, retaining a R^2 value of ≥ 0.98. The point of absorption onset (in eV) was derived at the x-value of the intercept of this straight line with a straight line describing the baseline (to avoid any error from extrapolating through an imperfect baseline).[47] Note that is some cases the baseline is in fact the curve of the visible IVCT transition, in this case the baseline was modelled as a tangent of the IVCT curve at the onset of the new charge transfer peak.

IR spectroscopy

Solid-state Fourier transform infrared spectra were recorded using an Agilent Cary 630 FTIR with an ATR Sampling Accessory.

NMR spectroscopy

Solution NMR spectra were recorded on Bruker 400 MHz spectrometers at 298 K. Chemical shifts are quoted in ppm and coupling constants in Hz and referenced to the proton impurity in the deuterated solvent.

Elemental Analysis

C, H, N elemental analysis was conducted by Orla McCullough at London Metropolitan University upon samples which had been dried overnight at a pressure of 10^{-2} mbar.

Inductive Coupled Plasma (ICP) Optical Emission Spectroscopy (OES) Analysis

Ce and La ICP OES analysis was conducted by Exeter Analytical upon samples which had been dried overnight at a pressure of 10^{-2} mbar.

X-ray Fluorescence

X-ray fluorescence spectroscopy was collected on a Rigaku Primus IV Wavelength Dispersive X-Ray Fluorescence Spectrometer. Elemental detection limits below parts per thousand mass%. Results present mass% not accounting for mass of O.

Powder X-ray Diffraction

PXRD patterns were collected on a Panalytical Empyrean with a Cu anode source in reflection geometry. Scherrer analysis including peak fitting was done in HighScore Plus.[48] To study in the presence of reaction solvents crystalline samples were kept in the crystallisation solution and loaded into capillaries, and high resolution PXRD patterns collected at the i11 beamline at Diamond light source (λ = 0.823756 Å)

Single crystal X-ray diffraction

Crystals of cerium oxo clusters were kept in the mother solution then mounted directly onto pins under fomblin oil and swiftly under the cryostream to prevent loss of solvent and crystallinity. Crystals of cerium-oxo clusters lose volatile solvent on standing in air and are degraded resulting in loss of crystallinity over time.

Single crystal X-ray diffraction datasets were collected on a Rigaku Supernova or Synergy S or beamline i19 EH1 at Diamond Light Source. Data integration was done using CrysAlis Pro or DIALS automated pipeline.[49] Structural solution was carried out with SUPERFLIP[50] and refined using CRYSTALS[51] implementation of SHELXS[52]. Solved structures were analysed using SQUEEZE[53] from PLATON[54] software to probe void volumes and identify disordered solvent. Where void volumes contained electron density an appropriate number of pyridines was suggested to compensate the volume and electron charge. This is consistent with the crystallisation solvent and with well-resolved co-crystallised solvent molecules.

Supporting note 1. Discussion of the synthesis of Ce_{24} clusters

General preparation of Ce_{24}-oxo clusters

Ce_{24}-oxo clusters were prepared via an adapted literature route for the synthesis of a benzoate Ce_{24}-oxo cluster.[55] 24 equivalents of carboxylic acid (6.0 mmol) was dissolved in a solution of pyridine and water (10 mL, pyridine 10:1 water). To which 4 equivalents of cerium nitrate hexahydrate $Ce(NO_3)_3(H_2O)_6$ (434 mg, 1.0 mmol) were added and the solution stirred for 30 mins. 1 equivalent of cerium ammonium nitrate $(NH_4)_2Ce(NO_3)_6$ (137 mg, 0.25 mmol) was added and the solution stirred for a further 1 hr. For some carboxylic acids a small amount of precipitate formed at this stage and was removed by filtration. The resulting solution was layered with acetone (25 mL) until crystals of cerium-oxo cluster formed, which were separated by filtration and washed with acetone (2 × 5 mL), then dried under vacuum.

During this study the reported synthetic procedure to **Ce_{24}** clusters was examined. carboxylic acid (24 equivalents) is first dissolved in pyridine and water (10:1 ratio), generating a basic solution of pyridinium benzoate. $Ce(III)(NO_3)_3(H_2O)_6$ (4 equivalents) is added to this. Studies with various carboxylates in this work indicate that the solution then contains $[Ce(III)(carboxylate)_3]_x$ structures along with pyridinium nitrate. If using tert-butylacetic acid, the helical coordination-polymer $[Ce(O_2CCH_2{}^tBu)_3(py)]_\infty$ crystallises directly from the solution (Fig S24). In the case of 4-flourobenzoic acid a single ^{19}F NMR environment is observed in the solution. For preparation of Ce-oxo clusters it is essential that these Ce(III) compounds remain dissolved at this stage. To the $[Ce(III)carboxylate_3]_x$ solution, cerium(IV) ammonium nitrate (1 equivalent) is added, which leads to slow darkening of the solution from yellow to brown and crystallization of Ce_{24} clusters. The crystalline yield is typically less than 20% with respect to total cerium, or less than 100% with respect to Ce(IV) reagents. We hypothesized that the Ce(III) containing solution was important for allowing the clusters to form, but that the clusters were mostly built from the Ce(IV) atoms from cerium(IV) ammonium nitrate.

Synthesis and characterisation of compounds

$[Ce_3O(O^tBu)_{10}]$ (**Ce_3**).

Ce_3 is an air sensitive compound and requires the use of air free manipulations. Schlenk and glovebox techniques under a nitrogen atmosphere were used for manipulation of this compound.

Prepared by an adapted literature procedure.[56] Cerium ammonium nitrate (CAN, 5.48 g, 10.0 mmol) was dried under vacuum at 120 °C and then dissolved in THF (30 mL). NaO^tBu (5.71 g, 60.0 mmol) was dissolved in 40 mL of THF and was added slowly to the CAN solution. Pale yellow precipitate forms immediately after addition and the solution changes colour from red to yellow, and ammonia gas is released. The precipitate was removed by filtration and washed with pentane (2 × 10 mL). The solvent was removed completely by vacuum. The yellow powder was redissolved in heptane and stirred for 96 hours before storing at –20 °C. Yellow needles of **Ce_3** form. The solvent was decanted and the remaining solid dried under vacuum (1.85 g yield, 1.58 mmol, 48% wrt Ce in CAN).

^1H NMR (400 MHz, d^8 toluene): δ 1.92 (s, 36H μ_3/μ_2-$OC(C\mathbf{H}_3)_3$), 1.44 (s, 27H μ_1-$OC(C\mathbf{H}_3)_3$), 1.43 (s, 27H μ_1-$OC(C\mathbf{H}_3)_3$).

$[Ce_6O_4(OH)_4(O_2C^tBu)_{12}]$ (**Ce_6**).

Ammended Synthesis based on a literature preparation.[57] Note that this amended process generates the product rapidly with the intention of generating a Ce(IV) only product.

2.17 g (5 mmol) of $Ce(NO_3)_3.6H_2O$ was dissolved in 150 mL MeCN and 2 mL water. To this, 1.2 g (12 mmol) of pivalic acid was added, followed by 3.6 mL of pyridine. The solution was stirred for 1 hour. 0.55 g (1 mmol) of $(NH_4)_2Ce(NO_3)_6$ was dissolved in 25 mL MeCN and this added to the reaction flask. The solution rapidly produces a fine pale coloured precipitate and is stirred for a further 4 hours before being left overnight. The solution was filtered to leave a pale-yellow powder that was dried under vacuum. Yield = 250 mg, 69% relative to Ce(IV) starting material, 11% relative to total Ce content.

CHN analysis: Calculated for $C_{60}H_{112}Ce_6O_{32}$: C, 32.96 % H 5.16 % N 0%, found C 31.95%, H 4.57% N 0.21 %.

$[Ce_{40}O_{56}(OH)_2(OOCMe)_{44}(py)_4(L)_2]$ (**Ce_{40}**) (L = pyridine and/or HOOCMe),[55b] was synthesised from a modification of the prep for **Ce_{24}^{Ph}**.[55c]

Glacial acetic acid (5.76 g, 5.49 mL, 96.0 mmol) was dissolved in a solution of pyridine and water (160 mL, pyridine 10:1 water). To which cerium nitrate hexahydrate, $Ce(NO_3)_3(H_2O)_6$, (6.94 g, 16.0 mmol) was added and stirred for 30 mins to give a clear colourless solution. Cerium ammonium nitrate, $(NH_4)_2Ce(NO_3)_6$, (2.19 g, 4.00 mmol) was added and the resulting pale yellow solution stirred for 1 hr. The resulting clear solution was layered with acetone (300 mL). Crystals appeared after 4 weeks, and after 6 weeks the precipitate was filtered from an orange solution, washed with acetone and dried under vacuum to give a yellow powder of **Ce_{40}** (398 mg, 41.7 µmol, yield 5.0% w.r.t Ce).

CHN analysis: calculated for $C_{108}H_{154}Ce_{40}N_4O_{146}$: C, 13.73 H 1.64 % N 0.59 %. found C 13.25%, H 1.61%, N 1.15 % Note loss of 'L' co-crystallised solvents.

$[Ce_{100}O_{149}(OH)_{18}(O_2CPh)_{60}(HO_2CPh)_{12}(H_2O)_{20}](O_2CPh)_8(NO_3)_8$ (**Ce_{100}**) was prepared using a literature route,[55a] at a scale of 2.74 g (5.0 mmol) CAN and yielding 560 mg (20.4 µmol) of **Ce_{100}** (8.0% yield wrt to Ce).

CHN analysis: calculated for $C_{572}H_{494}Ce_{100}N_8O_{375}$ (including 4 co-crystallised acetone molecules)[55a]: C, 24.99 H 1.81 % N 0.41%. found C 25.98%, H 1.71% N 0.51 %.

$[Ce_{24}O_{28}(OH)_8(O_2CPh)_{30}(py)_4](py)_{14}$ (**Ce_{24}^{Ph}**)

Benzoic acid, PhCOOH, (2.93 g, 24.0 mmol) was dissolved in a solution of pyridine and water (40 mL, pyridine 10:1 water). To which cerium nitrate hexahydrate, $Ce(NO_3)_3(H_2O)_6$, (1.74 g, 4.0 mmol) was added and stirred for 30 mins to give a clear colourless solution. Cerium ammonium nitrate $(NH_4)_2Ce(NO_3)_6$ (548 mg, 1.0 mmol) was added and the resulting straw coloured solution stirred for 1 hr. The solution was layered with acetone (100 mL). Crystals appeared after 2 weeks, after 4 weeks the precipitate was filtered from an orange solution and dried under vacuum to give a brown powder of **Ce_{24}^{Ph}** (110 mg, 13.9 µmol, yield 6.7% w.r.t Ce).

Pale yellow plate crystals suitable for SCXRD were kept in the mother liquor.

Previous reports using this synthesis method yielded the similar molecule $[Ce_{24}O_{27}(OH)_9(OOC(CH_2)_4CH_3)_{30}(py)_4](py)_{10}$ which differs by an additional proton and

corresponding Ce^{3+} site as well as an increased number of solvent pyridine molecules in the unit cell.[55a, 55c] Bond valence sum analysis of the crystals prepared here, was assigned to $[Ce_{24}O_{28}(OH)_8(O_2CPh)_{30}(py)_4](py)_{14}$ with two Ce(III) per cluster.

CHN analysis: calculated for $C_{230}H_{178}Ce_{24}N_4O_{96}$ (without uncoordinated pyridine solvents): C, 34.98 %, H 2.27 % N 0.71%. found C 36.30 %, H 2.18 % N 1.19 %. By including three co-crystallised pyridine molecules the fit is improved; calculated for $C_{245}H_{193}Ce_{24}N_7O_{96}$: C 36.18 %, H 2.39 % N 1.21 %.

ICP-OES: Calculated for $C_{245}H_{193}Ce_{24}N_7O_{96}$: Ce 41.34%, found Ce 40.78%.

$\mathbf{Ce_{22}La_2^{Ph}}$ was prepared using the same route as for $\mathbf{Ce_{24}^{Ph}}$ (2.5 x scale) but by replacing $Ce(NO_3)_3(H_2O)_6$ for $La(NO_3)_3(H_2O)_6$. 75 mg of yellow powder isolated (10 µmol, yield 148% w.r.t. Ce(IV))

CHN analysis: calculated for $C_{245}H_{193}Ce_{22}La_2N_7O_{96}$: C 36.19 %, H 2.39 % N 1.21 found C 35.4 %, H 2.15 % N 0.95 %.

ICP-OES: Calculated for $C_{245}H_{193}Ce_{22}La_2N_7O_{96}$: Ce 37.91 %, La 3.42 %, found Ce 36.00%, La 4.97%

$[Ce_{24}O_{28}(OH)_8(O_2CC_6H_4Me)_{30}(py)_4](py)_{21}$ ($\mathbf{Ce_{24}^{PhMe}}$)

p-Toluic acid, $4\text{-}(CH_3)C_6H_4COOH$, (3.27 g, 24.0 mmol) was dissolved in a solution of pyridine and water (40 mL, pyridine 10:1 water). To which cerium nitrate hexahydrate, $Ce(NO_3)_3(H_2O)_6$, (1.74 g, 4.0 mmol) was added and stirred for 30 mins to give a clear colourless solution. Cerium ammonium nitrate $(NH_4)_2Ce(NO_3)_6$ (548 mg, 1.0 mmol) was added and the resulting pale yellow solution stirred for 18 hr. A small amount of fine cream-coloured ppt formed and was removed by filtration. The resulting clear yellow solution was layered with acetone (100 mL). Crystals appeared after 4 weeks, and after 12 weeks the precipitate was filtered from an orange solution and dried under vacuum to give a brown powder of $\mathbf{Ce_{24}^{PhMe}}$ (109 mg, 13.1 µmol, yield 6.3% w.r.t Ce).

Pale yellow faceted block crystals suitable for SCXRD were kept in the mother liquor. The structure of $[Ce_{24}O_{28}(OH)_8(OOCPh\text{-}4\text{-}Me)_{30}(py)_4]_2(py)_{42}$ with two centrosymmetric halves of $Ce_{24}O_{28}(OH)_8(OOCPh\text{-}4\text{-}Me)_{30}(py)_4$ and 21 solvent pyridines in the asymmetric unit were resolved.

CHN analysis: calculated for $C_{260}H_{238}Ce_{24}N_4O_{96}$: C, 37.55 %, H 2.88 % N 0.67%. found C 37.46 %, H 2.61 % N 0.97 %. Note loss of co-crystallised pyridine molecules during drying under vacuum.

$[Ce_{24}O_{28}(OH)_8(O_2CC_6H_4{}^tBu)_{30}(py)_4](py)_{10}$ ($\mathbf{Ce_{24}^{PhtBu}}$)

4-tert-Butylbenzoic acid, $4\text{-}((CH_3)_3C)C_6H_4COOH$, (21.39 g, 120 mmol) was dissolved in a solution of pyridine and water (200 mL, pyridine 10:1 water). To which cerium nitrate hexahydrate, $Ce(NO_3)_3(H_2O)_6$, (8.68 g, 20.0 mmol) was added and stirred for 30 mins to give a clear colourless solution. Cerium ammonium nitrate, $(NH_4)_2Ce(NO_3)_6$, (2.74 g, 5.00 mmol) was added and the resulting pale yellow solution stirred for 1 hr. A small amount of fine ppt formed and was removed by filtration, and the resulting clear solution was layered with acetone (100 mL). Crystals appeared after 8 weeks, after 16 weeks the precipitate was filtered from an orange solution and

dried under vacuum to give a brown powder of **Ce_{24}^{PhtBu}** [$Ce_{24}O_{28}(OH)_8(OOCPh^tBu)_{30}(py)_4$] (758 mg, 79.2 µmol, yield 7.6% w.r.t Ce).

Yellow trapezoid block crystals suitable for SCXRD were kept in the mother liquor. The structure of [$Ce_{24}O_{28}(OH)_8(OOCPh^tBu)_{30}(py)_4$]$(py)_{10}$ was resolved.

CHN analysis: calculated for $C_{350}H_{418}Ce_{24}N_4O_{96}$: C, 43.88 H 4.40 % N 0.59 %. found C 43.23 %, H 4.42% N 0.46%. Note loss of co-crystallised pyridine molecules during drying under vacuum.

^{1}H NMR (400 MHz, d^8 toluene) δ 12-5 ppm, pyridine and aromatic signals; 2-0 ppm ^{t}Bu signals, some broadening due to paramagnetism, small paramagnetically shifted signal also observed at -37.7 ppm.

List of NMR peaks. 11.95, 10.28, 8.62, 8.41, 8.12, 7.71, 6.62, 6.51, 6.30, 5.89, 5.74, 5.57, 5.42, 1.93, 1.77, 1.65, 1.61, 1.57, 1.57, 1.14, 0.95, 0.82, 0.82, 0.67, 0.39, 0.36, 0.28, 0.27, -37.73

$Ce_{22}La_2^{PhtBu}$ was prepared using the same route (0.5x scale) as for **Ce_{24}^{PhtBu}** but by replacing $Ce(NO_3)_3(H_2O)_6$ for $La(NO_3)_3(H_2O)_6$. 150 mg of yellow plate crystals isolated (16 µmol, yield 14% w.r.t. Ce(IV))

ICP-OES: Calculated for $C_{245}H_{193}Ce_{22}La_2N_7O_{96}$: Ce 32.19 %, La 2.90 %, found Ce 30.30%, La 3.15%

[$Ce_{24}O_{28}(OH)_8(O_2CC_6H_4F)_{30}(py)_4$]$(py)_6$ (**Ce_{24}^{PhF}**)

4-Fluorobenzoic acid, 4-FC_6H_4COOH, (3.36 g, 24 mmol) was dissolved in a solution of pyridine and water (40 mL, pyridine 10:1 water). To which cerium nitrate hexahydrate, $Ce(NO_3)_3(H_2O)_6$, (1.74 g, 4.0 mmol) was added and stirred for 30 mins to give a clear colourless solution. Cerium ammonium nitrate, $(NH_4)_2Ce(NO_3)_6$, (548 mg, 1.00 mmol) was added and the resulting pale yellow solution stirred for 30 minutes. A small amount of fine ppt formed and was removed by filtration, the resulting clear solution was layered with acetone (100 mL). Crystals appeared after 8 weeks, and after 12 weeks the precipitate was filtered from a yellow solution and dried under vacuum to give a brown powder of **Ce_{24}^{PhF}** (61 mg, 7.5 µmol, yield 3.6% w.r.t Ce).

Yellow block crystals suitable for SCXRD were kept in the mother liquor. The structure of [$Ce_{24}O_{28}(OH)_8(OOCC_6H_4(4\text{-}F))_{30}(py)_4$]$_2(py)_{12}$ was resolved, with two half molecules of [$Ce_{24}O_{28}(OH)_8(OOCC_6H_4(4\text{-}F))_{30}(py)_4$] and 6 solvent pyridines in the asymmetric unit.

CHN analysis: calculated for $C_{230}H_{148}Ce_{24}F_{30}N_4O_{96}$: C, 32.75%, H 1.77 % N 0.66%. found C 33.67 %, H 1.63 % N 0.82 %. Calculated for 1 additional co-crystallised pyridine $C_{235}H_{153}Ce_{24}F_{30}N_5O_{96}$: C 33.15 %, H 1.81 % N 0.82 %. Note loss of co-crystallised pyridine molecules during drying under vacuum.

Ce_{24}^{But}, estimated formula: [$Ce_{24}O_{28}(OH)_8(O_2C^nPr)_{30}(py)_4$]$(py)_x$

Butyric acid, ^{n}PrCOOH, (2.12 g, 2.19 mL, 24.0 mmol) was dissolved in a solution of pyridine and water (40 mL, pyridine 10:1 water). To which cerium nitrate hexahydrate, $Ce(NO_3)_3(H_2O)_6$, (1.74 g, 4.0 mmol) was added and stirred for 30 mins to give a clear colourless solution. Cerium ammonium nitrate, $(NH_4)_2Ce(NO_3)_6$, (548 mg, 1.0 mmol) was added and the resulting yellow solution stirred for 1 hr. The solution was layered with acetone (100 mL). Crystals appeared after

3 weeks, and after 8 weeks the precipitate was filtered to give a brown powder of **Ce_{24}^{But}** (137 mg, 19.1 µmol, yield 9.2% w.r.t Ce).

Yellow needle crystals suitable for SCXRD were kept in the mother liquor, these gave an orthorhombic unit cell and a structural solution suitable to confirm a Ce_{24}-oxo core although the organic ligands are not fully resolved. (P m 2_1 n; **a** 27.1952(8) **b** 20.7368(6) **c** 19.9816(6) Å, **α** 90 **β** 90 **γ** 90) Assuming an analogous formula to other Ce_{24}-oxo clusters suggests a formula of $[Ce_{24}O_{28}(OH)_8(OOC^nPr)_{30}(py)_4](py)_x$.

CHN analysis: calculated for $C_{140}H_{238}Ce_{24}N_4O_{96}$: C, 24.45 H 3.49 % N 0.81%. found C 26.84 %, H 3.10% N 0.49%.

Ce_{24}^{Hex}, estimated formula: $[Ce_{24}O_{28}(OH)_8(O_2CC_5H_{11})_{30}(py)_4](py)_x$

Hexanoic acid, $CH_3(CH_2)_4COOH$, (2.79 g, 3.00 mL, 24.0 mmol) was dissolved in a solution of pyridine and water (40 mL, pyridine 10:1 water). To which cerium nitrate hexahydrate, $Ce(NO_3)_3(H_2O)_6$, (1.74 g mg, 4.0 mmol) was added and stirred for 30 mins to give a clear colourless solution. Cerium ammonium nitrate, $(NH_4)_2Ce(NO_3)_6$, (548 mg, 1.0 mmol) was added and the resulting pale yellow solution stirred for 1 hr. The solution was layered with acetone (100 mL). Crystals appeared after 3 days, and after 3 weeks the precipitate was filtered from an orange solution to give a brown powder of **Ce_{24}^{Hex}** (230 mg, 28.7 µmol, yield 13.7% w.r.t Ce).

Pale brown fibrous plate crystals suitable for SCXRD were kept in the mother liquor. A small block was cleaved for synchrotron SCXRD and gave a triclinic unit cell and a structural solution suitable to confirm a Ce_{24}-oxo core although the organic ligands are not resolved. (P -1; **a** 20.47780(4) **b** 20.87490(3) **c** 20.91000(3), **α** 68.359(4) **β** 85.765(5) **γ** 61.627(4)). Assuming an analogous formula to other Ce_{24}-oxo clusters gives $[Ce_{24}O_{28}(OH)_8(OOC(CH_2)_4CH_3)_{30}(py)_4](py)_x$

CHN analysis: calculated for $C_{200}H_{358}Ce_{24}N_4O_{96}$: C, 31.13 H 4.68 % N 0.73 %. found C 29.94 %, H 4.54% N 0.26%.

Ce_{24}^{PhNH2}, estimated formula: $[Ce_{24}O_{28}(OH)_8(O_2CC_6H_4NH_2)_{30}(py)_4](py)_x$

4-aminobenzoic acid, $H_2NC_6H_4COOH$, (1.65 g, 12.0 mmol) was dissolved in a solution of pyridine and water (20 mL, pyridine 10:1 water). To which cerium nitrate hexahydrate, $Ce(NO_3)_3(H_2O)_6$, (868 mg, 2.0 mmol) was added and stirred for 30 mins to give a clear colourless solution. Cerium ammonium nitrate, $(NH_4)_2Ce(NO_3)_6$, (274 mg, 0.5 mmol) was added and the resulting red solution stirred for 1 hr. The solution was layered with acetone (50 mL). Crystals appeared after 1 week, the precipate was filtered to give a red powder of **Ce_{24}^{PhNH2}** (102 mg, 12.2 µmol, yield 11.7% w.r.t Ce).

Red block crystals suitable for SCXRD were kept in the mother liquor. These gave monoclinic unit cell and a structural solution suitable to confirm a Ce_{24}-oxo core for both molecules in the asymmetric unit. Additionally, some carboxylate ligands could be resolved. (P 2/m; **a** 23.8850(13) **b** 41.3343(17) **c** 24.0718(15) Å, **α** 90 **β** 116.644(8) **γ** 90). Assuming an analogous formula to other Ce_{24}-oxo clusters gives formula $[Ce_{24}O_{28}(OH)_8OOCC_6H_4(4\text{-}NH_2))(py)_4]_2(py)_x$

CHN analysis: calculated for $C_{230}H_{208}Ce_{24}N_{34}O_{96}$: C, 33.10 H 2.51 % N 5.71 %. found C 33.06 %, H 2.68% N 7.47%. The higher than anticipated N content suggests incorporation of co-crystallised ammonium nitrate species, potentially a combination of ligated and co-crystallised ammoniumbenzoate nitrate / ammoniumbenzoic acid nitrates.

$[Ce_{24}O_{28}(OH)_8(OOCC_6H_4(4\text{-}OMe))_{30}(py)_4](py)_{11}$ (**Ce_{24}**^{PhOMe})

4-Methoxybenzoic acid, $MeOC_6H_4COOH$, (1.83 g, 12.0 mmol) was dissolved in a solution of pyridine and water (20 mL, pyridine 10:1 water). To which cerium nitrate hexahydrate, $Ce(NO_3)_3(H_2O)_6$, (868 mg, 2.0 mmol) was added and stirred for 30 mins to give a clear colourless solution. Cerium ammonium nitrate, $(NH_4)_2Ce(NO_3)_6$, (274 mg, 0.5 mmol) was added and the resulting yellow solution stirred for 1 hr. The solution was layered with acetone (50 mL). Crystals appeared after 4 weeks and after 6 weeks the precipitate was filtered, washed with acetone (10 mL) then toluene (10 mL) and dried under vacuum to give a brown powder of **Ce_{24}**^{PhOMe} (83 mg, 9.4 μmol, yield 9.4% w.r.t Ce).

Yellow block crystals suitable for SCXRD were kept in the mother liquor, these gave a monoclinic unit cell and a structural solution for $[Ce_{24}O_{28}(OH)_8(OOCC_6H_4(4\text{-}OMe))_{30}(py)_4](py)_{11}$. (P 2_1/c; **a** 36.1757(6) **b** 25.1668(4) **c** 42.5226(8) Å, **α** 90 **β** 110.700(2) **γ** 90).

CHN analysis: calculated for $C_{260}H_{238}Ce_{24}N_4O_{126}$: C, 35.50%, H 2.73 % N 0.64%. found C 35.15 %, H 2.60% N 0.59%. Note loss of co-crystallised pyridine molecules during drying under vacuum.

$[Ce_{24}O_{28}(OH)_8(1\text{-napthoate})_{30}(py)_4](py)_x$ (**Ce_{24}**^{Ph1Nap})

1-Napthoic acid (2.07 g, 12.0 mmol) was dissolved in a solution of pyridine and water (20 mL, pyridine 10:1 water). To which cerium nitrate hexahydrate, $Ce(NO_3)_3(H_2O)_6$, (868 mg, 2.0 mmol) was added and stirred for 30 mins to give a clear colourless solution. Cerium ammonium nitrate, $(NH_4)_2Ce(NO_3)_6$, (274 mg, 0.5 mmol) was added and the resulting yellow solution stirred for 1 hr. The solution was layered with acetone (50 mL). Crystals appeared after 12 weeks and the precipitate was filtered and dried under vacuum to give a yellow/brown powder of **Ce_{24}**^{Ph1Nap} (180 mg, 19.1 μmol, yield 18.3% w.r.t Ce).

Yellow crystals suitable for SCXRD were kept in the mother liquor, these gave a triclinic unit cell and a structural solution suitable to confirm a Ce_{24}-oxo core in the asymmetric unit. Additionally, almost all the carboxylate ligands could be resolved confirming the number of 1-napthoate and coordinated pyridines. Not all solvent molecules could be resolved and some of the naphthyl rings could not be resolved fully. (P -1; **a** 25.3276(8) **b** 29.1536(5) **c** 31.2965(5), **α** 81.1151(15) **β** 82.178(2) **γ** 82.311(2)). Assuming an analogous formula to other Ce_{24}-oxo clusters gives formula $[Ce_{24}O_{28}(OH)_8(OOCC_{10}H_7)_{30}(py)_4](py)_x$

CHN analysis: calculated for $C_{350}H_{238}Ce_{24}N_4O_{96}$: C, 44.73 %, H 2.55 % N 0.60%. found C 46.79 %, H 2.45 % N 1.48 %. Calculated for 6 additional solvent pyridines $C_{380}H_{268}Ce_{24}N_{10}O_{96}$: C 46.23 %, H 2.74 % N 1.42 %.

$[Ce_{24}O_{28}(OH)_8(2\text{-napthoate})_{30}(py)_4](py)_{10}$ (**Ce_{24}**^{Ph2Nap})

2-Napthoic acid (2.07 g, 12.0 mmol) was dissolved in a solution of pyridine and water (20 mL, pyridine 10:1 water). To which cerium nitrate hexahydrate, $Ce(NO_3)_3(H_2O)_6$, (868 mg, 2.0 mmol) was added and stirred for 30 mins to give a clear colourless solution. Cerium ammonium nitrate, $(NH_4)_2Ce(NO_3)_6$, (274 mg, 0.5 mmol) was added and the resulting yellow solution stirred for 1 hr. The solution was layered with acetone (50 mL). Crystals appeared after 1 week and after 6 weeks the precipitate was filtered and dried under vacuum to give a yellow/brown powder of **Ce_{24}**^{Ph2Nap} (53 mg, 5.6 μmol, yield 5.4% w.r.t Ce).

Yellow crystals suitable for SCXRD were kept in the mother liquor, these gave an orthorhombic unit cell and a full structural solution (P bcn; **a** 26.0526(5) **b** 42.5286(7) **c** 35.9018(6)**α** 90 **β** 90 **γ** 90).

CHN analysis: calculated for $C_{350}H_{238}Ce_{24}N_4O_{96}$: C, 44.73 %, H 2.55 % N 0.60%. found C 42.43 %, H 2.40% N 0.93%. Note that a minor impurity phase is likely based on PXRD and CHN analysis data.

$[Ce(O_2CCH_2{}^tBu)_3(py)]_\infty$

3,3-dimethylbutyric acid, $^tBuCH_2COOH$, (2.79 g, 24.0 mmol) was dissolved in a solution of pyridine and water (40 mL, pyridine 10:1 water). To which cerium nitrate hexahydrate, $Ce(NO_3)_3(H_2O)_6$, (1.74 g, 4.0 mmol) was added and stirred for 30 mins to give a transparent colourless solution. Cerium ammonium nitrate, $(NH_4)_2Ce(NO_3)_6$, (548 mg, 1.0 mmol) was added and the resulting yellow solution stirred for 1 hr, becoming turbid. The fine precipitate was removed by filtration. The resulting yellow solution was layered with acetone (100 mL), very long and large needle like crystals appeared after 6 weeks, after 12 weeks the crystalline precipitate was filtered from a yellow solution and dried under vacuum to give colourless crystalline needles of $[Ce(O_2CCH_2{}^tBu)_3(py)]_\infty$ (1.1 g, 2.08 mmol, yield 42% w.r.t Ce). Alternatively, the same procedure without CAN also yields the same structure, without the need to filter off the initial precipitate. Colourless needle crystals have a hexagonal unit cell with an asymmetric unit formula of $[Ce(O_2CCH_2{}^tBu)_3(py)]_4$, (P 6_5 (or P 6_1); **a** 14.11380(10) **b** 14.11380(10) **c** 93.9953(6), **α** 90 **β** 90 **γ** 120). Crystals were predominantly racemically twinned, but a single enantiomorph was collected by cleaving a domain from one needle along its length.

CHN analysis: calculated for $C_{23}H_{38}CeNO_6$: C, 48.92 % H 6.78 % N 2.48 %.

Calculated for $[Ce(O_2CCH_2{}^tBu)_3(py)_{0.4}(H_2O)_{0.6}]_\infty$ $C_{20}H_{36.2}CeN_{0.4}O_{6.6}$: C, 45.49 % H 6.91 % N 1.06 %.

found C 45.66 %, H 6.70% N 1.57 %. It is possible that in the bulk sample, some coordinated solvent sites are occupied by water, and/or solvent is lost under drying.[58]

Discussion of bulk purity

It is difficult to characterise the bulk purity of many of the larger Ce-oxo cluster compounds due to their paramagnetism, insolubility, and complex structures. Unfortunately, the crystals which form in the reaction process lose a significant quantity of co-crystallised solvent molecules on drying leading to loss of crystallinity in the powders, restricting the value of bulk PXRD analysis.

Elemental analysis provides an important examination of bulk composition and is in good agreement with expected formulae in most cases (Table S1). It is noteworthy that the expected C, H, N values are significantly different across the range of compounds. In **Ce_6** the slightly reduced C content (found in multiple batches) is attributed to a minor phase containing larger Ce-oxo pivalate clusters (with lower C:Ce ratio, see Fig S3). In aliphatic carboxylate Ce_{24} and **Ce_{24}^{PhNH2}** clusters the single crystal structure does not allow resolution of co-solvated solvent molecules. For **Ce_{24}^{But}** and **Ce_{24}^{Hex}** we find a slightly greater C content than the expected structure, which may be attributed to retention of co-solvated solvents in the hydrophilic ligand sphere. For **Ce_{24}^{PhNH2}** a significantly higher N content is found, which perhaps indicates the aminobenzoates are protonated with associated NO_3 anions. The C, H, N analysis and also PXRD pattern of **Ce_{24}^{2Nap}** indicate some impurities are present in this sample, it is noteworthy that the UV-is spectra of **Ce_{24}^{1Nap}** and **Ce_{24}^{2Nap}** are very similar, providing confidence in assessment of the absorption spectra even if minor impurities are present in the latter.

Table S1. Predicted and observed Elemental analysis data for compounds (and precursors)

Compound	C predicted	C observed	H predicted	H observed	N predicted	N observed
**Ce_6**	32.96	31.95	5.16	4.56	0	0.21
**Ce_{40}**	13.73	13.25	1.64	1.61	0.59	1.15
**Ce_{100}**	24.99	25.98	1.81	1.71	0.41	0.51
**Ce_{24}^{Ph}** *(+3py)	36.18	36.30	2.39	2.18	1.21	1.19
**Ce_{24}^{PhMe}**	37.55	37.46	2.88	2.61	0.67	0.97
**Ce_{24}^{PhtBu}**	43.88	43.23	4.40	4.42	0.59	0.46
**Ce_{24}^{PhF}** *(+1py)	33.15	33.67	1.81	1.63	0.82	0.82
**Ce_{24}^{But}**	24.45	26.84	3.49	3.10	0.81	0.49
**Ce_{24}^{Hex}**	31.13	29.94	4.68	4.54	0.73	0.26
**Ce_{24}^{PhNH2}**	33.10	33.06	2.51	2.68	5.71	7.47
**Ce_{24}^{PhOMe}**	35.50	35.15	2.73	2.60	0.64	0.59
**Ce_{24}^{Ph1Nap}** *(+6 py)	46.23	46.56	2.74	2.45	1.42	1.48
**Ce_{24}^{Ph2Nap}** *(+2 py)	44.73	42.43	2.55	2.40	0.60	0.93
**$[Ce(O_2CCH_2{}^tBu)_3(L)]_\infty$** #(L = 0.4 py/0.6 H_2O coordinated)	45.49	45.66	6.91	6.70	1.06	1.57
$Ce(NO_3)_3.6(H_2O)$	*0*		*2.79*		*9.68*	
$[NH_4]_2Ce(NO_3)_6$	*0*		*1.47*		*20.44*	

The low angle data of the PXRD patterns of dry powders provides little value due to loss of crystallinity and/or shrinking of the unit cell upon solvent loss (see Supporting Figures), to address this, samples of three novel compounds **Ce_{24}^{PhOMe}**, **Ce_{24}^{PhNH2}** and **Ce_{24}^{PhtBu}** were prepared and directly analysed as microcrystalline powders loaded into capiliaries along with the reaction solution using Synchrotron diffraction methods (Figs S15, S18, S19). This confirms that the bulk materials prepared in solution are highly crystalline and show a good match to the simulated

powder pattern from single crystal data (albeit with minor impurity phases observed in capillary measurements in some cases), providing further confidence in the preparation of bulk materials using these synthetic methods.

Furthermore, there is value in the high angle diffraction patterns (Fig S1), which originates from diffraction from within the core of individual molecules. As the clusters contain a core comparable to fluorite CeO_2, they exhibit a diffraction pattern consistent with tiny CeO_2 nanoparticles at higher two theta. The pattern is clearest for Ce_{100} with the largest CeO_2 core, Scherrer analysis for Ce_{100} (Fig S1) estimates a particle size of 1.9 nm in excellent agreement with the size of the Ce_{100} core (pseudo-ellipsoidal with dimensions 1.7 x 1.9 x 2.3 nm).[55a]

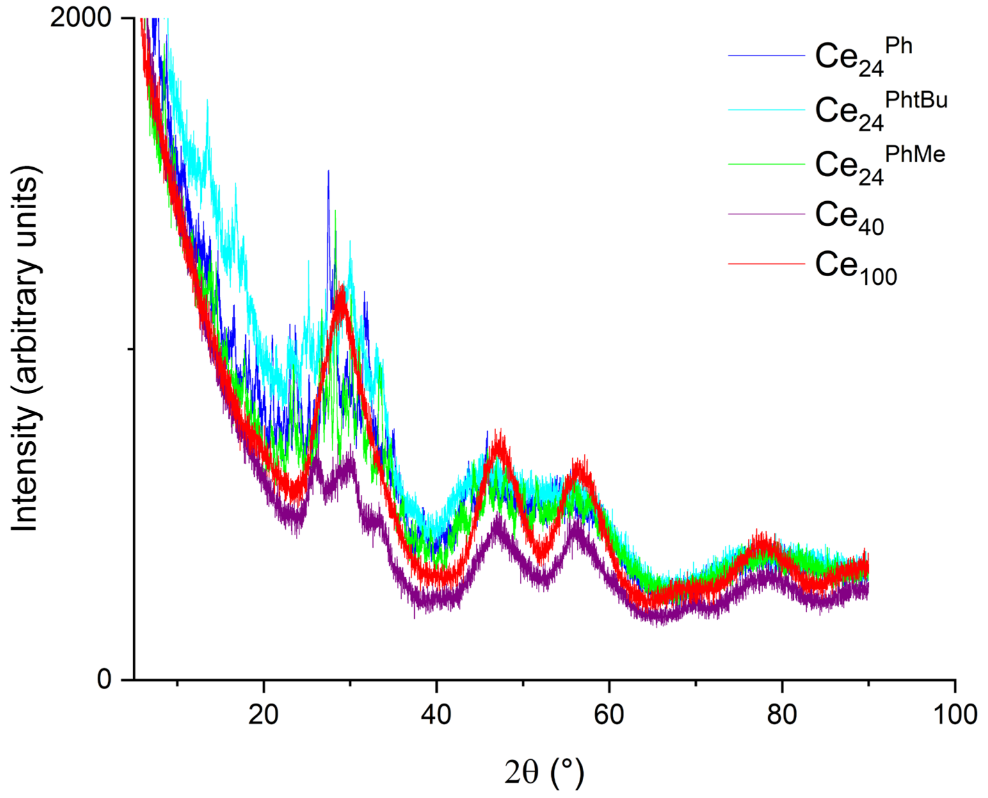

Pos. [°2θ]	FWHM	Grain Size (angstrom)
**28.6407**	4.086084	2.01
**31.7987**	4.166796	1.98
**47.2823**	4.400728	1.97
**56.6431**	4.896086	1.84
**77.5486**	6.630821	1.54
**average**		1.87

Figure S1. Powder X-ray diffraction pattern of different sized Ce-oxo clusters at high angles, revealing the expected diffraction signals that originate from the core CeO_2 fluorite lattice, confirming these are species may be described as molecular nanoparticles of CeO_2. Table below shows Scherrer analysis of **Ce_{100}** conducted using Highscore Plus.

None of the recorded PXRD patterns for Ce-oxo clusters show evidence of crystalline cerium nitrate starting materials. It is possible that Ce(III)carboxylates (potentially with coordinated pyridine) could be present as minor phases, although no evidence is found in the diffraction data, and it is noteworthy that these are colourless or pale-yellow compounds which absorb from >2.7 eV and will not affect the analysis of absorption onset (Fig. S26-27)

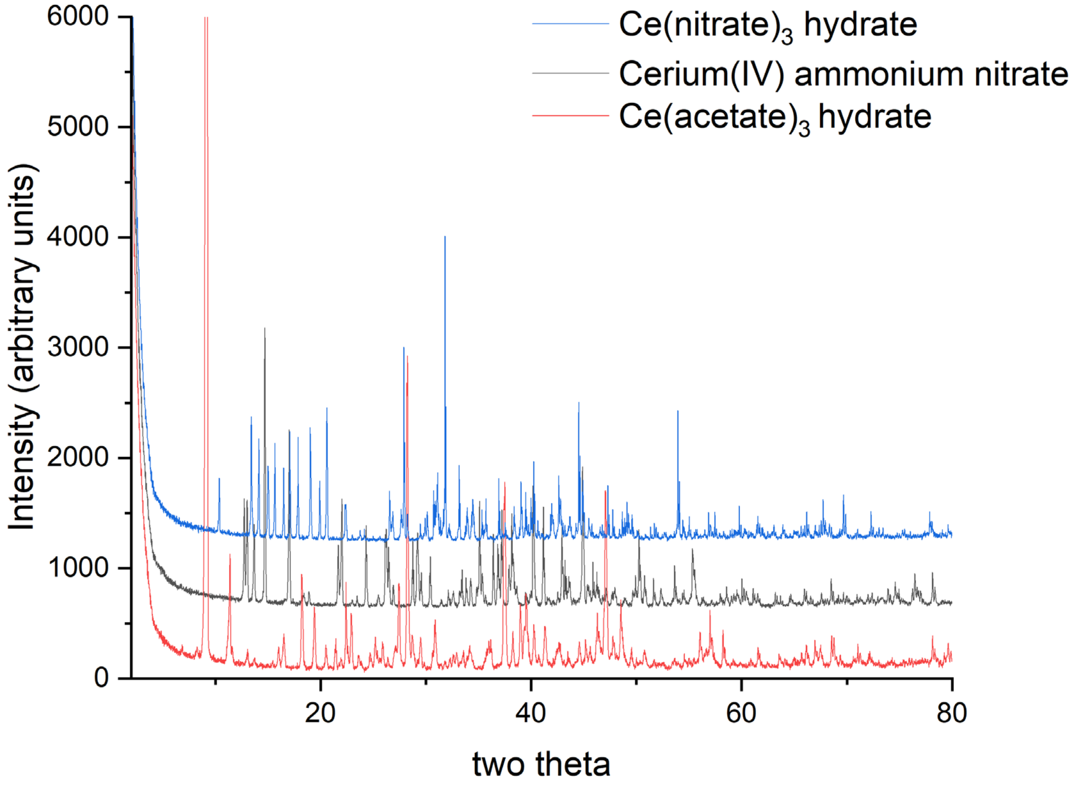

Figure S2. PXRD patterns of Ce starting materials and commercial Ce(acetate)$_3$ hydrate

Supporting Figures and Tables

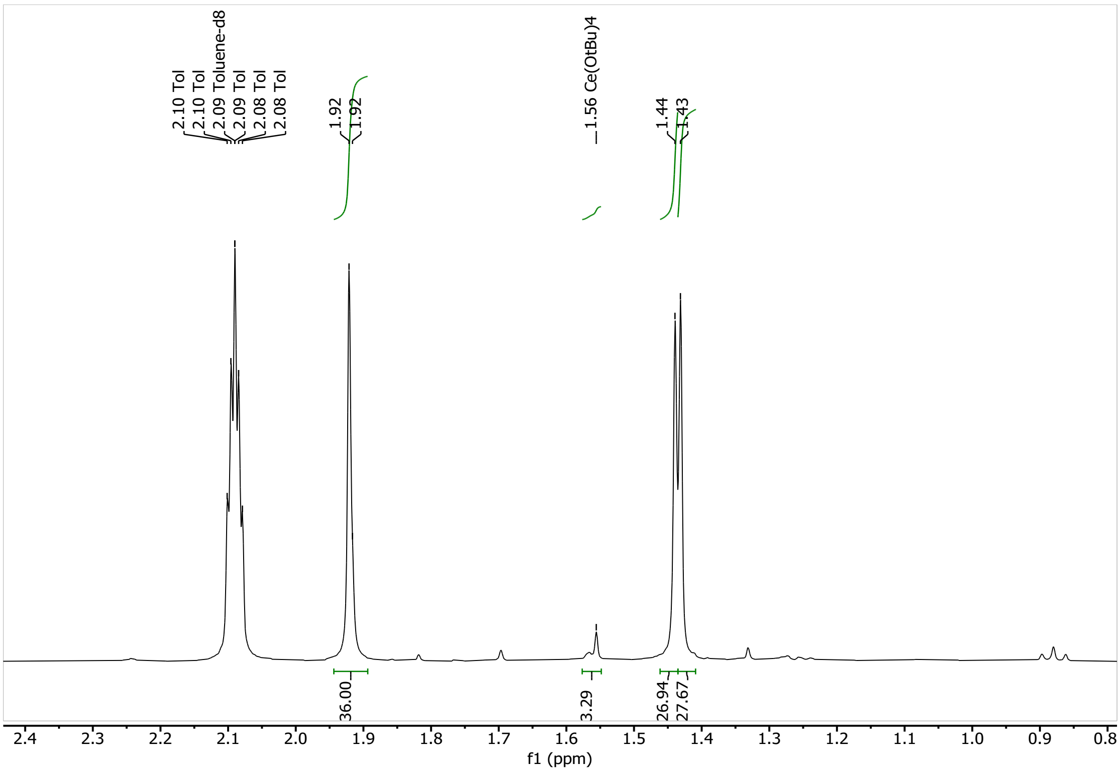

Figure S3. ^{1}H NMR spectrum of **Ce_3** in d_8 toluene. Note small quantity of the precursor $Ce(O^tBu)_4$.THF located at 1.56 ppm.

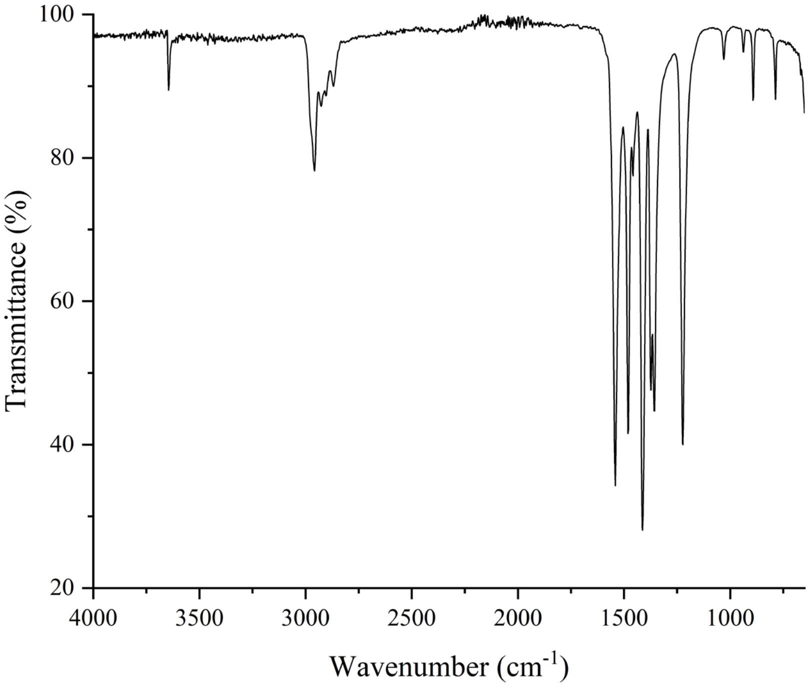

Figure S4. ATR-FTIR spectrum of **Ce_6**

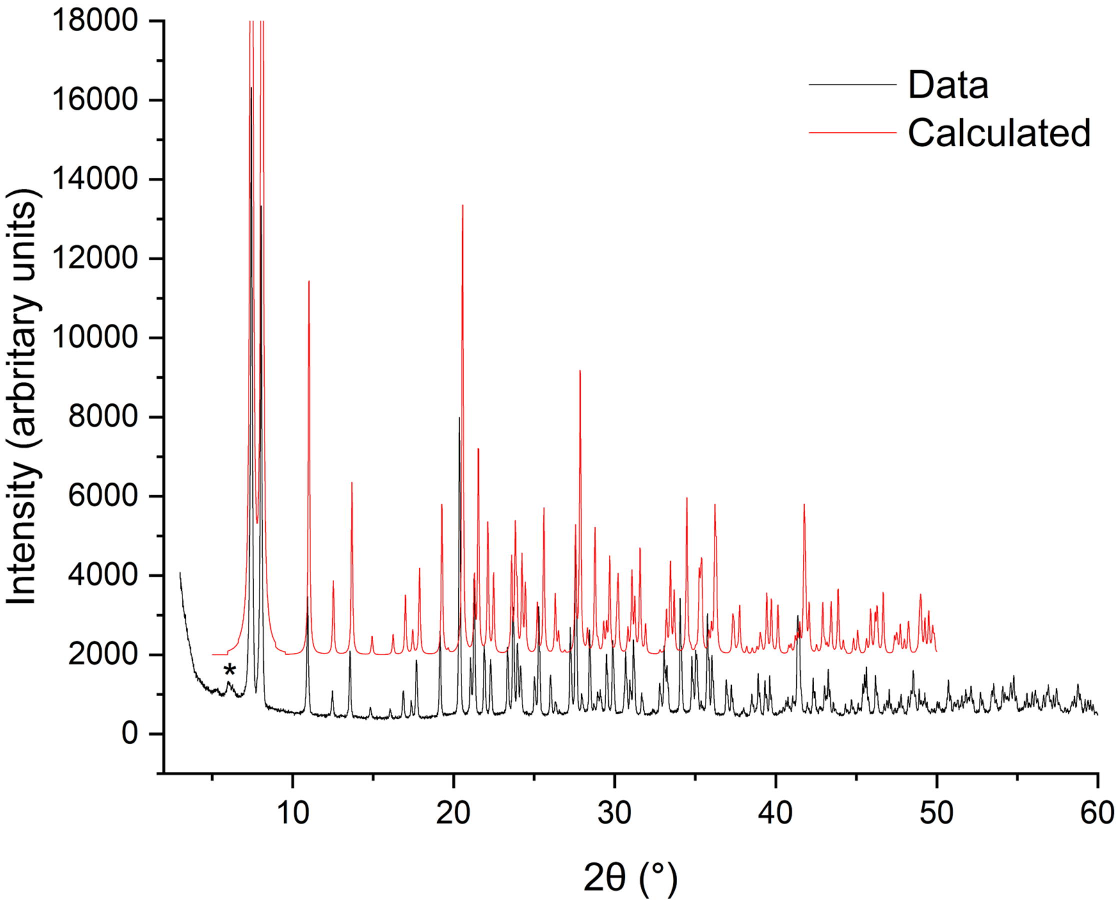

Figure S5. PXRD pattern of **Ce_6** at room temperature compared to calculated pattern derived from the 100 K SCXRD structure.[59] * Unknown impurity phase, consistent with a larger unit cell that may indicate the presence of a small amount of larger Ce-oxo-pivalate clusters present.

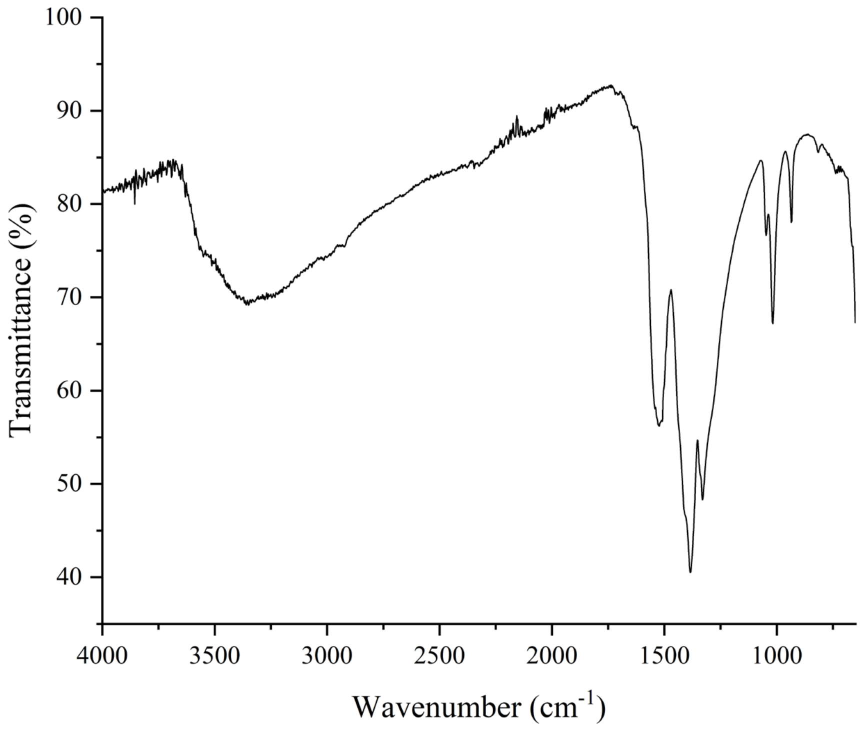

Figure S6. ATR-FTIR spectrum of **Ce_{40}**

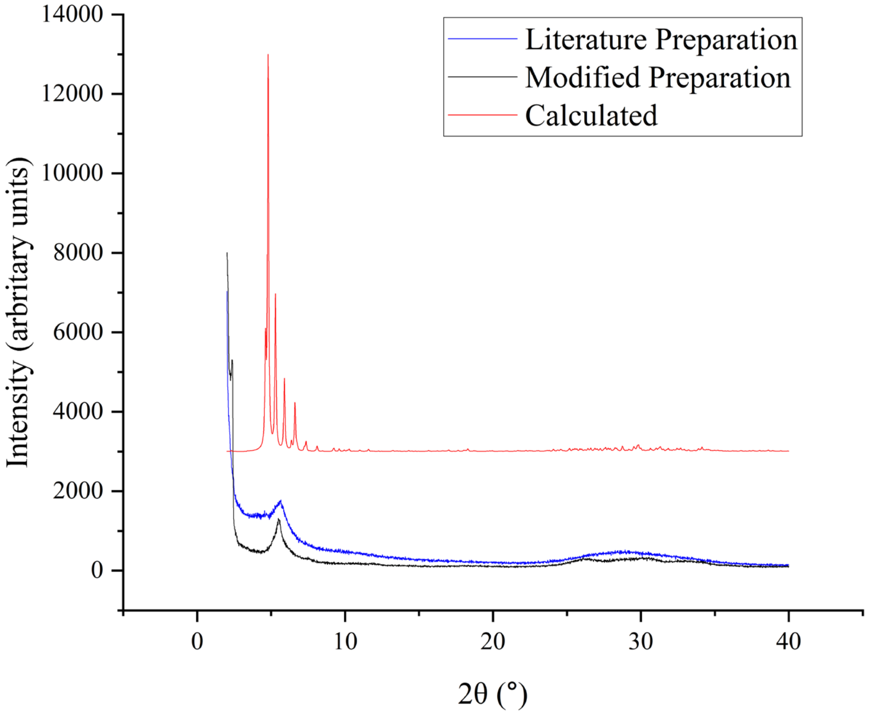

Figure S7 – PXRD pattern of **Ce_{40}** at room temperature, prepared for this study, compared to calculated pattern derived from the 100 K SCXRD structure and also a sample prepared from the previously reported literature route.[55b]

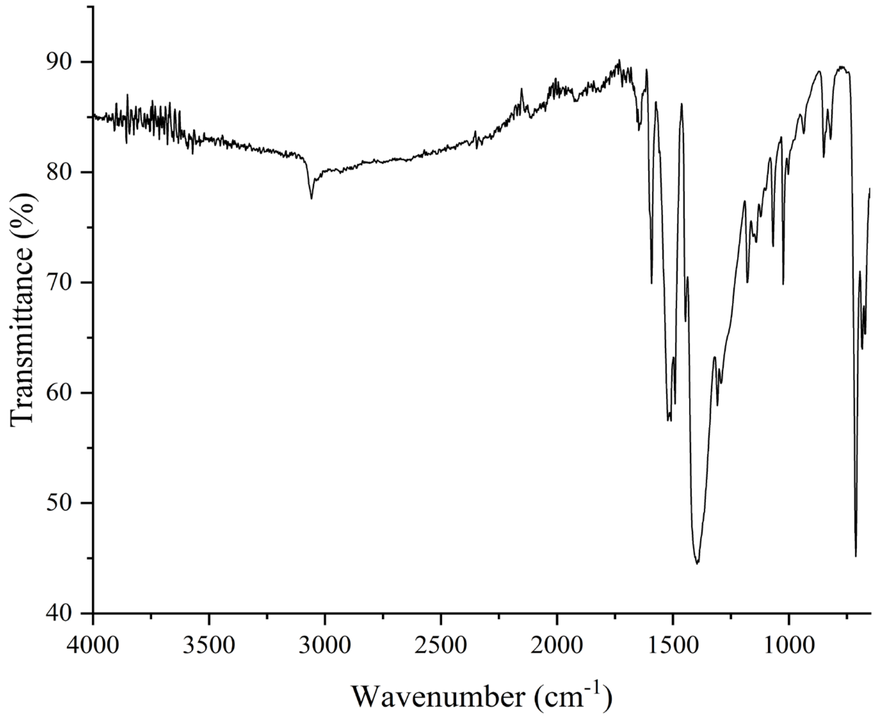

Figure S8. ATR-FTIR spectrum of **Ce_{100}**

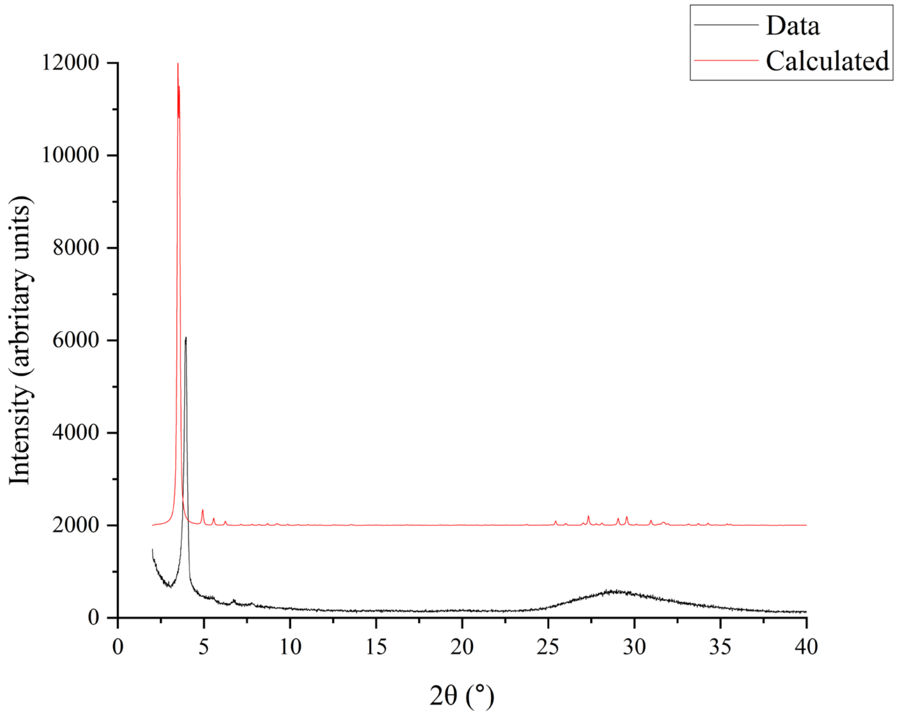

Figure S9. PXRD pattern of **Ce_{100}** at room temperature compared to calculated pattern derived from the 100 K SCXRD structure.[55a]

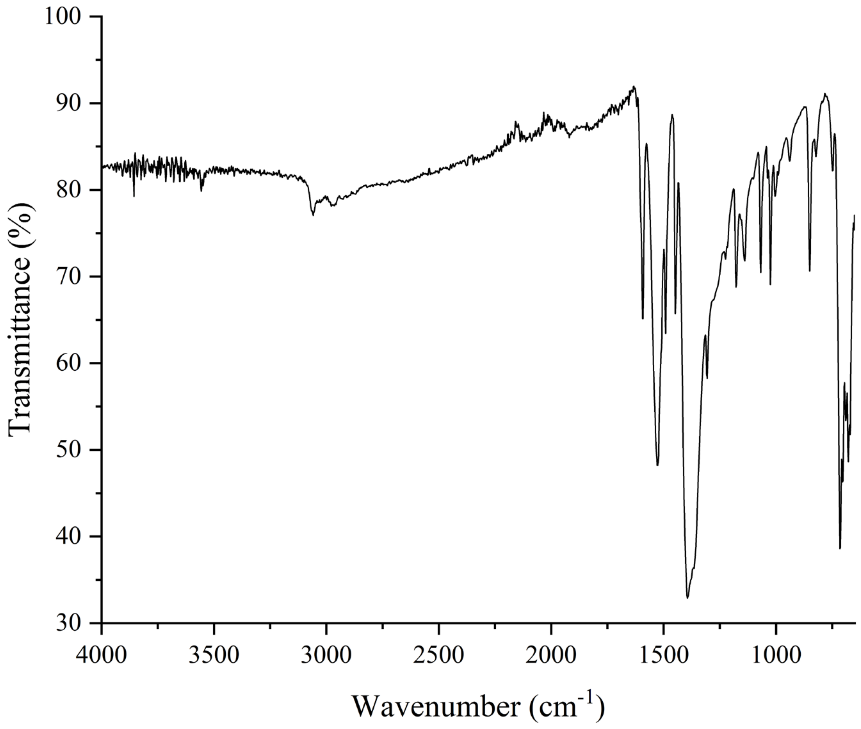

Figure S10. ATR-FTIR spectrum of **Ce_{24}^{Ph}**

Table S2 – Bond valence sum calculations for **Ce_{24}^{Ph}** using Ce(IV) or Ce(III) average bond distance parameters.[60] Proposed oxidation state shown in bold.

Atom	Coordination number	Multiplicity of Ce Site	Ce(IV) test	Ce(III) test
Ce1	8	2	**3.95**	4.55
Ce2	8	2	**3.65**	4.17
Ce3	8	2	**3.64**	4.16
Ce4	8	2	**3.54**	4.08
Ce5	9	2	**3.88**	4.48
Ce8	8	2	**3.45**	3.98
Ce11	10	2	2.65	**3.06**
Ce12	9	2	**3.89**	4.49
Ce13	9	2	**3.79**	4.38
Ce15	8	2	**3.81**	4.40
Ce20	8	2	**3.88**	4.48
Ce21	9	2	**3.81**	4.39

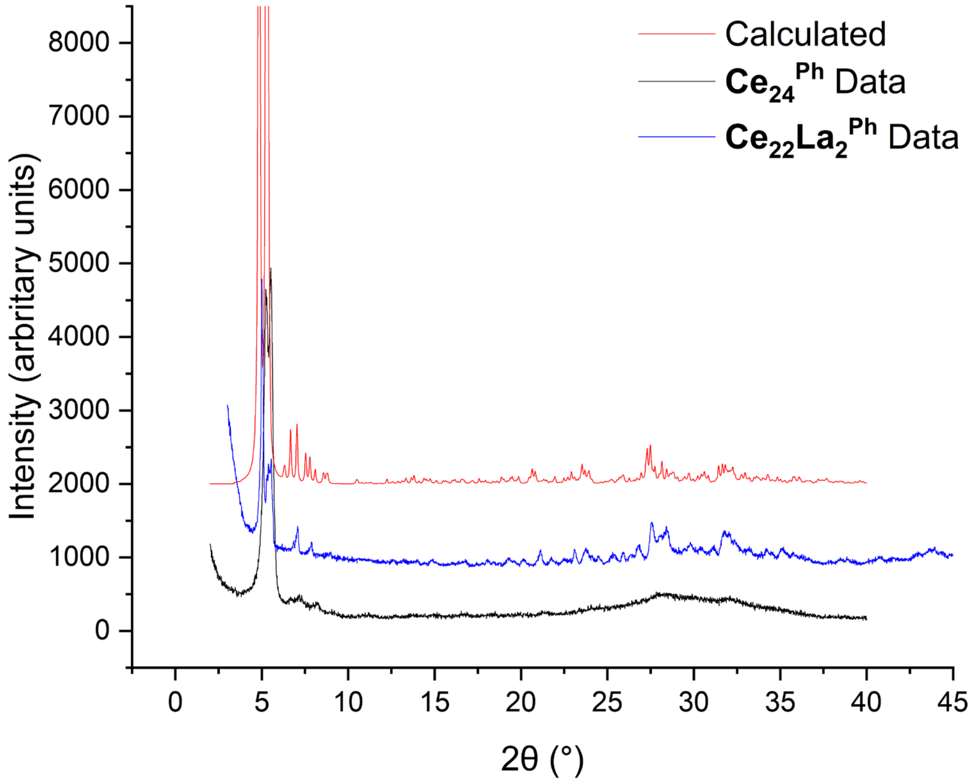

Figure S11. PXRD pattern of $\mathbf{Ce_{24}^{Ph}}$ and $\mathbf{Ce_{22}La_2^{Ph}}$ at room temperature compared to calculated pattern derived from the 100 K SCXRD structure.

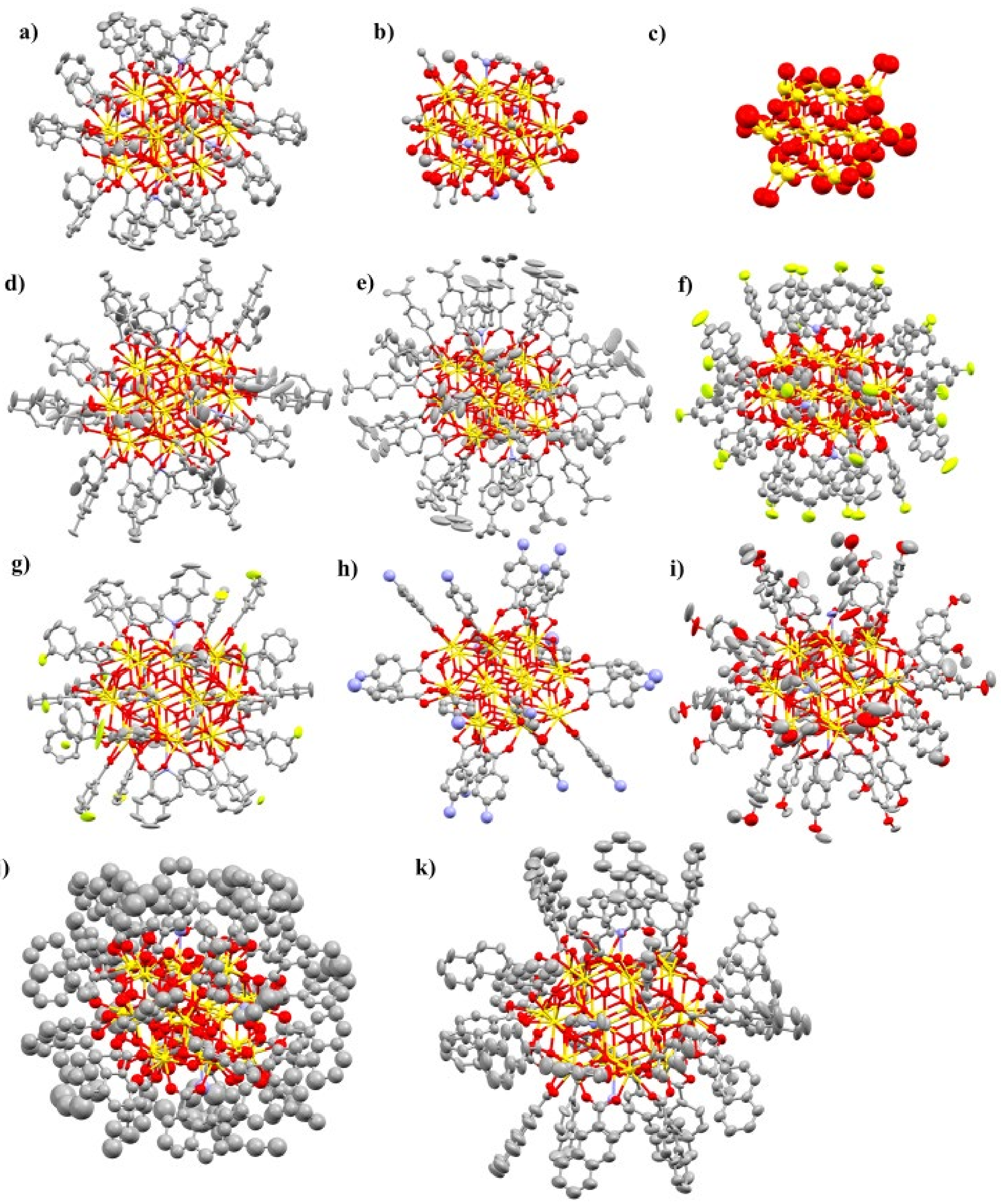

Figure S12. Single crystal X-ray structures of Ce_{24} clusters; drawn as thermal ellipsoids at 50% probability with H atoms omitted for clarity, with cerium (gold), oxygen (red) nitrogen (blue), carbon (grey), and fluorine (lime). In order a-k the molecules shown are; a) $\mathbf{Ce_{24}^{Ph}}$, b) $\mathbf{Ce_{24}^{But}}$,c) $\mathbf{Ce_{24}^{Hex}}$, d) $\mathbf{Ce_{24}^{PhMe}}$, e) $\mathbf{Ce_{24}^{PhtBu}}$, f) $\mathbf{Ce_{24}^{PhF}}$, g) $[Ce_{24}O_{28}(OH)_8(O_2C\text{-meta-}C_6H_4F)_{30}(py)_4](py)_{22}$ (single crystal structure reported only, synthesised as for $\mathbf{Ce_{24}^{PhF}}$, but with meta-fluorobenzoic acid), h) $\mathbf{Ce_{24}^{PhNH2}}$, i) $\mathbf{Ce_{24}^{PhOMe}}$, j) $\mathbf{Ce_{24}^{1Nap}}$, k) **and** $\mathbf{Ce_{24}^{2Nap}}$. The total ligand coordination shell could not be resolved for $\mathbf{Ce_{24}^{But}}$, $\mathbf{Ce_{24}^{Hex}}$, $\mathbf{Ce_{24}^{PhNH2}}$, and $\mathbf{Ce_{24}^{1Nap}}$.

Table S3 – BVS calculations for $\mathbf{Ce_{24}^{PhMe}}$, proposed oxidation state shown in bold.

Atom label Molecule 1	Coordination number	Multiplicity of Ce Site	Ce(IV) test	Ce(III) test
Ce1	8	2	**3.90**	4.51
Ce2	8	2	**3.67**	4.19
Ce3	8	2	**3.64**	4.16
Ce4	9	2	**3.82**	4.41
Ce5	9	2	**3.74**	4.31
Ce8	9	2	**3.53**	4.08
Ce21	9	2	**3.83**	4.42
Ce22	10	2	2.61	**3.02**
Ce23	9	2	**3.59**	4.14
Ce26	9	2	**3.89**	4.49
Ce38	8	2	**3.77**	4.35
Ce43	8	2	**3.87**	4.46
Molecule 2				
Ce193	8	2	**3.98**	4.59
Ce194	8	2	**3.69**	4.22
Ce195	8	2	**3.63**	4.15
Ce196	9	2	**3.83**	4.42
Ce197	9	2	**3.83**	4.42
Ce200	9	2	**3.67**	4.24
Ce213	10	2	2.60	**3.00**
Ce214	9	2	**3.84**	4.44
Ce215	8	2	**3.54**	4.08
Ce217	8	2	**3.86**	4.46
Ce222	8	2	**3.82**	4.41
Ce223	8	2	**3.78**	4.36

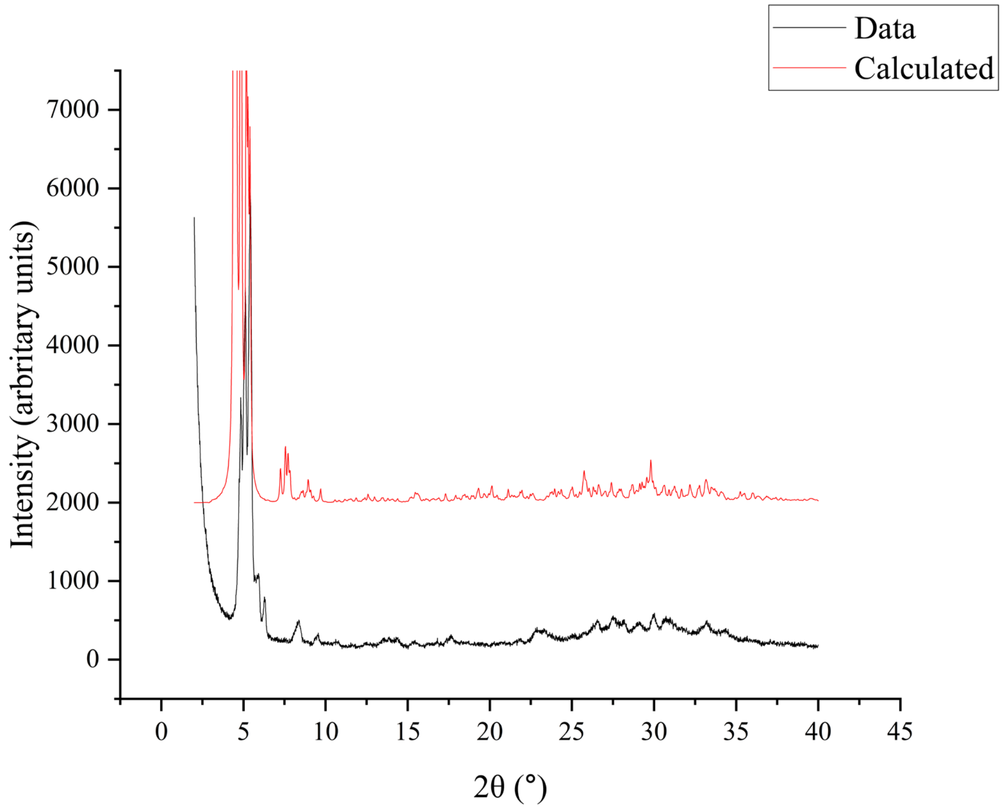

Figure S13. PXRD pattern of **Ce_{24}^{PhMe}** at room temperature compared to calculated pattern derived from the 100 K SCXRD structure.

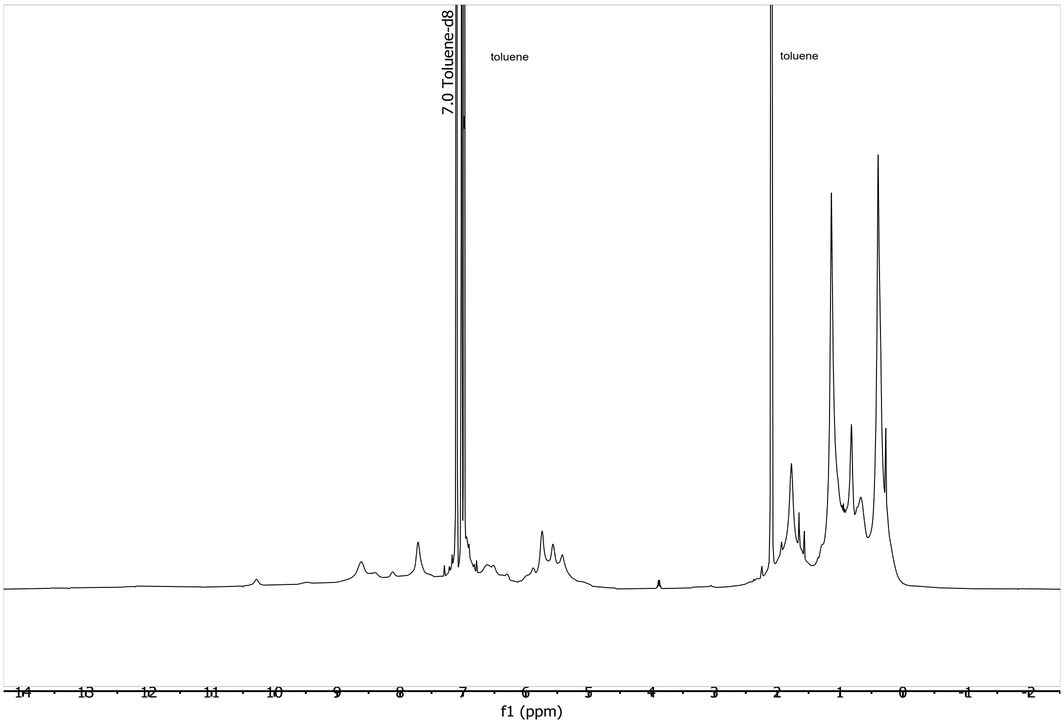

Figure S14. ^{1}H NMR spectrum of **Ce_{24}^{PhtBu}** in d_8 toluene

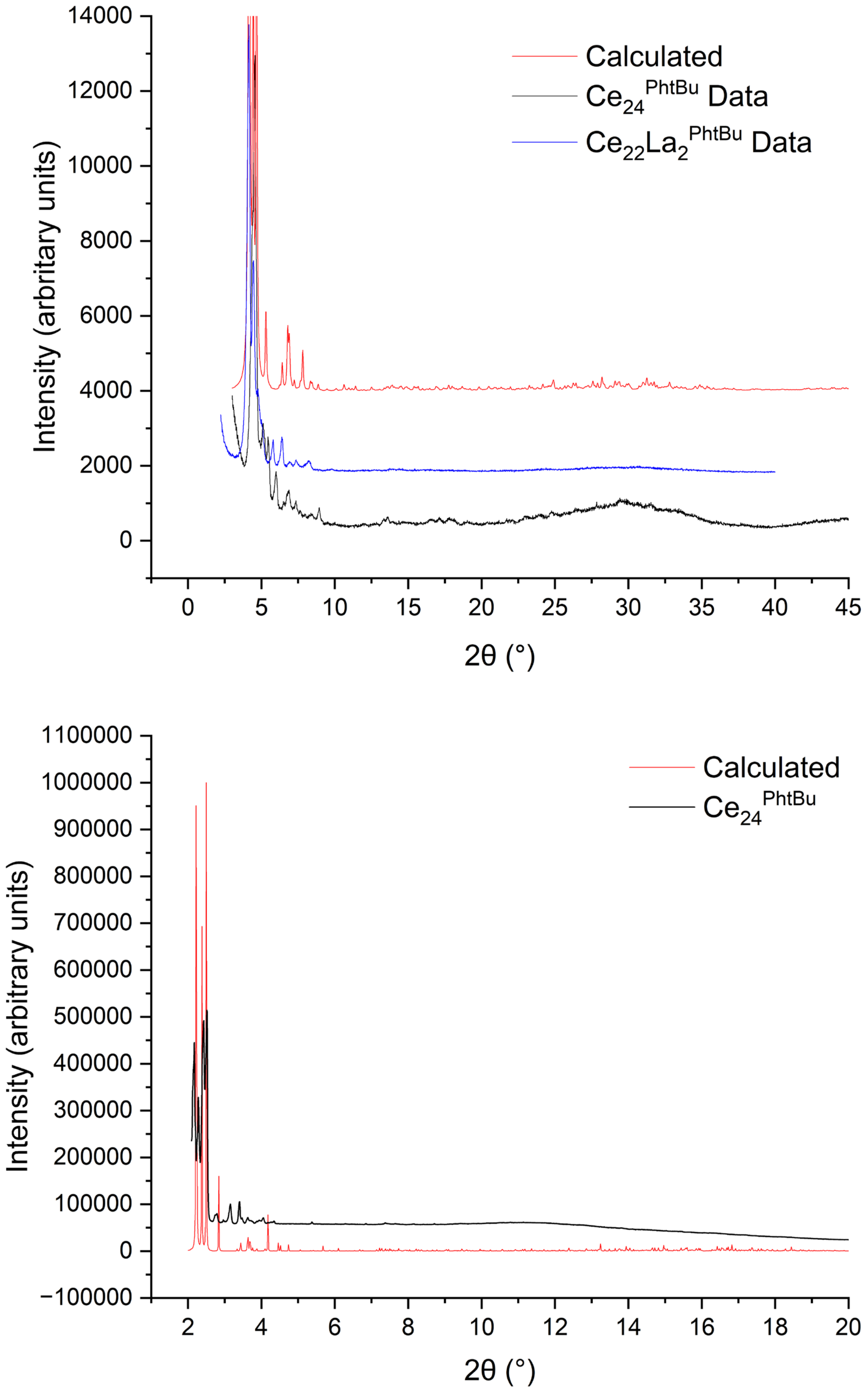

Figure S15. PXRD pattern of **Ce_{24}^{PhtBu}** at room temperature compared to calculated pattern derived from the approximate 100 K SCXRD structure (note difficulty of accurately solving disordered tBu groups and solvents in the structure). Above) dry powder, also with **$Ce_{22}La_2^{PhtBu}$**; Below) microcrystalline batch directly synthesised and measured within a capillary (at synchrotron) also containing the reaction solution.

Table S4. BVS calculations for $\mathbf{Ce_{24}}^{PhF}$, proposed oxidation state shown in bold.

Atom	Coordination number	Multiplicity of Ce Site	Ce(IV) test	Ce(III) test
Ce1	9	1	**3.82**	4.41
Ce2	8	1	**3.74**	4.32
Ce3	10	1	2.58	**2.98**
Ce4	9	1	**3.86**	4.45
Ce5	9	1	**3.78**	4.37
Ce6	9	1	**3.70**	4.19
Ce7	8	1	**3.69**	4.23
Ce8	8	1	**3.93**	4.54
Ce9	9	1	**3.78**	4.36
Ce10	8	1	**3.84**	4.43
Ce11	8	1	**3.63**	4.15
Ce12	9	1	**3.82**	4.41
Ce13	8	1	**3.81**	4.40
Ce14	8	1	**3.65**	4.17
Ce15	9	1	**3.95**	4.55
Ce16	8	1	**3.60**	4.11
Ce17	8	1	**3.92**	4.53
Ce18	9	1	**3.92**	4.52
Ce19	9	1	**3.82**	4.41
Ce20	9	1	**3.93**	4.53
Ce21	8	1	**3.71**	4.23
Ce22	9	1	**3.80**	4.39
Ce23	9	1	**4.03**	4.65
Ce24	10	1	2.66	**3.07**

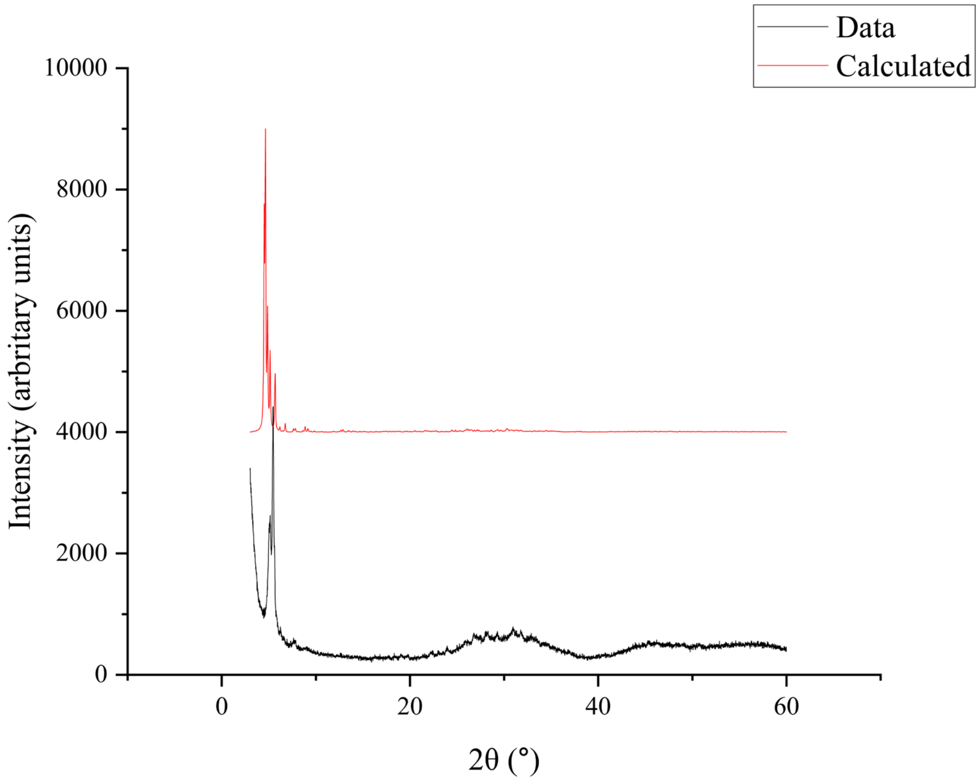

Figure S16. PXRD pattern of $\mathbf{Ce_{24}}^{PhF}$ at room temperature compared to calculated pattern derived from the 100 K SCXRD structure.

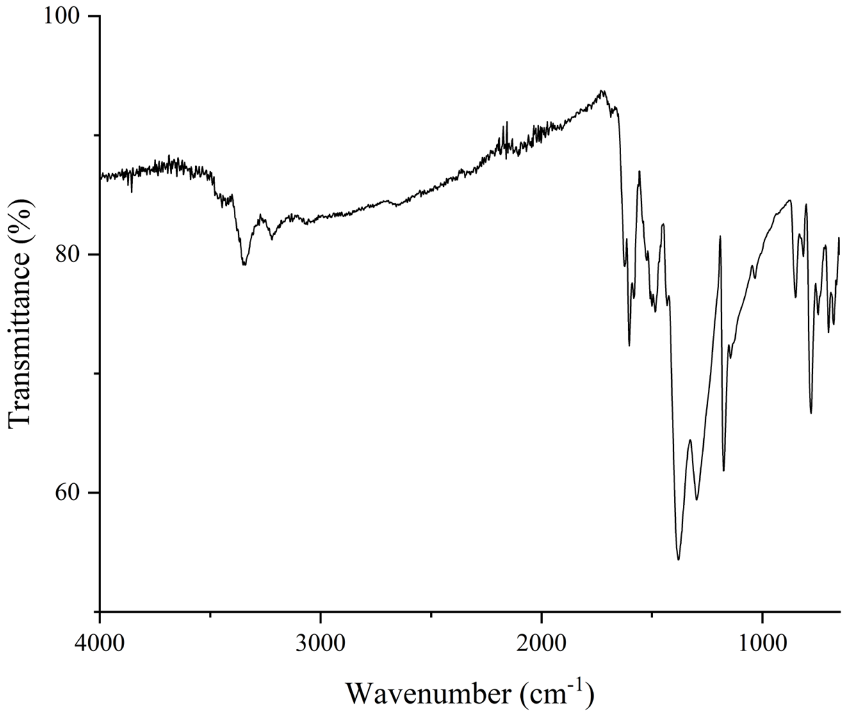

Figure S17. ATR-FTIR spectrum of $\mathbf{Ce_{24}}^{PhNH2}$

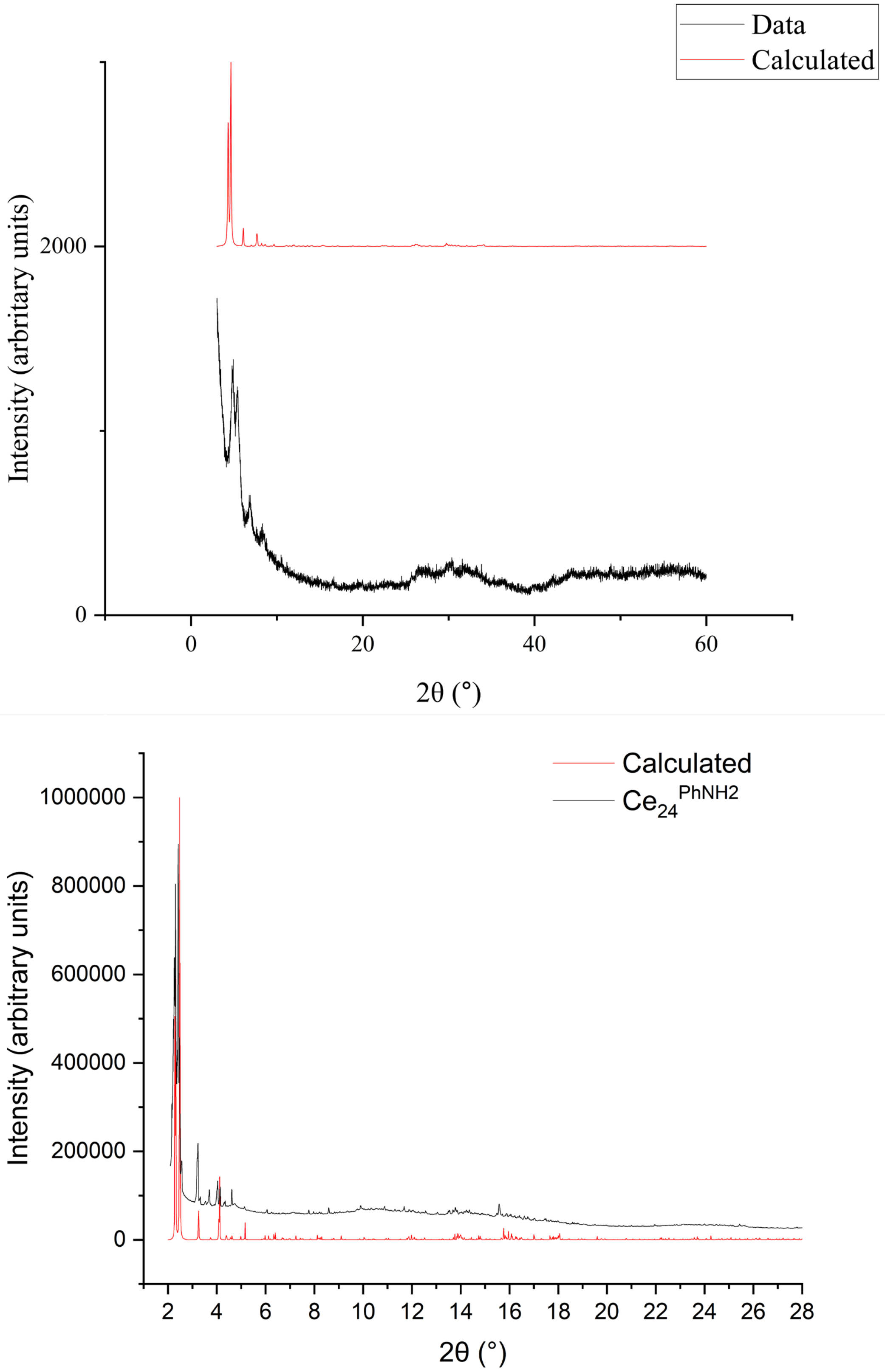

Figure S18. PXRD pattern of $\mathbf{Ce_{24}^{PhNH2}}$ at room temperature compared to calculated pattern derived from the approximate 100 K SCXRD structure (note difficulty of accurately solving disordered tBu groups and solvents in the structure). Above) dry powder; below) microcrystalline batch directly synthesised measured within a capillary (at synchrotron) also containing the reaction solution.

Table S5 – BVS calculations for $\mathbf{Ce_{24}^{PhOMe}}$, proposed oxidation state shown in bold.

Atom	Coordination number	Multiplicity of Ce Site	Ce(IV) test	Ce(III) test
Ce1	8	1	**3.86**	4.45
Ce2	8	1	**3.78**	4.37
Ce3	8	1	**3.75**	4.28
Ce4	8	1	**3.73**	4.26
Ce6	9	1	**3.96**	4.57
Ce7	8	1	**4.07**	4.70
Ce9	8	1	**3.81**	4.35
Ce10	8	1	**3.79**	4.33
Ce12	8	1	**3.72**	4.29
Ce13	8	1	**3.81**	4.40
Ce15	9	1	**3.83**	4.42
Ce17	9	1	**3.87**	4.46
Ce19	10	1	2.64	**3.05**
Ce22	9	1	**3.81**	4.40
Ce23	8	1	**3.89**	4.49
Ce48	9	1	**3.83**	4.42
Ce51	9	1	**3.92**	4.52
Ce54	10	1	2.67	**3.08**
Ce55	9	1	**3.77**	4.35
Ce56	9	1	**3.89**	4.49
Ce60	8	1	**3.82**	4.41
Ce61	9	1	**3.84**	4.43
Ce111	9	1	**3.90**	4.50
Ce114	9	1	**3.68**	4.25

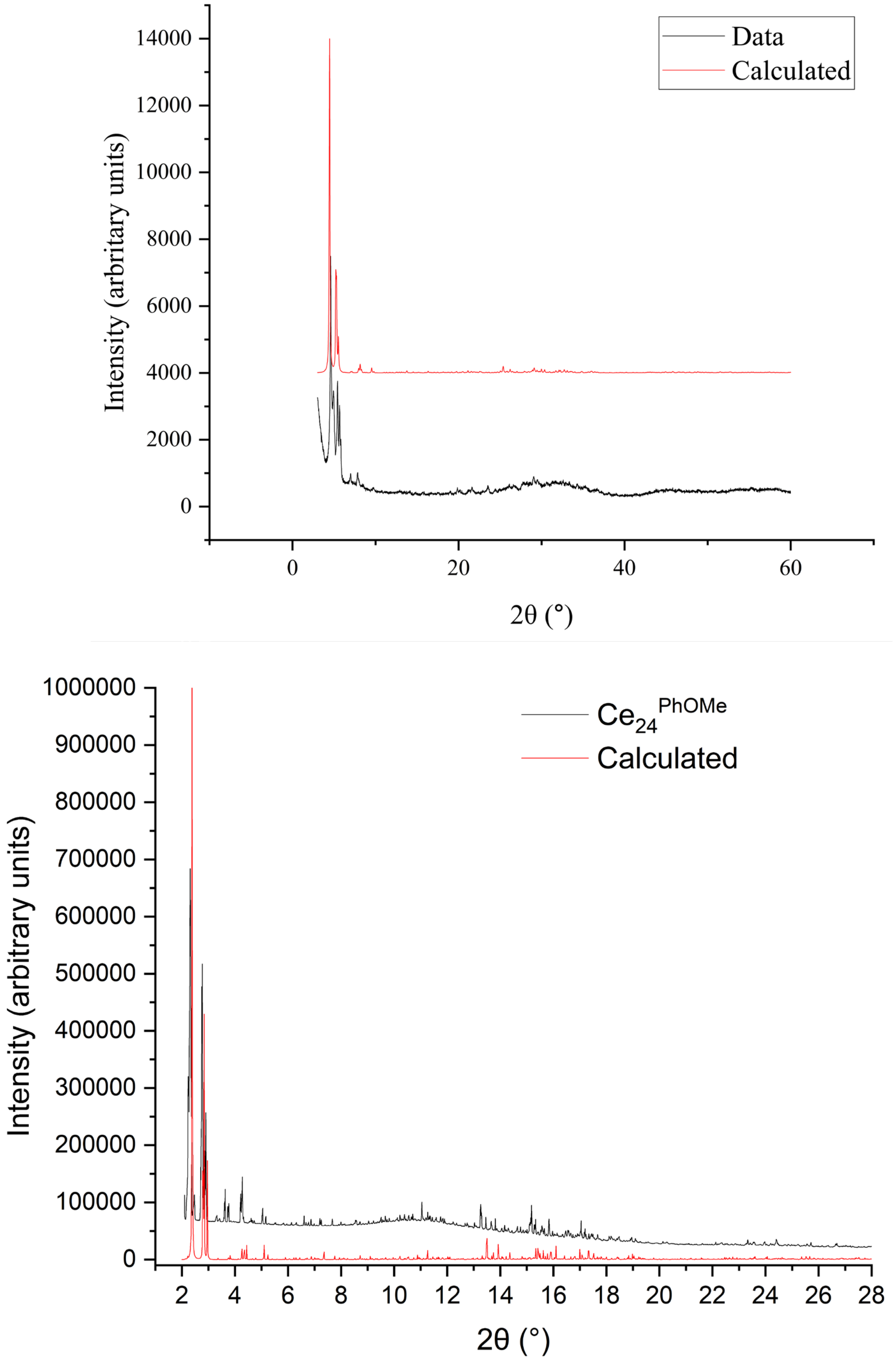

Figure S19. PXRD pattern of **Ce_{24}^{PhOMe}** at room temperature compared to calculated pattern derived from the 100 K SCXRD structure. Above) dry powder Below) microcrystalline batch directly synthesised measured within a capillary (at synchrotron) also containing the reaction solution.

Table S6. BVS calculations for $\mathbf{Ce_{24}^{1Nap}}$, proposed oxidation state shown in bold.

Atom	Coordination number	Multiplicity of Ce Site	Ce(IV) test	Ce(III) test
Ce1	9	1	**3.50**	4.04
Ce2	8	1	**3.55**	4.06
Ce3	9	1	**3.80**	4.38
Ce4	8	1	**4.00**	4.62
Ce6	9	1	**3.52**	4.06
Ce7	8	1	**3.68**	4.22
Ce9	9	1	**3.70**	4.27
Ce11	8	1	**3.76**	4.30
Ce12	9	1	**3.55**	4.10
Ce14	9	1	**3.67**	4.24
Ce16	9	1	**3.52**	4.06
Ce18	8	1	**3.76**	4.34
Ce19	10	1	2.52	**2.90**
Ce21	9	1	**3.41**	3.94
Ce22	9	1	**3.25**	3.76
Ce24	9	1	**3.58**	4.14
Ce26	9	1	**3.91**	4.47
Ce28	9	1	**3.70**	4.27
Ce30	9	1	**3.47**	4.00
Ce33	9	1	**3.81**	4.40
Ce36	9	1	**3.14**	3.62
Ce37	10	1	2.59	**2.99**
Ce132	9	1	**3.64**	4.20
Ce265	9	1	**3.83**	4.42

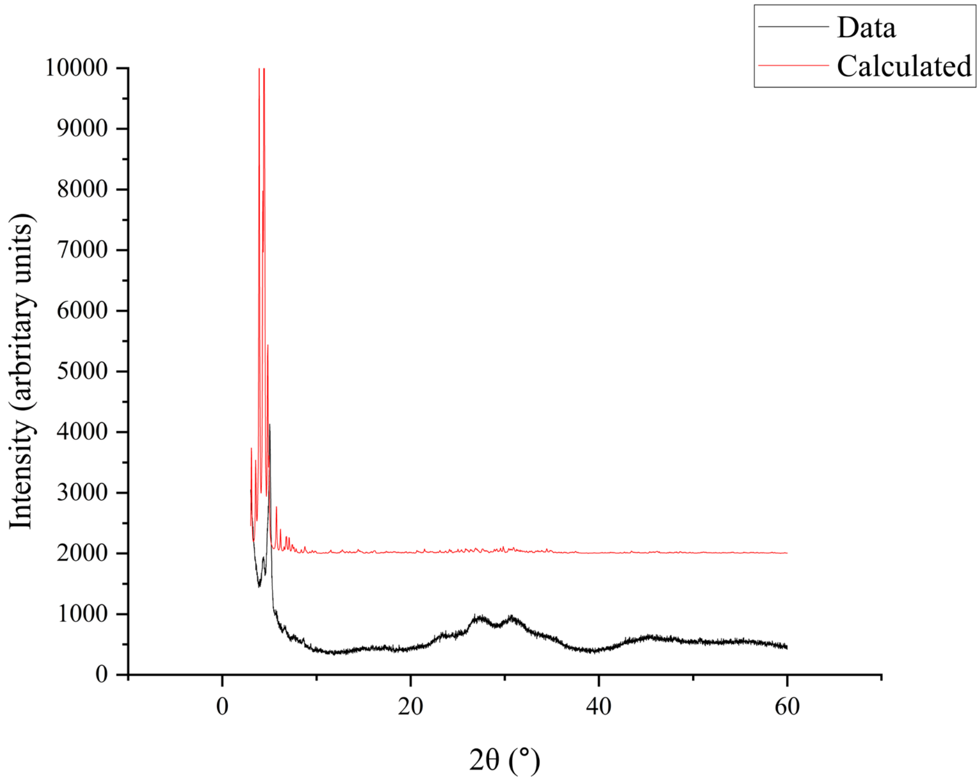

Figure S20. PXRD pattern of **Ce_{24}**^{1Nap} at room temperature compared to calculated pattern derived from the 100 K SCXRD structure.

Table S7. BVS calculations for **Ce_{24}**^{2Nap}, proposed oxidation state shown in bold.

Atom Abel	Coordination number	Multiplicity of Ce Site	Ce(IV) test	Ce(III) test
Ce1	10	1	2.65	**3.06**
Ce2	9	2	**3.82**	4.40
Ce3	9	2	**3.88**	4.50
Ce4	8	2	**3.83**	4.42
Ce5	8	1	**3.93**	4.53
Ce6	9	2	**3.82**	4.41
Ce7	8	2	**3.63**	4.14
Ce8	8	2	**3.63**	4.15
Ce9	8	2	**3.88**	4.47
Ce10	9	2	**3.64**	4.20
Ce11	9	2	**3.80**	4.38
Ce12	8	1	**4.00**	4.62
Ce13	9	2	**3.78**	4.36
Ce14	10	1	2.54	**2.93**

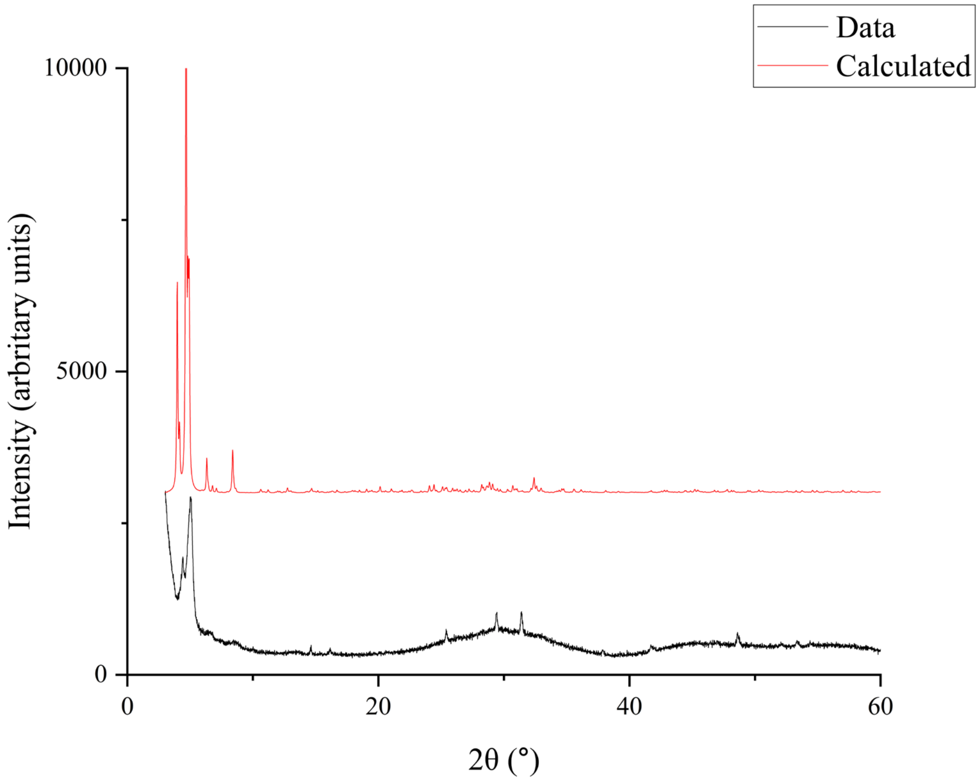

Figure S21. PXRD pattern of $\mathbf{Ce_{24}}^{\mathbf{2Nap}}$ at room temperature compared to calculated pattern derived from the 100 K SCXRD structure. An unknown impurity phase is likely present.

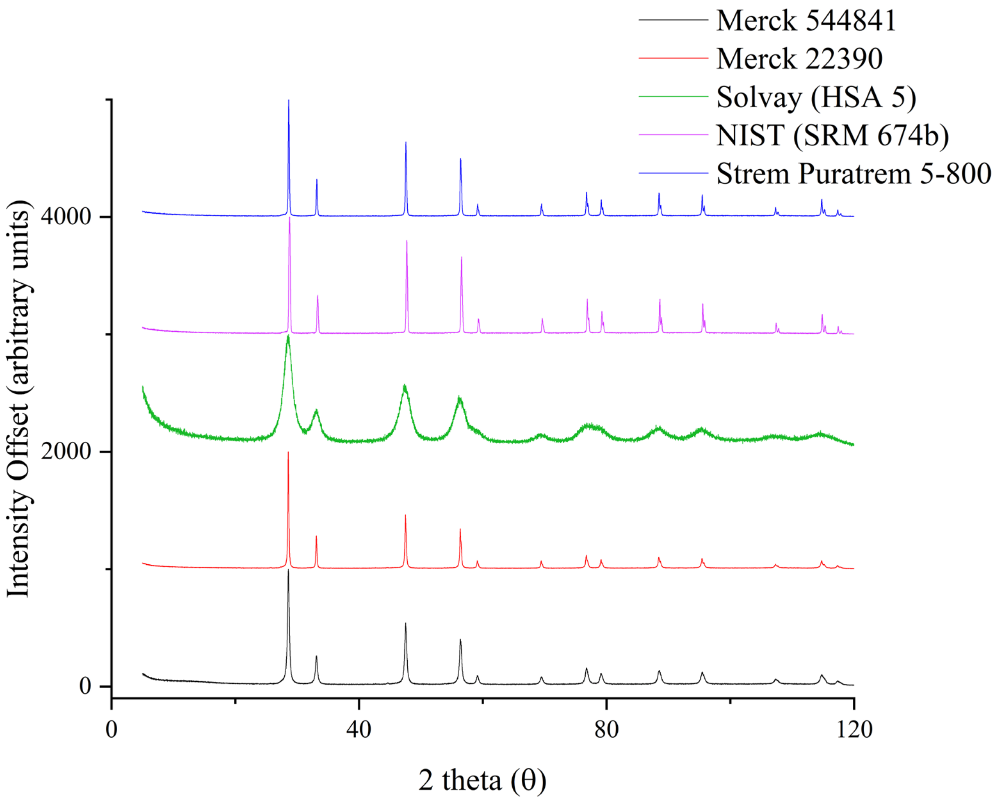

Figure S22. Powder X-ray diffractograms of commercially available ceria materials

Table S8. Scherrer analysis of CeO_2 materials (Fig S20), Range determined by analysis of all available peaks in PXRD diffractogram. Average generated by mean of all grain sizes.

Sample	Grain size range (nm)	Grain size average (nm)
NIST (SRM 674b)	54-106	76
Merck 22390	35-52	40
Merck 544841	16-23	21
Solvay (HSA 5)	3-6	5
Strem Puratrem 58-0800	62-75	68

Table S9. X-ray fluorescence spectroscopy data. Mass% does not include O.

Sample	Mass%					
	Ce	Al	Si	S	Cl	P
NIST (SRM 674b)	99.5	0.184	0.142	0.098	0	0.0567
Merck 22390	99.8	0.0554	0.0928	0.0270	0.0548	0
Merck 544841	99.7	0.0242	0.201	0.007	0.0648	0.007
Solvay (HAS 5)	99.9	0.0318	0.0916	0	0	0
CAN (Fluka)	99.8	0.0256	0.150	0	0	0
$Ce(NO_3)_3.6H_2O$ (Sigma Aldrich)	99.7	0.0382	0.164	0.0255	0.0445	0

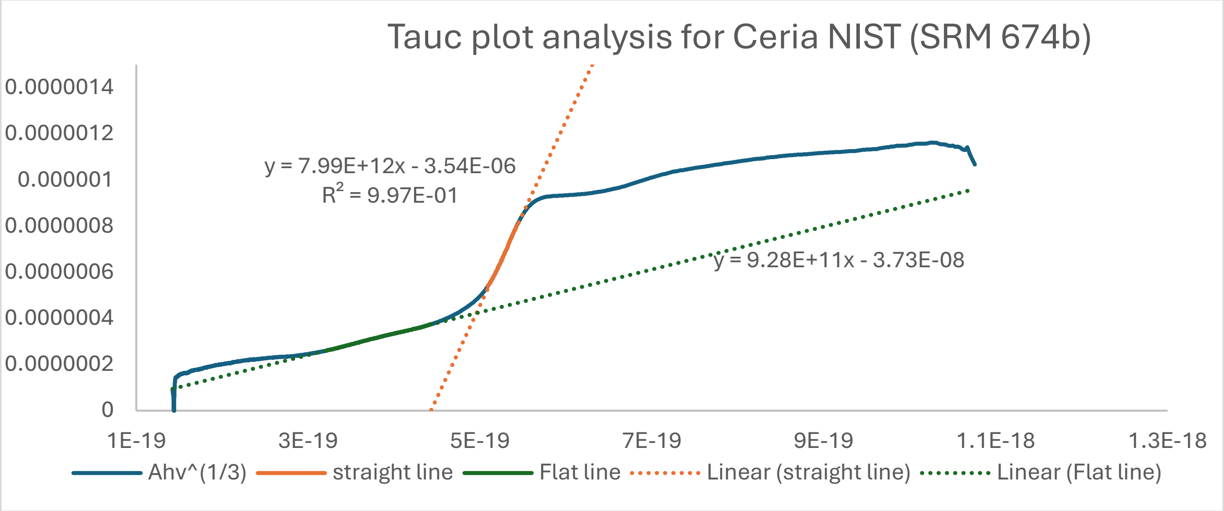
Tauc plot analysis for Ceria NIST (SRM 674b)
y = 7.99E+12x - 3.54E-06
R2 = 9.97E-01
y = 9.28E+11x - 3.73E-08
0.0000014
0.0000012
0.000001
0.0000008
0.0000006
0.0000004
0.0000002
0
1E-19
3E-19
5E-19
7E-19
9E-19
1.1E-18
1.3E-18
Ahv^(1/3)
straight line
Flat line
Linear (straight line)
Linear (Flat line)

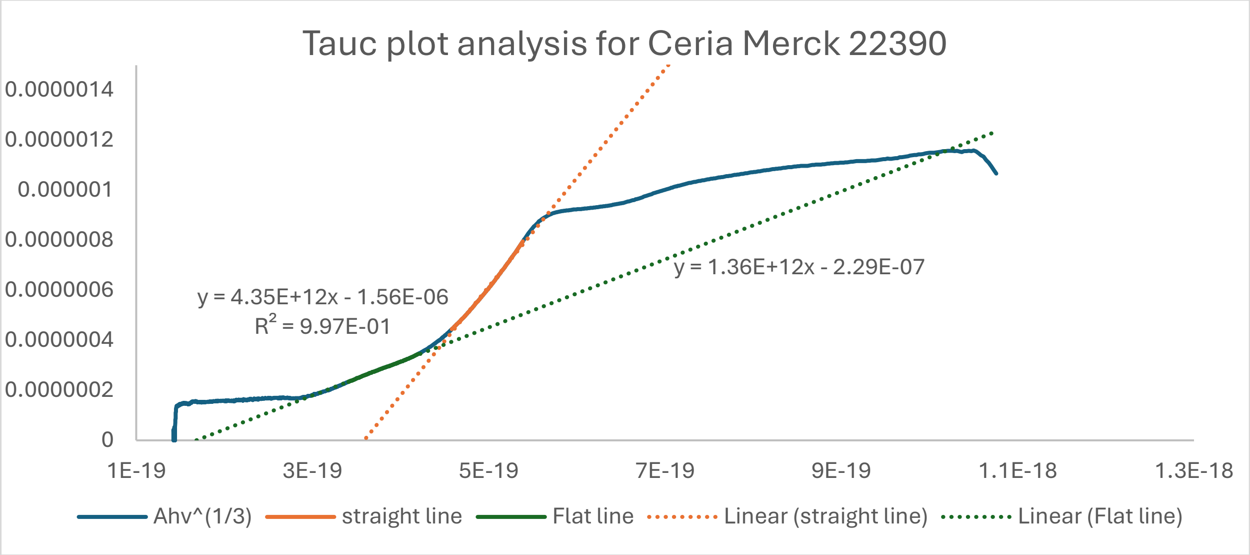
Tauc plot analysis for Ceria Merck 22390
y = 1.36E+12x - 2.29E-07
y = 4.35E+12x - 1.56E-06
R2 = 9.97E-01
0.0000014
0.0000012
0.000001
0.0000008
0.0000006
0.0000004
0.0000002
0
1E-19
3E-19
5E-19
7E-19
9E-19
1.1E-18
1.3E-18
Ahv^(1/3)
straight line
Flat line
Linear (straight line)
Linear (Flat line)

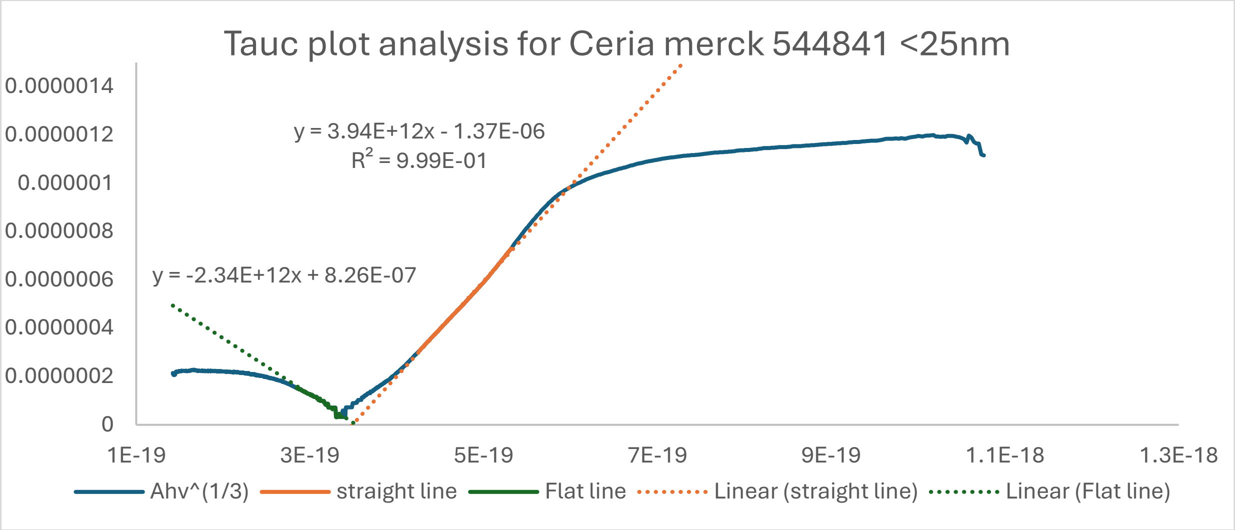
Tauc plot analysis for Ceria merck 544841 <25nm
y = 3.94E+12x - 1.37E-06
R2 = 9.99E-01
y = -2.34E+12x + 8.26E-07
0.0000014
0.0000012
0.000001
0.0000008
0.0000006
0.0000004
0.0000002
0
1E-19
3E-19
5E-19
7E-19
9E-19
1.1E-18
1.3E-18
Ahv^(1/3)
straight line
Flat line
Linear (straight line)
Linear (Flat line)

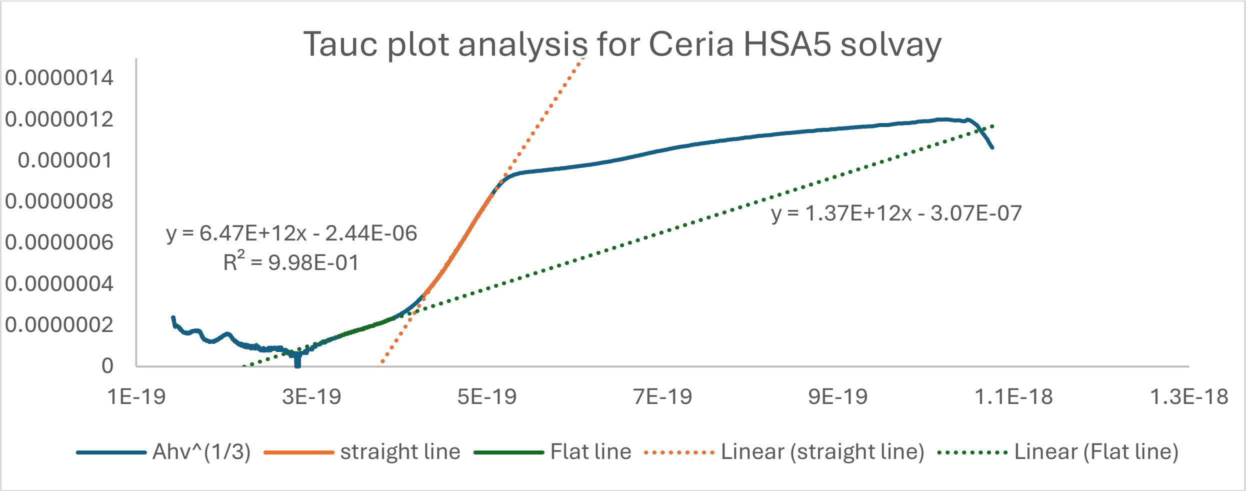
Tauc plot analysis for Ceria HSA5 solvay
0.0000014
0.0000012
0.000001
0.0000008
0.0000006
0.0000004
0.0000002
0
1E-19
3E-19
5E-19
7E-19
9E-19
1.1E-18
1.3E-18
y = 6.47E+12x - 2.44E-06
R² = 9.98E-01
y = 1.37E+12x - 3.07E-07
Ahv^(1/3)
straight line
Flat line
Linear (straight line)
Linear (Flat line)

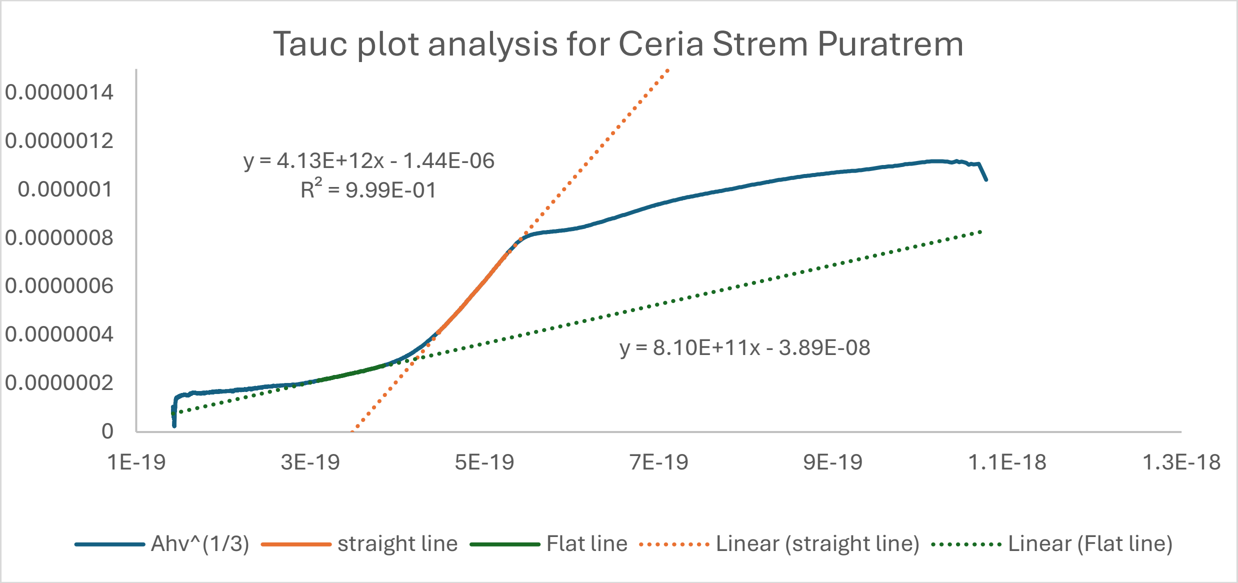
Tauc plot analysis for Ceria Strem Puratrem
0.0000014
0.0000012
0.000001
0.0000008
0.0000006
0.0000004
0.0000002
0
1E-19
3E-19
5E-19
7E-19
9E-19
1.1E-18
1.3E-18
y = 4.13E+12x - 1.44E-06
R² = 9.99E-01
y = 8.10E+11x - 3.89E-08
Ahv^(1/3)
straight line
Flat line
Linear (straight line)
Linear (Flat line)

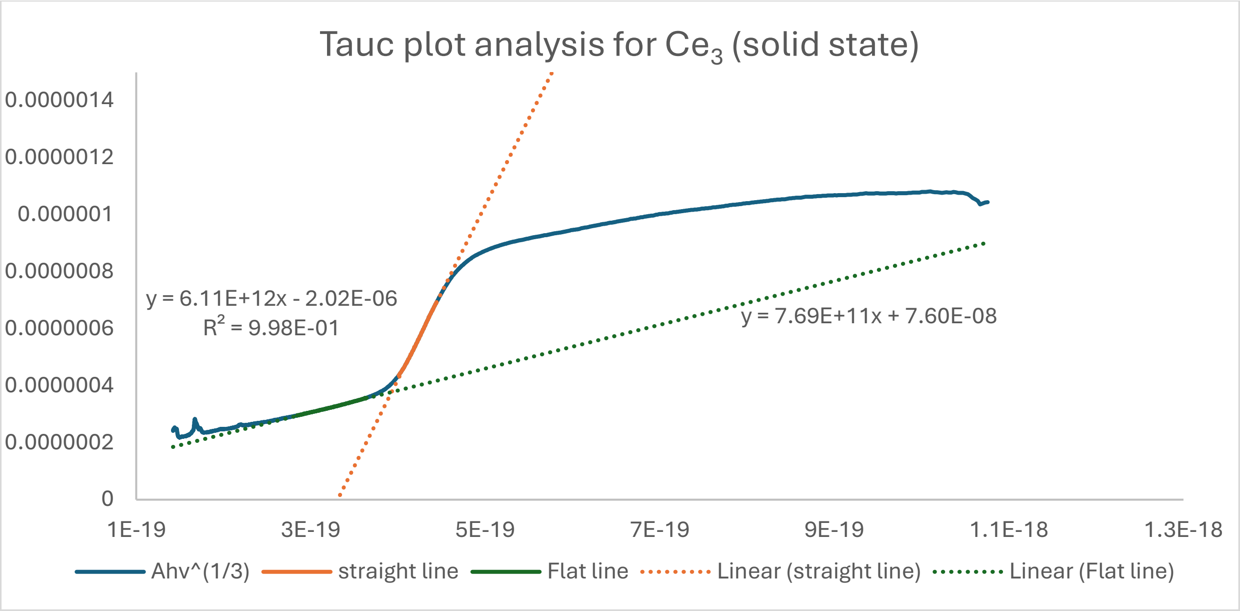
Tauc plot analysis for Ce3 (solid state)
0.0000014
0.0000012
0.000001
0.0000008
0.0000006
0.0000004
0.0000002
0
1E-19
3E-19
5E-19
7E-19
9E-19
1.1E-18
1.3E-18
y = 6.11E+12x - 2.02E-06
R² = 9.98E-01
y = 7.69E+11x + 7.60E-08
Ahv^(1/3)
straight line
Flat line
Linear (straight line)
Linear (Flat line)

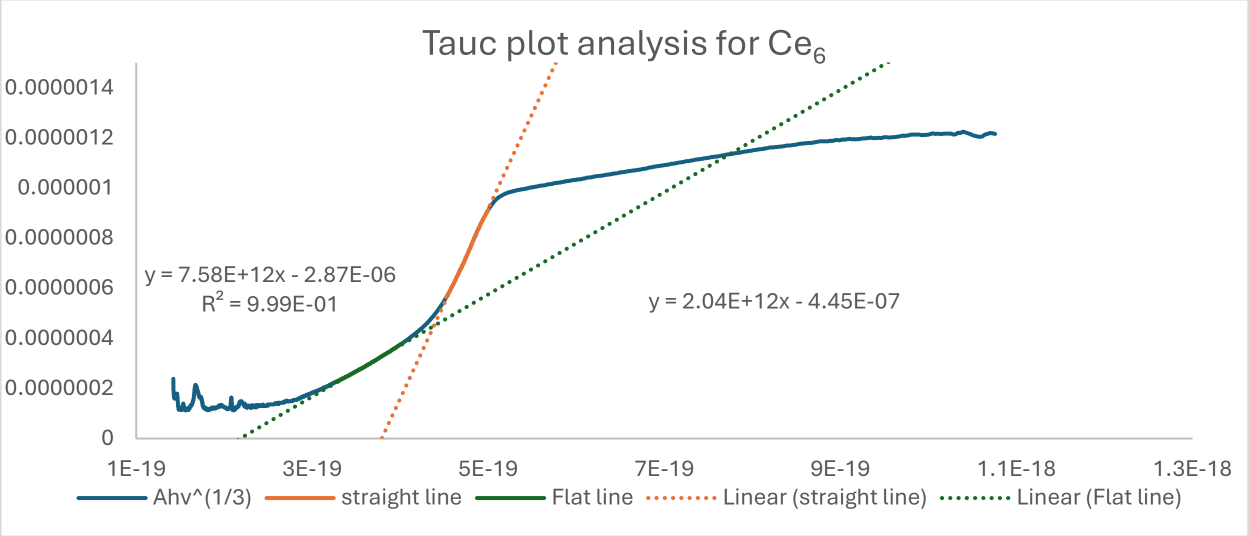
Tauc plot analysis for Ce6
y = 7.58E+12x - 2.87E-06
R2 = 9.99E-01
y = 2.04E+12x - 4.45E-07
0.0000014
0.0000012
0.000001
0.0000008
0.0000006
0.0000004
0.0000002
0
1E-19
3E-19
5E-19
7E-19
9E-19
1.1E-18
1.3E-18
Ahv^(1/3)
straight line
Flat line
Linear (straight line)
Linear (Flat line)

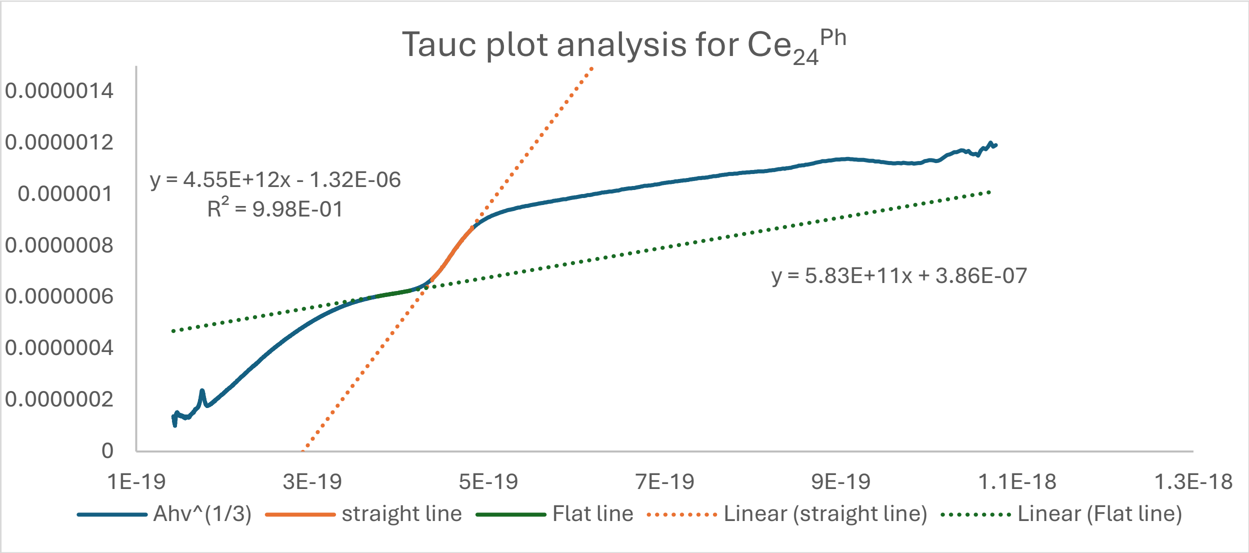
Tauc plot analysis for Ce24Ph
y = 4.55E+12x - 1.32E-06
R2 = 9.98E-01
y = 5.83E+11x + 3.86E-07
0.0000014
0.0000012
0.000001
0.0000008
0.0000006
0.0000004
0.0000002
0
1E-19
3E-19
5E-19
7E-19
9E-19
1.1E-18
1.3E-18
Ahv^(1/3)
straight line
Flat line
Linear (straight line)
Linear (Flat line)

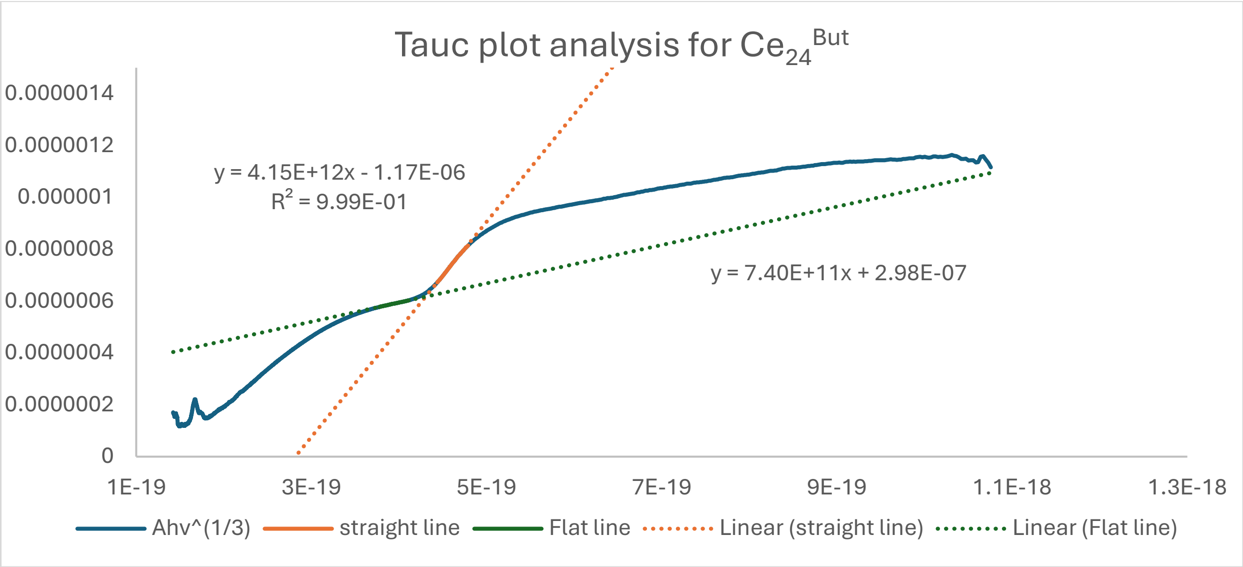
Tauc plot analysis for Ce24But
y = 4.15E+12x - 1.17E-06
R2 = 9.99E-01
y = 7.40E+11x + 2.98E-07
0.0000014
0.0000012
0.000001
0.0000008
0.0000006
0.0000004
0.0000002
0
1E-19
3E-19
5E-19
7E-19
9E-19
1.1E-18
1.3E-18
Ahv^(1/3)
straight line
Flat line
Linear (straight line)
Linear (Flat line)

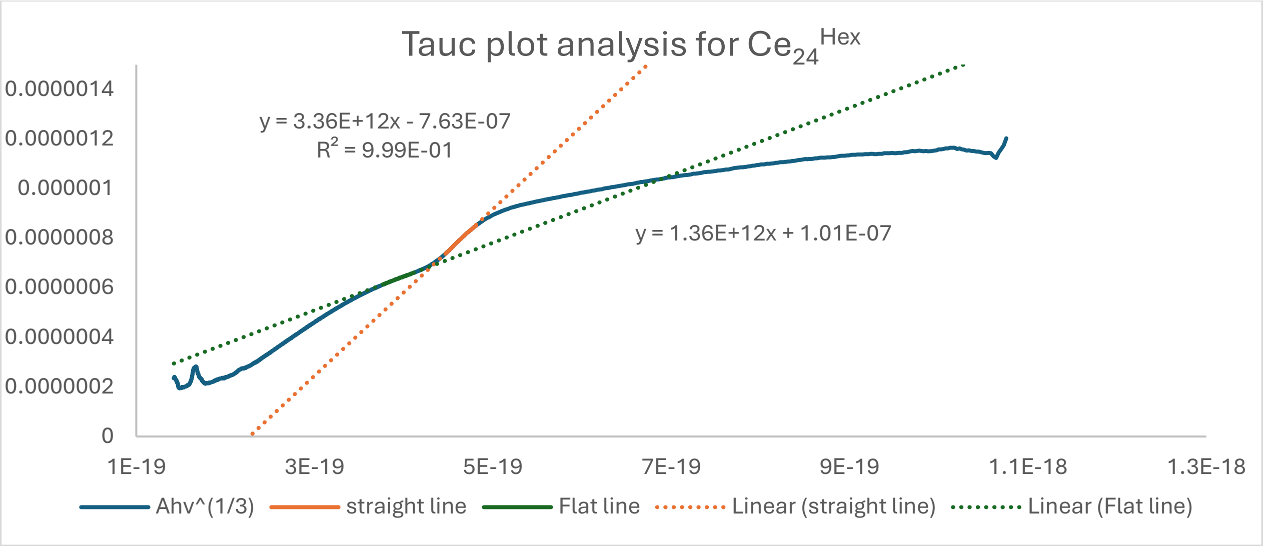
Tauc plot analysis for Ce24Hex
0.0000014
0.0000012
0.000001
0.0000008
0.0000006
0.0000004
0.0000002
0
y = 3.36E+12x - 7.63E-07
R2 = 9.99E-01
y = 1.36E+12x + 1.01E-07
1E-19
3E-19
5E-19
7E-19
9E-19
1.1E-18
1.3E-18
Ahv^(1/3)
straight line
Flat line
Linear (straight line)
Linear (Flat line)

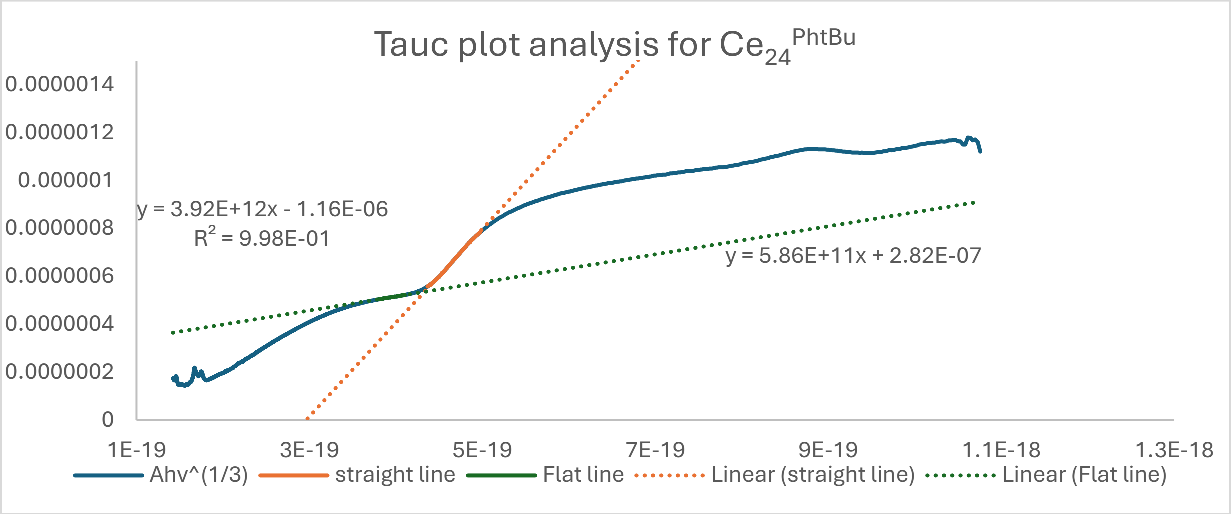
Tauc plot analysis for Ce24PhtBu
0.0000014
0.0000012
0.000001
0.0000008
0.0000006
0.0000004
0.0000002
0
y = 3.92E+12x - 1.16E-06
R2 = 9.98E-01
y = 5.86E+11x + 2.82E-07
1E-19
3E-19
5E-19
7E-19
9E-19
1.1E-18
1.3E-18
Ahv^(1/3)
straight line
Flat line
Linear (straight line)
Linear (Flat line)

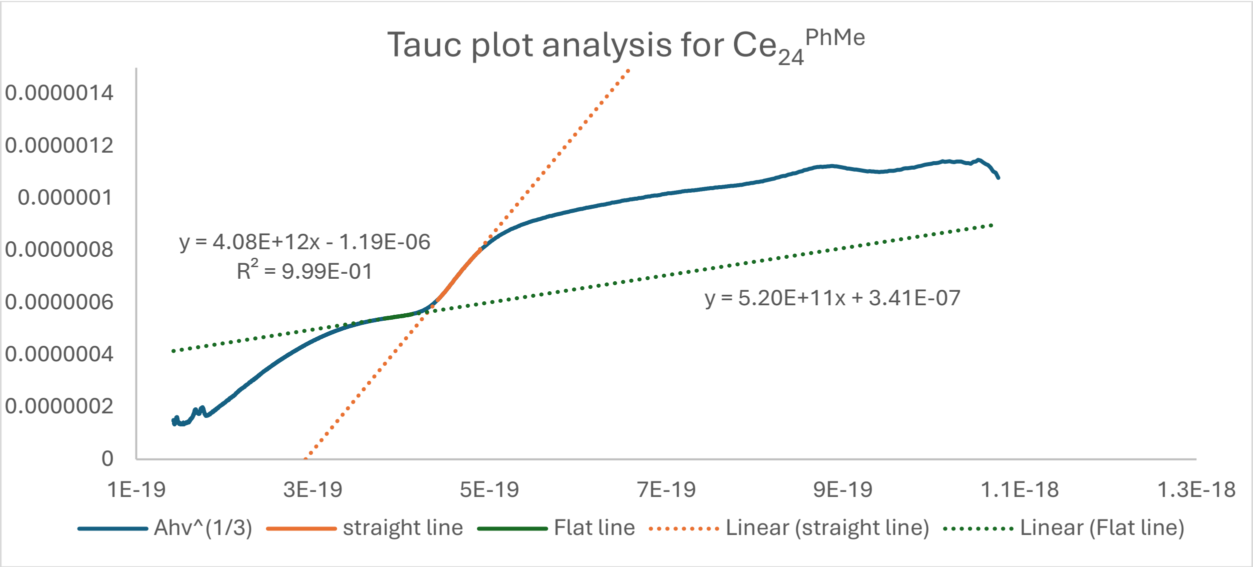
Tauc plot analysis for Ce24PhMe
0.0000014
0.0000012
0.000001
0.0000008
0.0000006
0.0000004
0.0000002
0
y = 4.08E+12x - 1.19E-06
R2 = 9.99E-01
y = 5.20E+11x + 3.41E-07
1E-19
3E-19
5E-19
7E-19
9E-19
1.1E-18
1.3E-18
Ahv^(1/3)
straight line
Flat line
Linear (straight line)
Linear (Flat line)

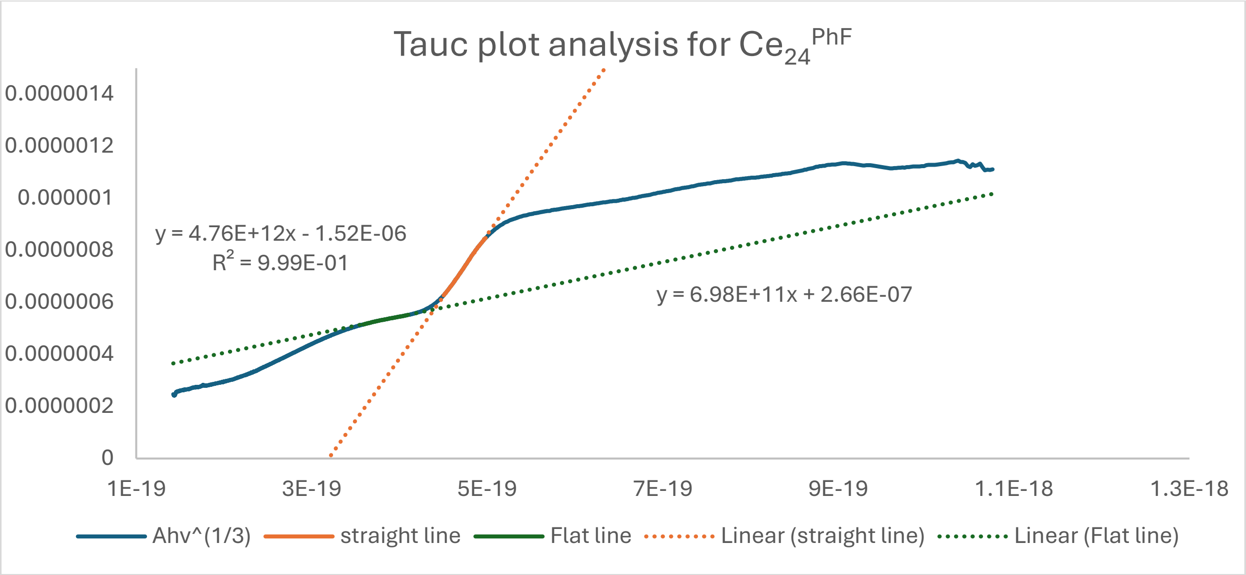

Tauc plot analysis for Ce24PhF
0.0000014
0.0000012
0.000001
0.0000008
0.0000006
0.0000004
0.0000002
0
1E-19
3E-19
5E-19
7E-19
9E-19
1.1E-18
1.3E-18
y = 4.76E+12x - 1.52E-06
R2 = 9.99E-01
y = 6.98E+11x + 2.66E-07
Ahv^(1/3)
straight line
Flat line
Linear (straight line)
Linear (Flat line)

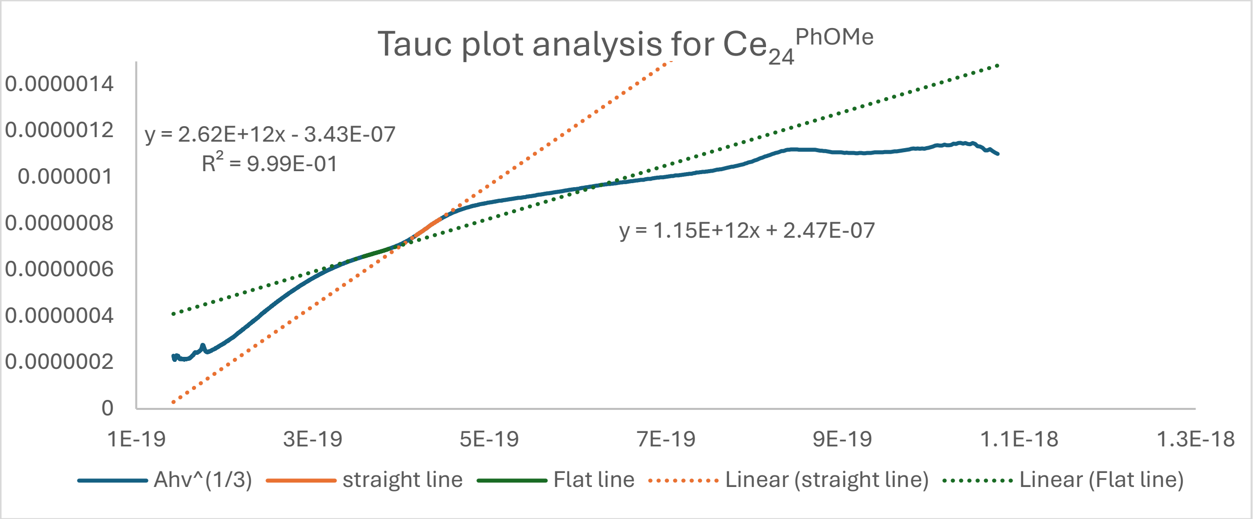

Tauc plot analysis for Ce24PhOMe
0.0000014
0.0000012
0.000001
0.0000008
0.0000006
0.0000004
0.0000002
0
1E-19
3E-19
5E-19
7E-19
9E-19
1.1E-18
1.3E-18
y = 2.62E+12x - 3.43E-07
R2 = 9.99E-01
y = 1.15E+12x + 2.47E-07
Ahv^(1/3)
straight line
Flat line
Linear (straight line)
Linear (Flat line)

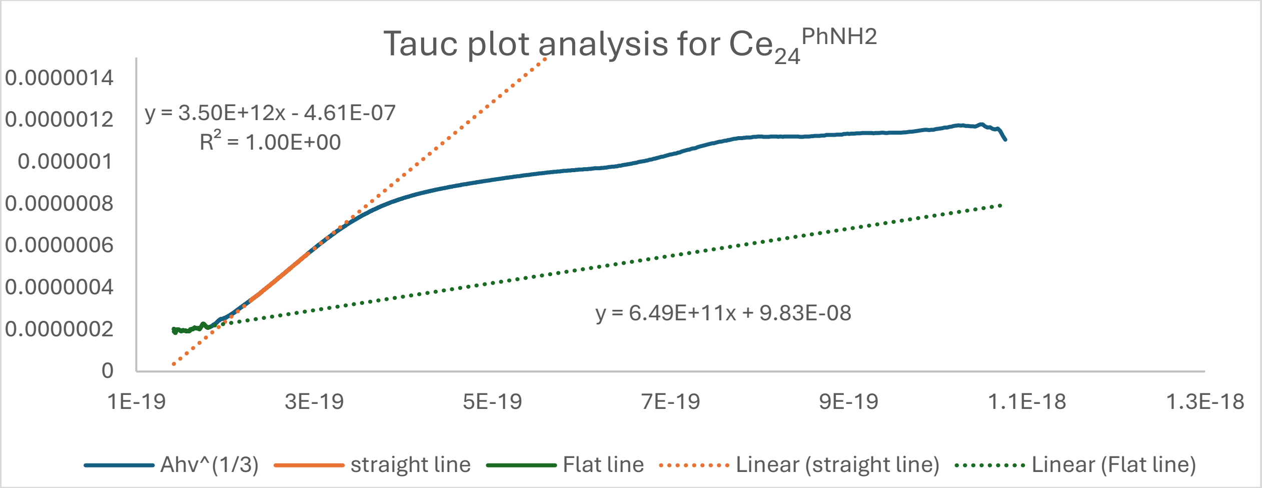

Tauc plot analysis for Ce24PhNH2
0.0000014
0.0000012
0.000001
0.0000008
0.0000006
0.0000004
0.0000002
0
1E-19
3E-19
5E-19
7E-19
9E-19
1.1E-18
1.3E-18
y = 3.50E+12x - 4.61E-07
R2 = 1.00E+00
y = 6.49E+11x + 9.83E-08
Ahv^(1/3)
straight line
Flat line
Linear (straight line)
Linear (Flat line)

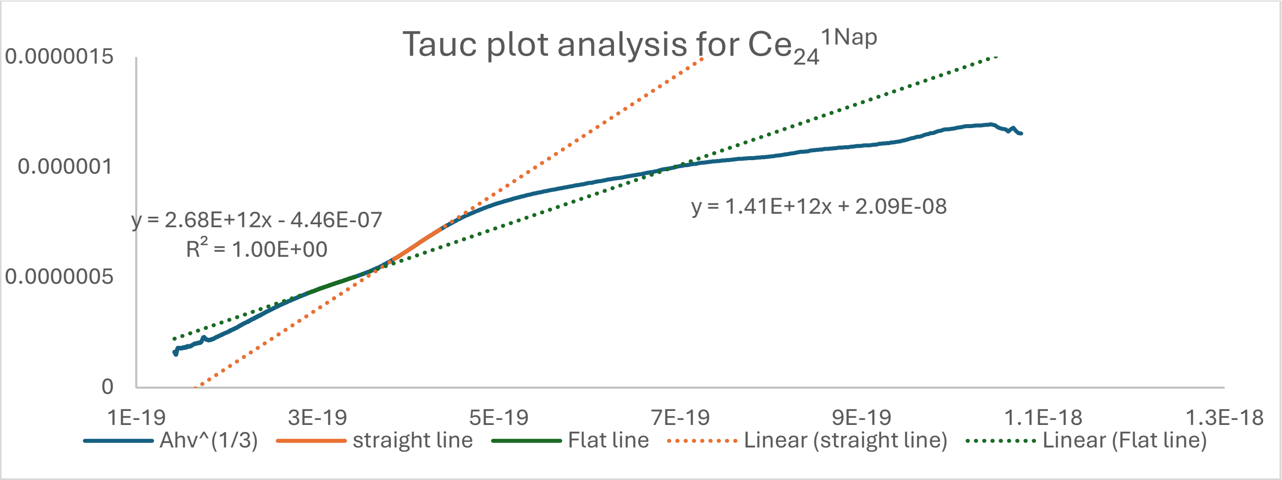
Tauc plot analysis for Ce24 1Nap
0.0000015
0.000001
0.0000005
0
y = 2.68E+12x - 4.46E-07
R2 = 1.00E+00
y = 1.41E+12x + 2.09E-08
1E-19
3E-19
5E-19
7E-19
9E-19
1.1E-18
1.3E-18
Ahv^(1/3)
straight line
Flat line
Linear (straight line)
Linear (Flat line)

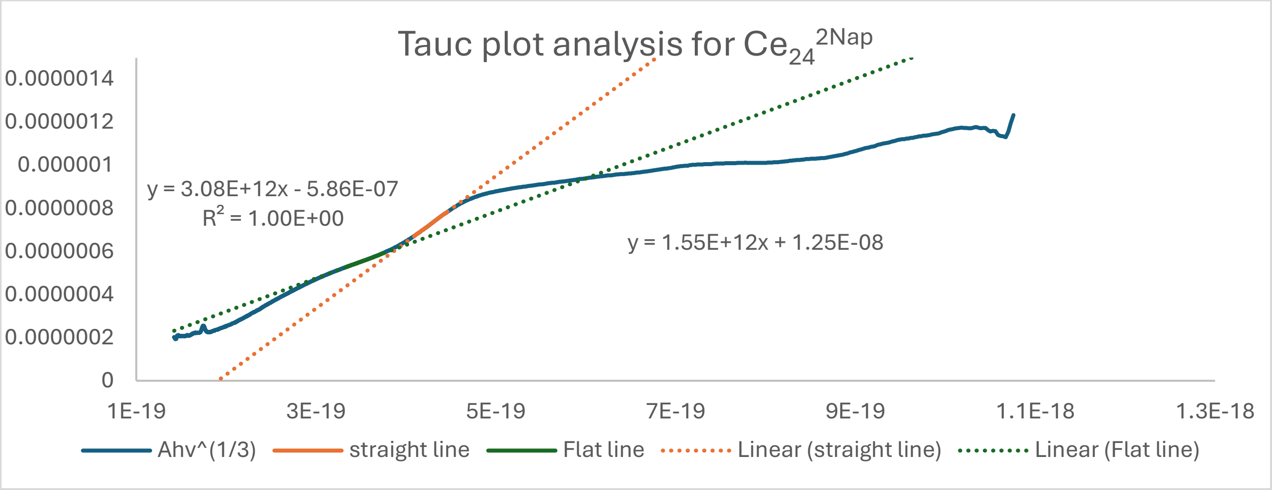
Tauc plot analysis for Ce24 2Nap
0.0000014
0.0000012
0.000001
0.0000008
0.0000006
0.0000004
0.0000002
0
y = 3.08E+12x - 5.86E-07
R2 = 1.00E+00
y = 1.55E+12x + 1.25E-08
1E-19
3E-19
5E-19
7E-19
9E-19
1.1E-18
1.3E-18
Ahv^(1/3)
straight line
Flat line
Linear (straight line)
Linear (Flat line)

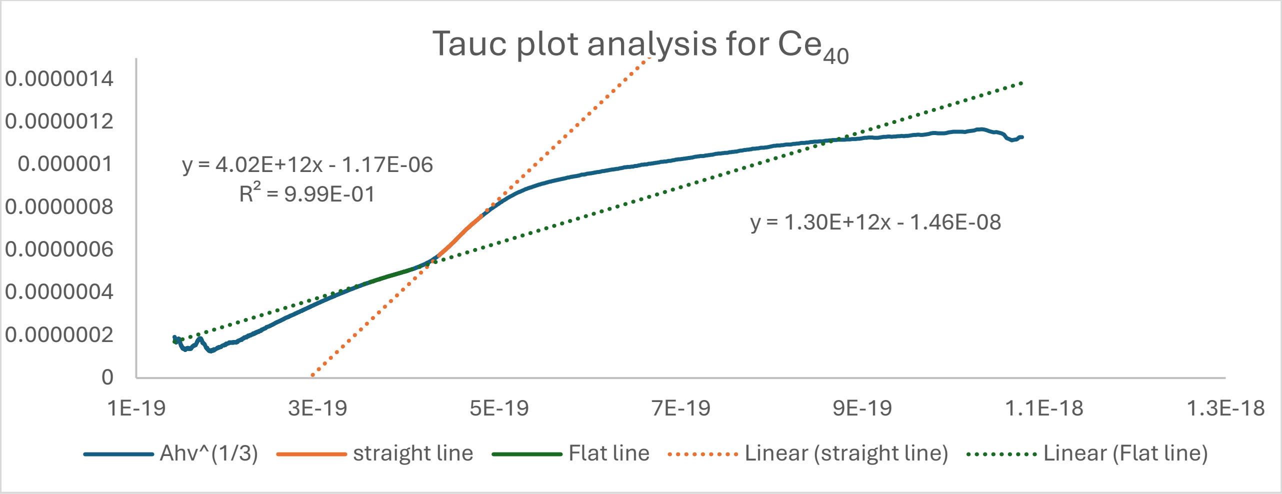
Tauc plot analysis for Ce40
0.0000014
0.0000012
0.000001
0.0000008
0.0000006
0.0000004
0.0000002
0
y = 4.02E+12x - 1.17E-06
R2 = 9.99E-01
y = 1.30E+12x - 1.46E-08
1E-19
3E-19
5E-19
7E-19
9E-19
1.1E-18
1.3E-18
Ahv^(1/3)
straight line
Flat line
Linear (straight line)
Linear (Flat line)

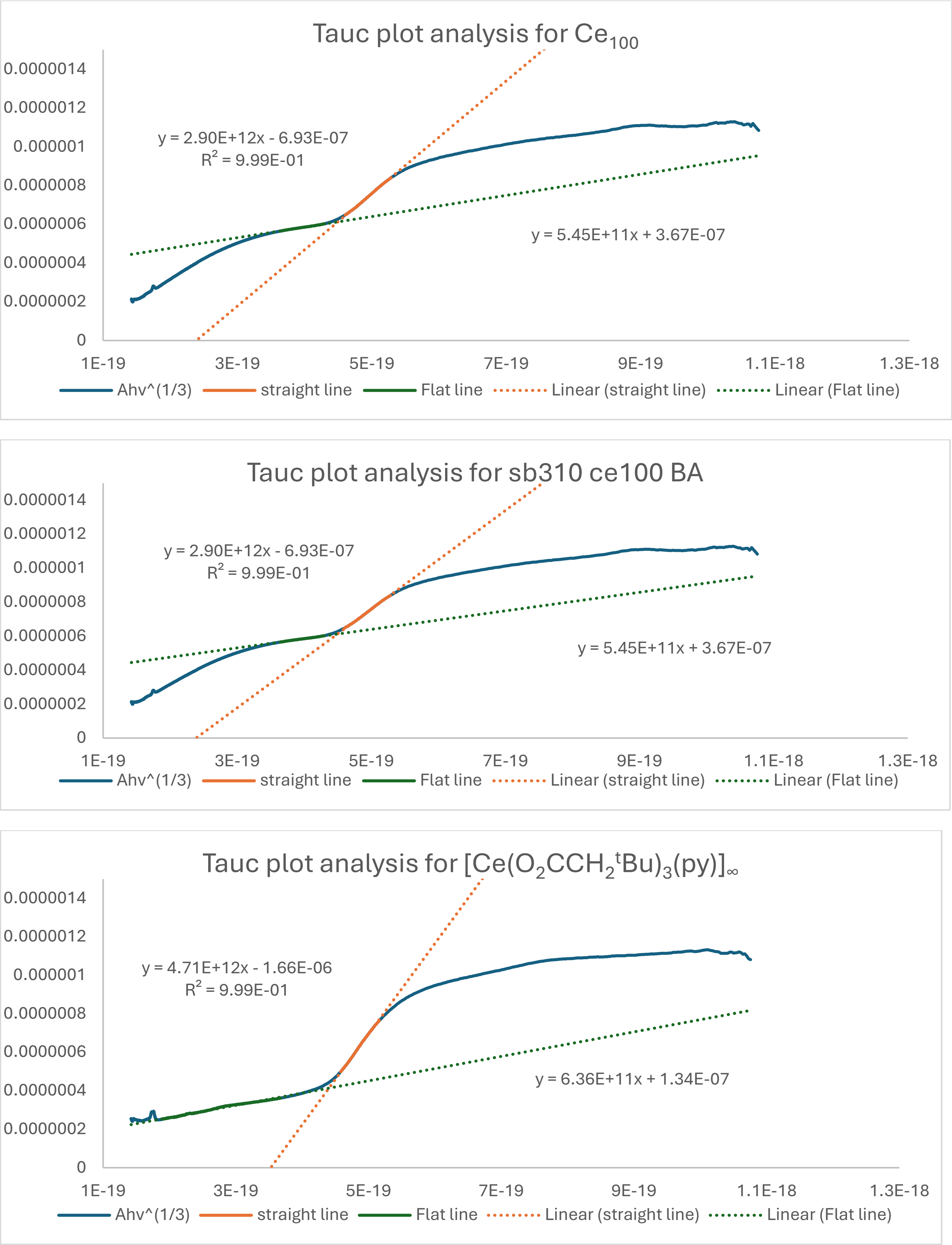

Figure S23. Tauc's plots of all Ce-oxo clusters studied (using n = 3, forbidden indirect transition, noting the O 2p to Ce 4f is a Laporte forbidden transition)

Table S10. Table of absorption onset values derived from Tauc's plot analysis. *Note the onset value for Ceria Merck 544841 (<25 nm) is difficult to fit, due to a rising baseline beyond 600 nm, and a smoothly dropping absorption curve, which generates an onset value much lower that expected using this analysis (compared to similar spectra of other cerias), this highlights the importance of comparative visual inspection of the spectra rather than purely mathematical fitting of an onset value.

Sample	Forbidden Indirect (n = 3) absorption onset (diffuse reflectance) eV (nm)	Forbidden Indirect (n = 3) absorption onset (solution) eV (nm)
Ceria NIST (SRM 674b)	3.09 eV (401 nm)	
Ceria Merck 22390	2.78 eV (447 nm)	
Ceria Merck 544841	*2.19 eV (567 nm)**	
Ceria Solvay (HSA 5)	2.61 eV (476 nm)	
Ceria Strem Puratrem	2.63 eV (472 nm)	
Ce_3	2.45 eV (507 nm)	2.73 eV (455 nm) toluene
Ce_6	2.73 eV (454 nm)	
Ce_{24}^{Ph}	2.68 eV (463 nm)	
Ce_{24}^{But}	2.69 eV (462 nm)	
Ce_{24}^{Hex}	2.70 eV (460 nm)	
Ce_{24}^{PhtBu}	2.70 eV (460 nm)	2.69 eV (461 nm) THF<br>2.73 eV (454 nm) toluene
Ce_{24}^{PhMe}	2.69 eV (463 nm)	
Ce_{24}^{PhF}	2.74 eV (453 nm)	
Ce_{24}^{PhOMe}	2.50 eV (496 nm)	
Ce_{24}^{PhNH2}	1.23 eV (1013 nm)	
Ce_{24}^{1Nap}	2.31 eV (538 nm)	
Ce_{24}^{2Nap}	2.44 eV (508 nm)	
Ce_{40}	2.65 eV (469 nm)	
Ce_{100}	2.81 eV (443 nm)	
Ce-UIO-66	2.85 eV (436 nm)	
$[Ce(O_2CCH_2{}^tBu)_3(py)]_\infty$	2.75 eV (452 nm)	
$Ce(acetate)_3$ hydrate	3.00 eV (414 nm)	

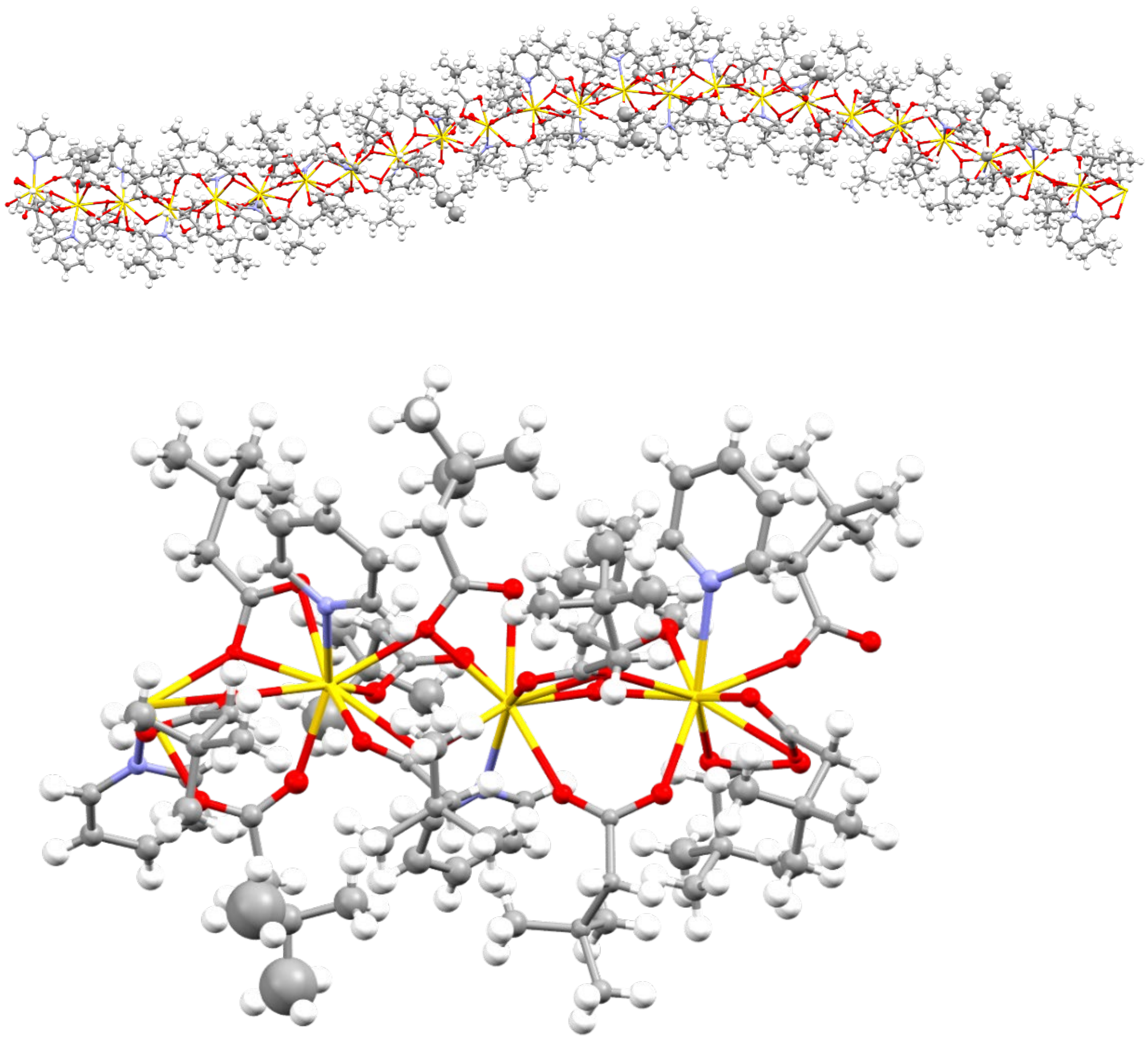

Figure S24. SCXRD structure of [Ce(OOCCH$_2$^{t}Bu)$_3$(py)]$_\infty$, top; one complete helix, bottom; asymmetric unit. Displacement spheres drawn at 50%; cerium (gold), oxygen (red), carbon (grey), nitrogen (blue) and hydrogen (white).

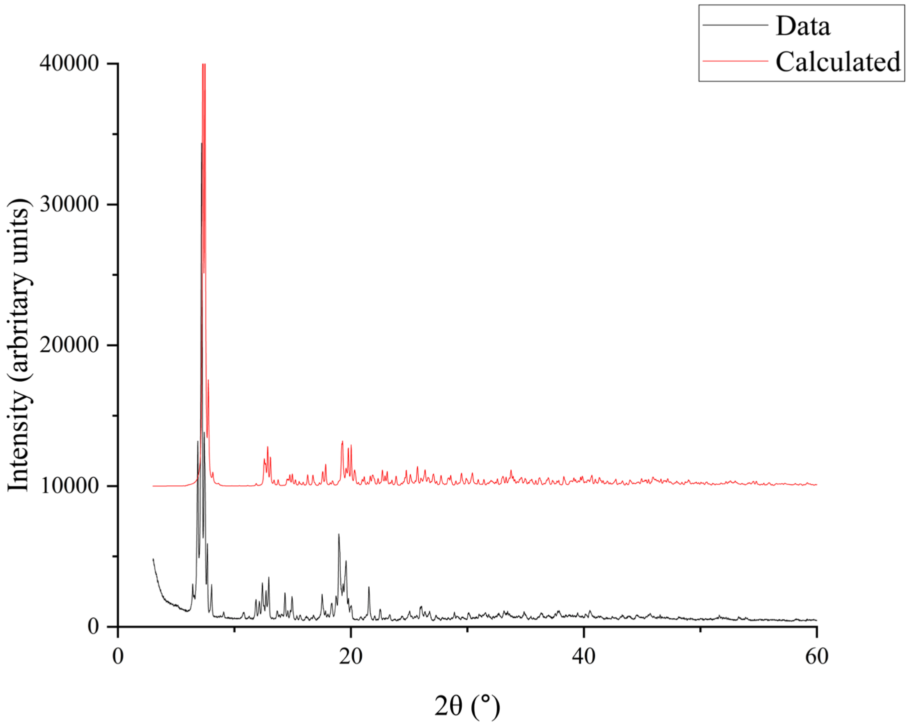

Figure S25 – PXRD pattern of $[Ce(O_2CCH_2{}^tBu)_3(py)]_\infty$ at room temperature compared to calculated pattern derived from the 100 K SCXRD structure.

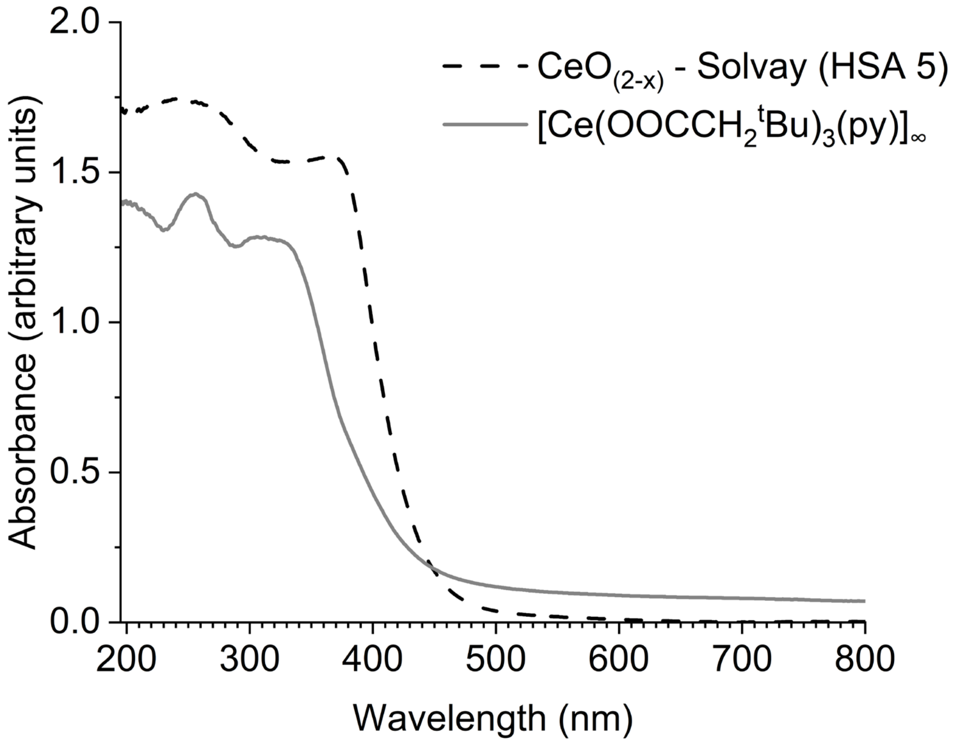

Figure S26. Diffuse reflectance UV/vis spectra of $[Ce(O_2CCH_2{}^tBu)_3Py]_\infty$, with only Ce(III), compared to high surface area ceria.

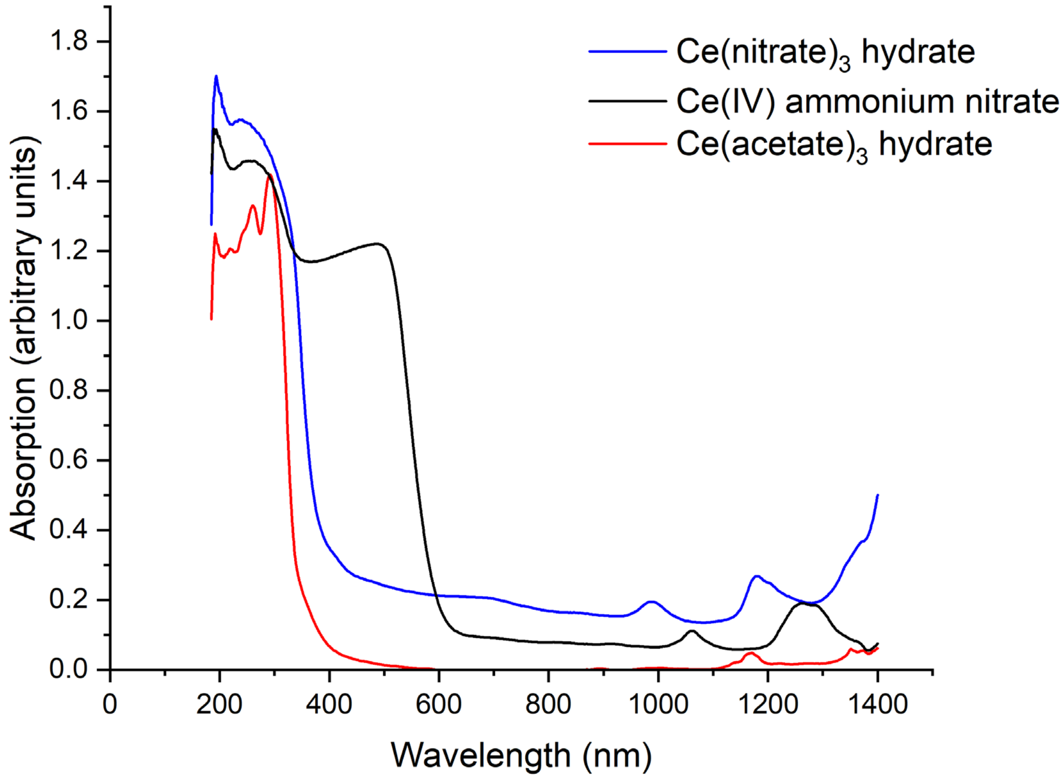

Figure S27. Diffuse reflectance UV/vis spectra of Ce nitrate starting materials and Ce(III) acetate hydrate.

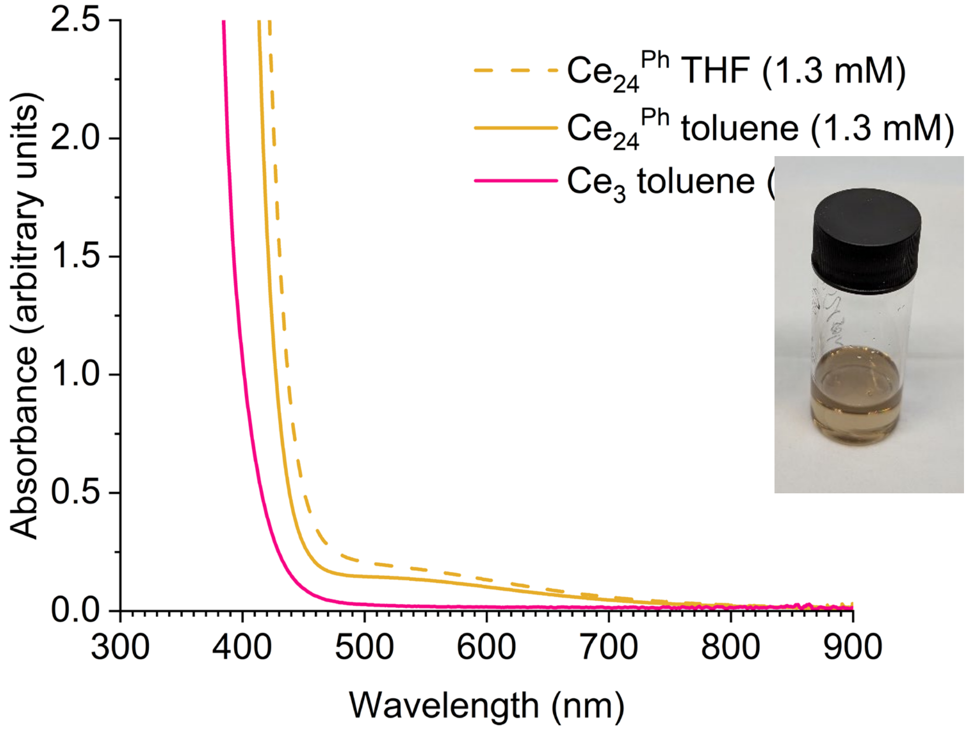

Figure S28. Solution UV-vis spectrum of **Ce_3** and **Ce_{24}^{PhtBu}** (with a photograph showing the colour of the solution of Ce_{24}^{PhtBu})

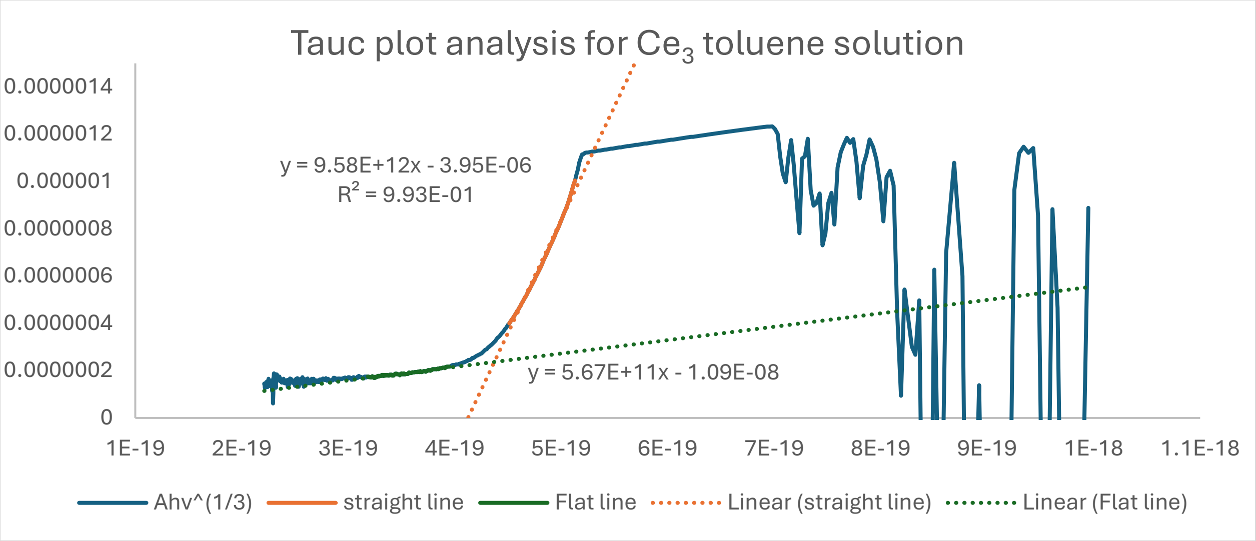

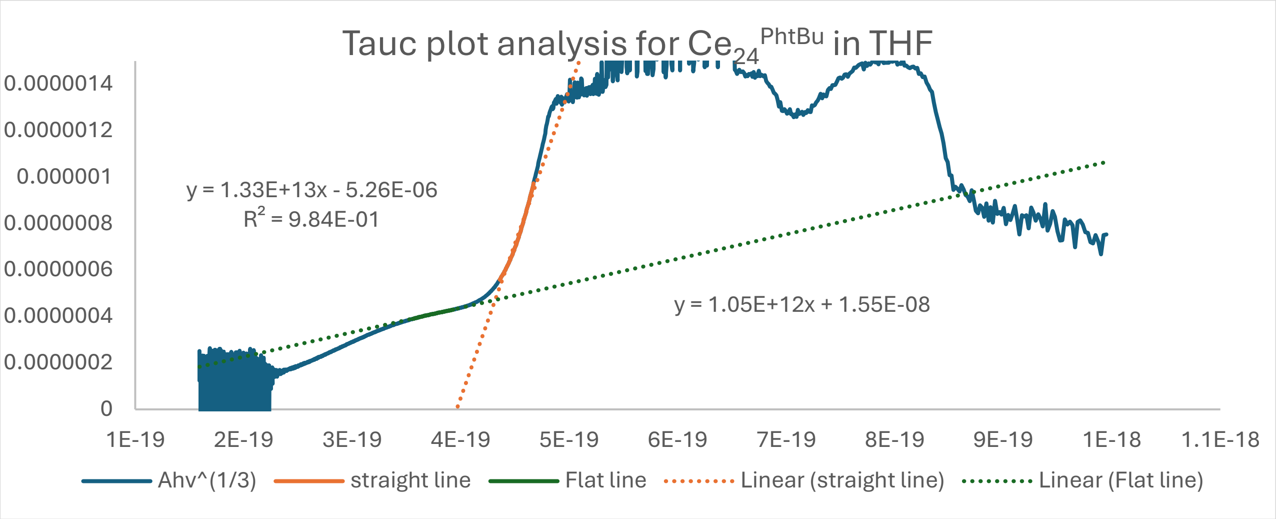

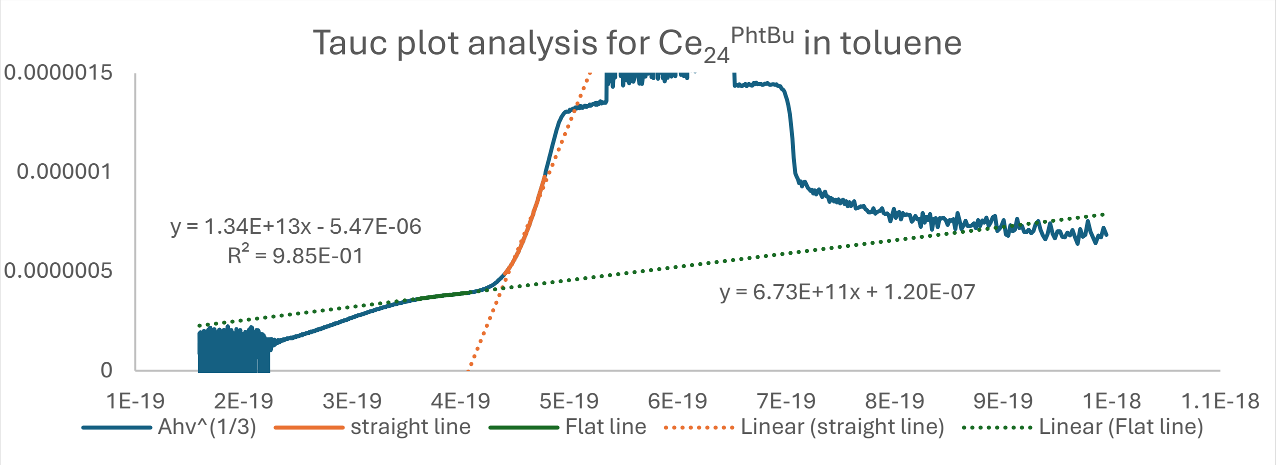

Figure S29. Tauc's plot analysis of solution spectra

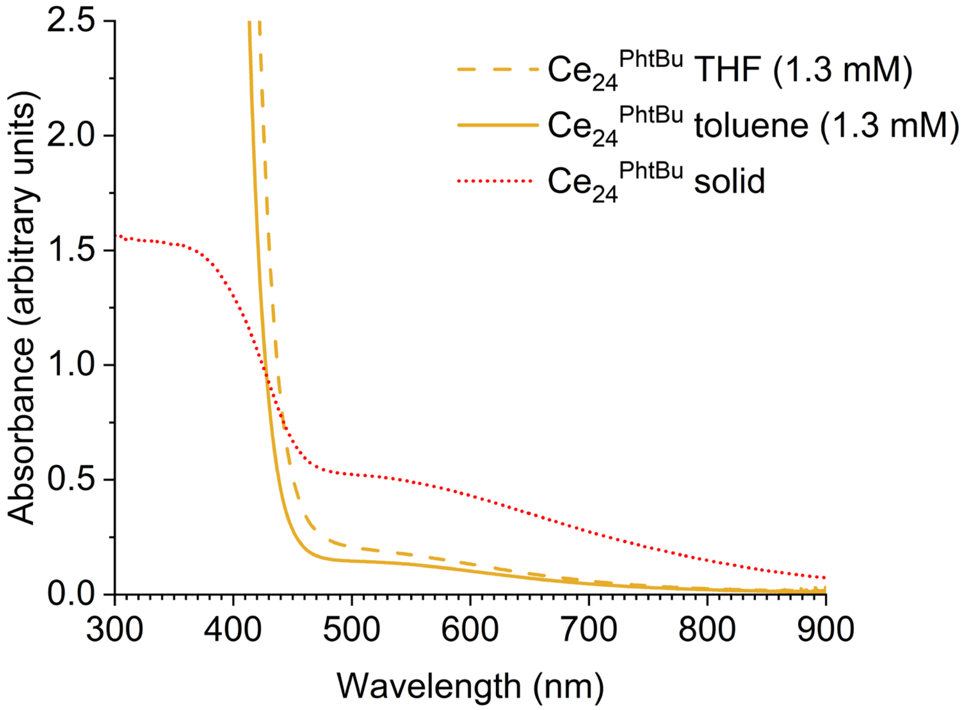

Figure S30. Solution and diffuse reflectance UV-vis spectrum of $\mathbf{Ce_{24}^{PhtBu}}$ for solution and solid-state samples respectively.

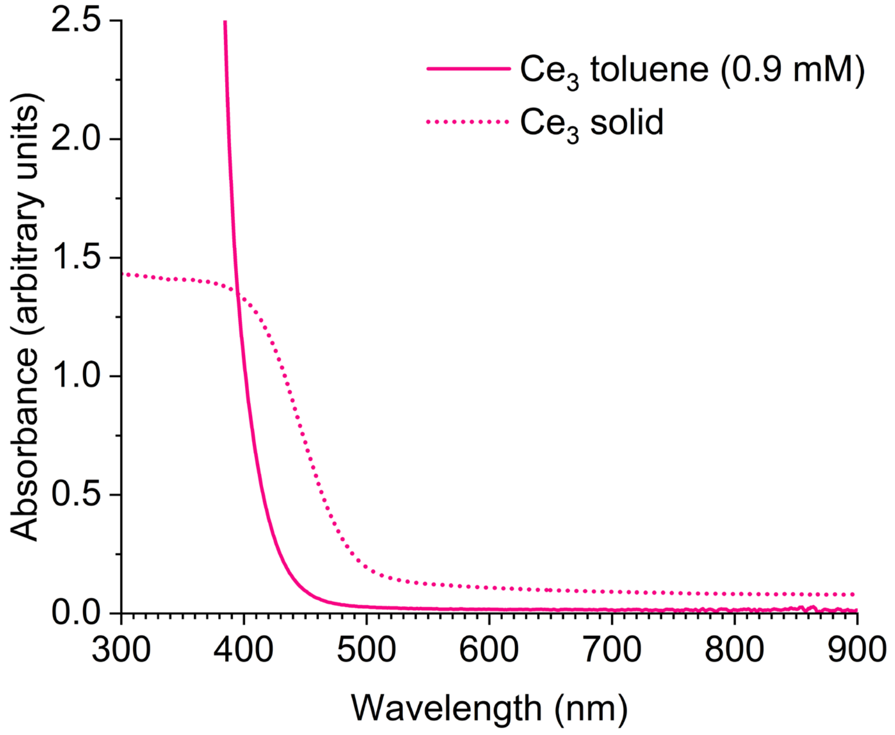

Figure S31. Solution and diffuse reflectance UV-vis spectrum of $\mathbf{Ce_3}$ for solution and solid-state samples respectively.

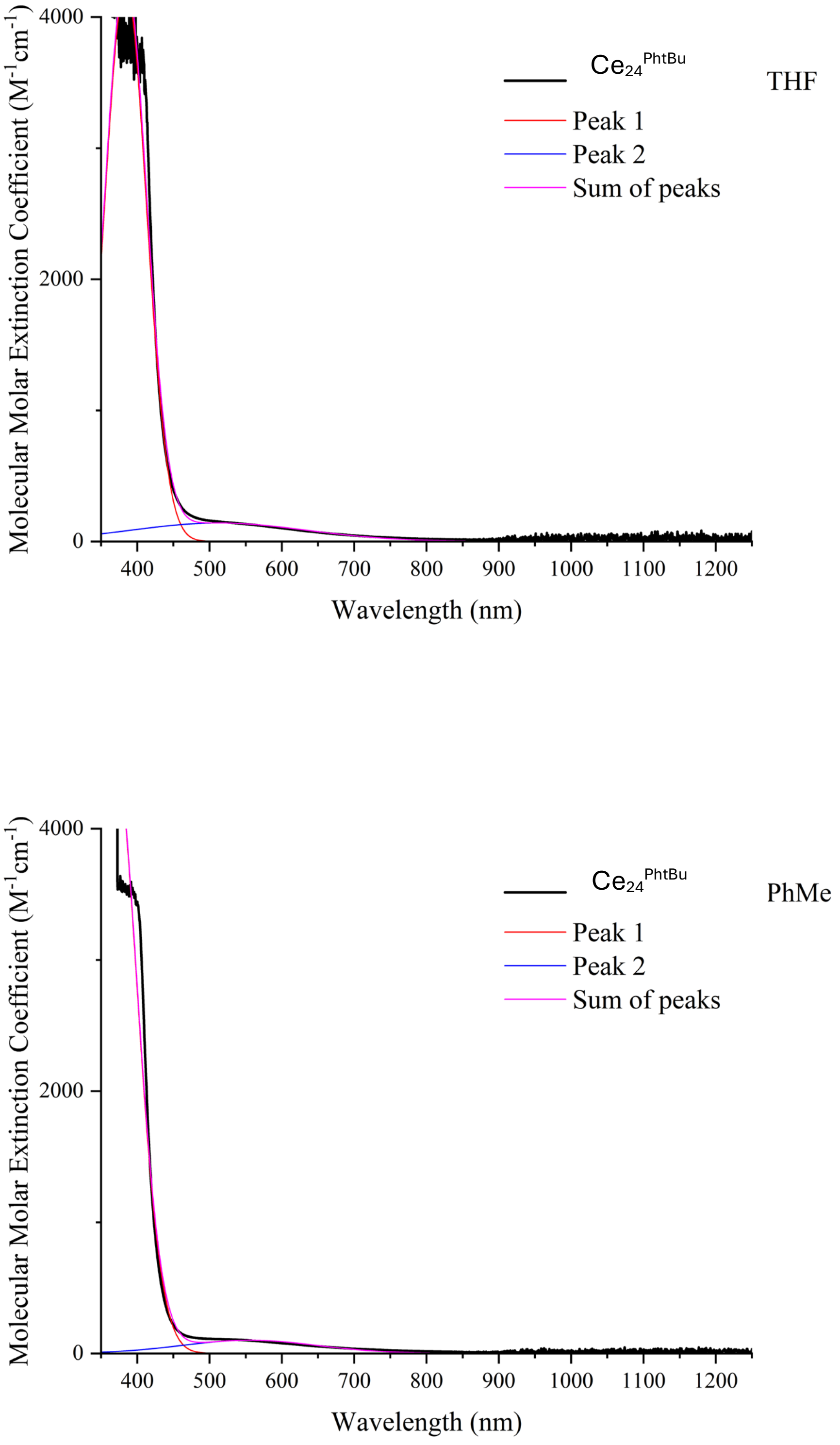

Figure S32. Gaussian peak fitting of solution UV/visible spectra of **Ce_{24}^{PhtB}** (top in THF, bottom in toluene).

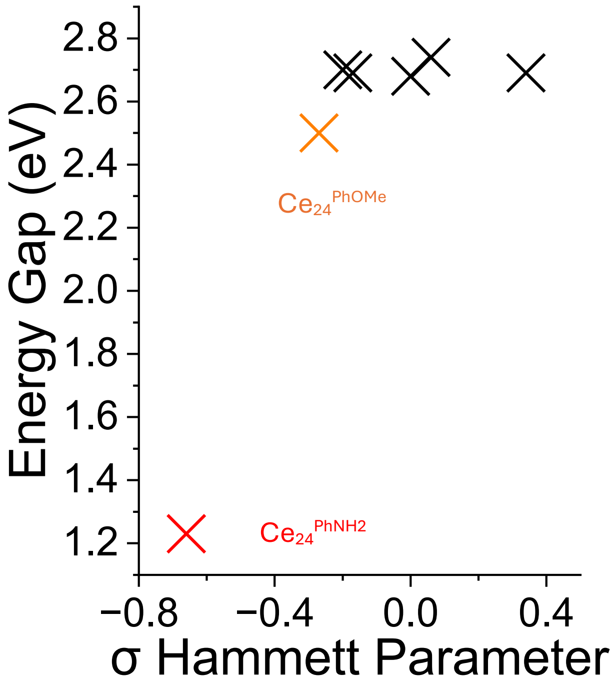

Figure S33. Energy gaps of Ce-oxo clusters versus Hammett parameter of ligands.

Supporting Note 2. Near-IR signals

The other notable features in the UV-visible-NIR spectra of Ce-oxo clusters are the NIR peaks between 1100 and 1400 nm. These are not electronic transitions, instead, they are vibrational overtones. There are three regions of interest; one at ~1140-1150 nm, another at ~1190 nm and a further feature >1350 nm that extends past 1400 nm, above the NIR detector range on the spectrometer used.

The peak at ~1140 nm is attributed to the 2nd overtone aromatic C-H stretch and is present in compounds that have aromatic C–H bonds in the carboxylate ligands such as $\mathbf{Ce_{24}^{Ph}}$, and $\mathbf{Ce_{100}}$.[61] Additionally, compounds containing pyridine also show stretches around this frequency. At ~1150 nm there is a second peak or shoulder in $\mathbf{Ce_3}$, $\mathbf{Ce_6}$, $\mathbf{Ce_{24}^{PhtBu}}$, $\mathbf{Ce_{24}^{But}}$ and $\mathbf{Ce_{24}^{PhMe}}$, $\mathbf{Ce_{40}}$ attributable to the 2nd overtone asymmetric CH_3 stretches.

At 1190 nm there is a stretch observed which is attributable to the 2nd overtone C–H stretches including CH_3 and CH_2 and is not observed in compounds without alkyl moieties including $\mathbf{Ce_{24}^{Ph}}$.[61]

There are stretches from 1350 nm onwards that are associated with the first overtone of O–H stretches and the 1st overtone of combination C–H stretches.[61] These features can be attributed to a complex mixture of stretches combined with features above the detector limit of 1400 nm and are therefore not assigned.

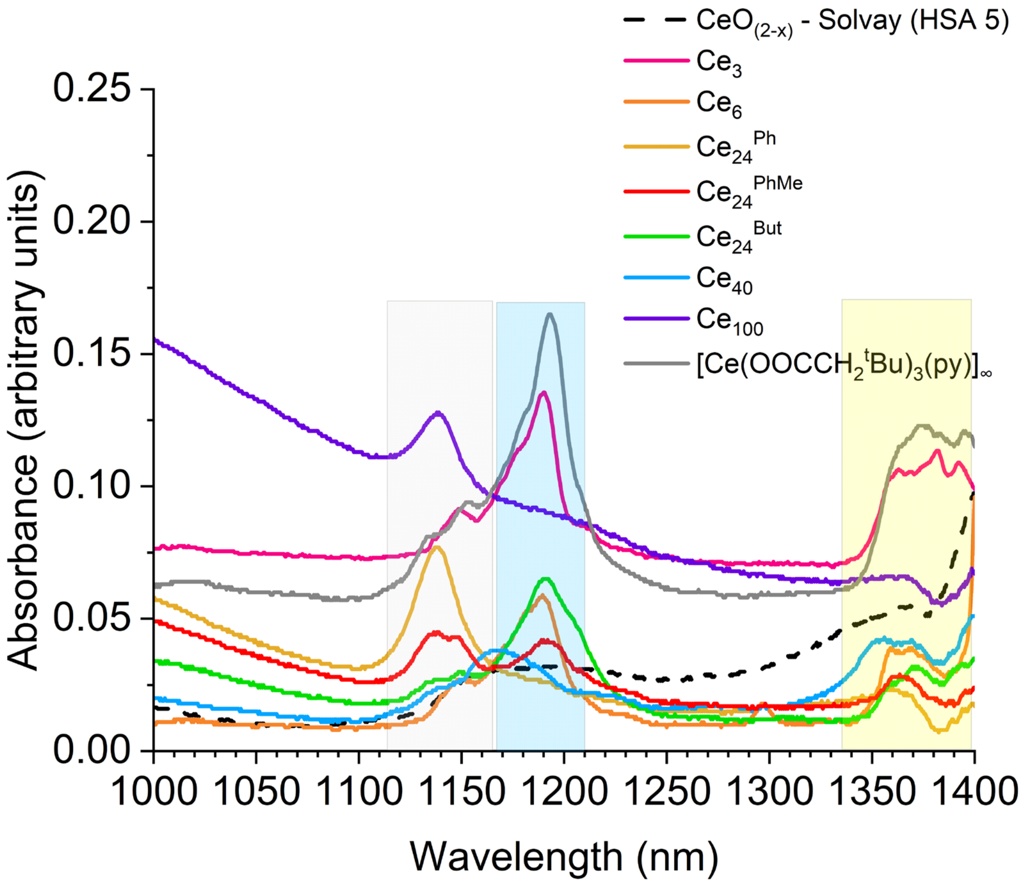

Figure S34 – NIR spectra of cerium compounds showing the region associated with 2nd overtone stretches. Grey shaded region, aromatic C-H and CH_3 2nd overtone stretches; blue region, aliphatic C-H 2nd overtone stretch; yellow region, O-H and C-H first overtones.

Supporting note 3. Preparation of **$Ce_{22}La_2^{Ph}$**.

The reaction to **Ce_{24}^{Ph}** was repeated using La(III)$(NO_3)_3(H_2O)_6$ instead of Ce(III)$(NO_3)_3(H_2O)_6$. The reaction proceeded as expected, but the solution remained yellow (and did not darken to brown). Yellow crystals were studied by single crystal X-ray diffraction and revealed the expected unit cell and core structure for **Ce_{24}^{Ph}** and bulk powder diffraction was also consistent (Fig S11). Elemental analysis was consistent with the expected bulk structure. CHN analysis: calculated for $C_{230}H_{174}Ce_{24}N_4O_{96}$: C, 35.00 %, H 2.22 % N 0.71%. Found C 35.4 %, H 2.15 %, N 0.95 %. ICP-OES analysis revealed La was incorporated into the product, Ce wt %, found 36.0%, calc. 37.91; La wt%, found 4.97%, calc, 3.42%. The slightly higher than expected La content, may imply that more than two sites of the Ce_{24} structure can be occupied by M(III) cations.

Solid-state UV-vis spectroscopy shows a similar spectrum to **Ce_{24}^{Ph}** but with a significantly weaker IVCT signal (Fig S32). This is consistent with partial replacement of the surface Ce(III) sites by La(III), but also suggests some redox reactions during the synthesis also incorporate some Ce(III) into these sites. It is noteworthy that the intensity of diffuse reflectance UV/vis spectra are challenging to compare due to differences in sample packing, and that other Ce_{24} clusters (with larger volume of surface ligands, so less CeO_2 per unit volume) also have weaker IVCT bands.

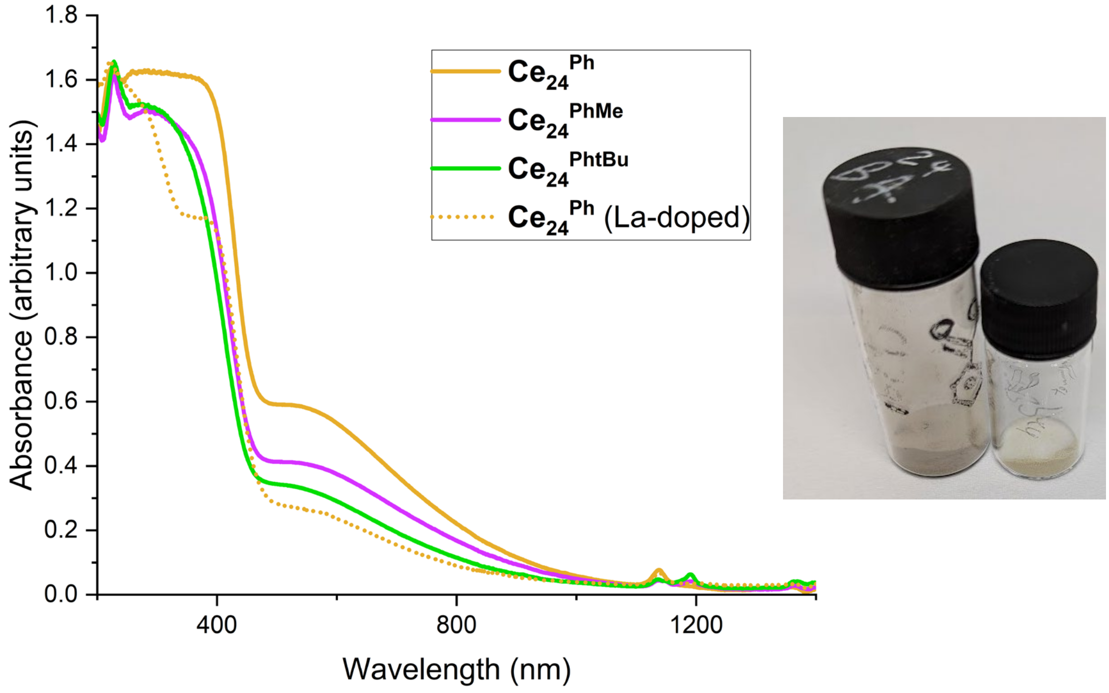

Figure S35. Diffuse reflectance UV/vis spectra of a lanthanum doped Ce_{24} cluster, compared to all Ce structures. Photograph of powders of $\mathbf{Ce_{24}^{Ph}}$ (left) and La doped $\mathbf{Ce_{24}^{Ph}}$ (right).

Crystallography Details

In general, the large molecule size and large solvent volume and fragility of the crystals made high quality data collection to the standard typically expected for small molecule crystallography difficult, analogous to the well-studied issues in protein crystallography.

Comments on specific structures

$\mathbf{Ce_{24}^{Ph}}$**:** Full structure was solved for the cerium-oxo cluster molecule and most of the pyridine solvent found. Similarity restraints were used for some solvent pyridine molecules and one benzoate ligand. An additional 8 pyridines are in the unit cell, implied from PLATON SQUEEZE.

$\mathbf{Ce_{24}^{Bu}}$**:** It was only possible to solve the cerium-oxo core. This is presented to report the unit cell and to confirm that a Ce_{24} nuclearity oxo cluster is the crystalline product.

$\mathbf{Ce_{24}^{Hex}}$**:** It was only possible to solve the cerium-oxo core. This is presented to report unit cell and to confirm that a Ce_{24} nuclearity oxo cluster is the crystalline product.

$\mathbf{Ce_{24}^{PhMe}}$**:** The full structure was solved for the cerium-oxo cluster molecule and most of the pyridine solvent molecules found. Similarity restraints were used for some solvent pyridine molecules. An additional 5.5 pyridines are in the unit cell, implied from PLATON SQUEEZE.

$\mathbf{Ce_{24}^{PhtBu}}$**:** The full structure was solved for the cerium-oxo cluster molecule and most of the pyridine solvent molecules found. Similarity restraints were used for some solvent pyridine

molecules and some para-tert-butylbenzoate ligands. Approximately 81 additional solvent pyridines are in the unit cell, implied from PLATON SQUEEZE.

Ce_{24}^{PhF}: The full structure was solved for the cerium-oxo cluster molecule and some of the pyridine solvent molecules found. Similarity restraints were used for some solvent pyridine molecules and some para-fluorobenzoate ligands. An additional 31 pyridines are in the unit cell, implied from PLATON SQUEEZE.

Ce_{24}^{PhOMe}: The full structure and connectivity was solved for the cerium-oxo cluster molecule including all 30 anisate ligands, and some of the pyridine solvent molecules found. Similarity restraints were used extensively for some solvent pyridine molecules. An additional 44 pyridines are in the unit cell, implied from PLATON SQUEEZE.

Ce_{24}^{PhNH2}: It was only possible to solve the cerium-oxo core and some para-aminonenzoate ligands. This is presented to report the unit cell and to confirm that a Ce_{24} nuclearity oxo cluster is the crystalline product.

Ce_{24}^{1Nap}: It was only possible to solve the cerium-oxo core and carboxylate groups, the napthyl groups could not be full resolved. This is presented to report the unit cell and to confirm that a Ce_{24} nuclearity oxo cluster is the crystalline product with 30 carboxylate ligands.

Ce_{24}^{2Nap}: The crystal cracked below 150K and so was collected at 200K. The full structure was solved for the cerium-oxo cluster molecule and some of the pyridine solvent molecules found. Similarity restraints were used extensively for napthoate ligands and solvent pyridine molecules. An additional 38 pyridines are in the unit cell, implied from PLATON SQUEEZE.

$[Ce_{24}O_{28}(OH)_8(O_2C\text{-meta-}C_6H_4F)_{30}(py)_4](py)_{22}$: The full structure was solved for the cerium-oxo cluster molecule and all of the pyridine solvent found. Meta-fluorobenzoate ligands were positionally disordered and the occupancy was refined independently for each ligand. Where the occupancy of F was close to zero the second part was removed and no disorder was modelled. Similarity restraints were not used for fluorobenzoate ligands but thermal similarity was used especially for low occupancy fluorines. Similarity restraints were used for some solvent pyridines. Additionally, PLATON SQUEEZE found no additional solvent voids and the output was not applied to the structure factors.

$[Ce(O_2CCH_2{}^tBu)_3(py)]_\infty$: The Full structure was solved with isotropic thermal parameters. A very large cell axis along C with diffuse scattering in this direction also meant data quality in this direction was relatively poor and limited anisotropic refinement. This was accompanied by very anisotropic needle crystals (10 mm x 100 µm x 100 µm). Crystals were cleaved for collection. Most crystals were racemic; however, one crystal (or cleaved domain of crystal) was found that was the single enantiomorph and this is presented.

Crystallography table

Table S11. Crystallographic data.

Compound	Ce_{24}^{Ph}	Ce_{24}^{Bu}	Ce_{24}^{hex}
**CCDC number**	2418678	N/A	N/A
**Formula**	$[Ce_{24}O_{28}(OH)_8(OOCPh)_{30}(Py)_4](Py)_{14}$	$[Ce_{24}O_{28}(OH)_8(OOCPr)_{30}(Py)_4](Py)_x$	$[Ce_{24}O_{28}(OH)_8(OOC(CH_2)_4CH_3)_{30}(Py)_4](Py)_x$
**X-ray source**	Mo Kα	Mo Kα	Synchroton
**wavelength [Å]**	0.71073	0.71073	0. 68890
**Empirical Formula**	$C_{300}H_{230}Ce_{24}N_{18}O_{96}$	$C_{140}H_{234}Ce_{24}N_4O_{96}$	$C_{200}H_{354}Ce_{24}N_4O_{96}$
**M [gmol^{-1}]**	8985.95	6872.13	7713.75
**Crystal System**	monoclinic	orthorhombic	triclinic
**Space Group**	$P\,2_1/n$	$P\,m\,2_1\,n$	P -1
**Size [mm]**	0.076 × 0.168 × 0.324	0.067 × 0.118 × 0.568	0.1 × 0.1× 0.1
**Colour / Shape**	yellow plate	brown prism	brown block
**T [K]**	100.0(1)	100.0(1)	100.0(1)
**a [Å]**	21.834400(17)	27.1952(8)	20.47780(4)
**b [Å]**	26.493099(17)	20.7368(6)	20.87490(3)
**c [Å]**	25.106501(14)	19.9816(6)	20.91000(3)
**α [deg]**	90	90	68.359(4)
**β [deg]**	90.7010(18)	90	85.765(5)
**γ [deg]**	90	90	61.627(4)
**V [Å^{-3}]**	14522.043(18)	11268.5(6)	7253.9(4)
**Z**	2	2	1
**θ range [deg]**	2.166 – 30.582	3.406 – 30.882	1.023– 25.151
**Reflections**	327519	72061	63964
**R int**	0.051	0.057	0.144
**data/restr/par**	38438/584/1919	24607/1/313	20304/0/153
**R1 [I>2σ(I)]**	0.0527	0.1538	0.2639
**wR2 [all data]**	0.1363	0.4847	0.4843
**GoF**	0.9989	4.001	0.9443
**Largest diff. peak and hole [eÅ^{-3}]**	4.97 & -1.66*	19.65 & -16.84	10.19 & -9.45
**Flack**	N/A	0.500(14)	N/A

Compound	Ce_{24}^{PhMe}	Ce_{24}^{PhOMe}
**CCDC number**	2418699	2418700
**Formula**	$[Ce_{24}O_{28}(OH)_8(OOCPhMe)_{30}(Py)_4]_2(Py)_{42}$	$[Ce_{24}O_{28}(OH)_8(OOCC_6H_4(4\text{-}OMe))_{30}(py)_4](py)_{11}$
**X-ray source**	Mo Kα	Mo Kα
**wavelength [Å]**	0.71073	0.71073
**Empirical Formula**	$C_{365}H_{335}Ce_{24}N_{25}O_{96}$	$C_{315}H_{287}Ce_{24}N_{15}O_{126}$
**M [gmol^{-1}]**	9970.64	9661.65
**Crystal System**	triclinic	monoclinic
**Space Group**	P -1	$P\,2_1/c$
**Size [mm]**	0.05 × 0.1 × 0.1	0.098 × 0.164 × 0.220
**Colour / Shape**	yellow faceted block	Yellow block
**T [K]**	100.0(1)	100.0(1)
**a [Å]**	23.5031(2)	36.1757(6)
**b [Å]**	24.4019(2)	25.1668(4)
**c [Å]**	32.8406(3)	42.5226(8)
**α [deg]**	90.4670(7)	90
**β [deg]**	95.3987(8)	110.700(2)
**γ [deg]**	91.1491(7)	90
**V [Å^{-3}]**	18746.5(3)	36214.5(12)
**Z**	2	4
**θ range [deg]**	1.776 – 31.105	2.077 – 26.320
**Reflections**	409981	299949
**R int**	0.080	0.064
**data/restr/par**	98033/2394/4591	65029/2943/4321

**R1 [I>2σ(I)]**	0.0527	0.1024
**wR2 [all data]**	0.1363	0.4361
**GoF**	1.0114	1.7423
**Largest diff. peak and hole [eÅ^{-3}]**	3.05 & -2.31*	9.10 & -9.79*
**Flack**	N/A	N/A

**Compound**	Ce_{24}^{PhtBu}	Ce_{24}^{PhF}
**CCDC number**	2418814	2418679
**Formula**	$[Ce_{24}O_{28}(OH)_8(OOCPh^tBu)_{30}(Py)_4](py)_{10}$	$[Ce_{24}O_{28}(OH)_8(OOCC_6H_4(4\text{-}F))_{30}(py)_4]_2(py)_{12}$
**X-ray source**	Mo Kα	Cu Kα
**wavelength [Å]**	0.71073	1.54184
**Empirical Formula**	$C_{400}H_{460}Ce_{24}N_{14}O_{96}$	$C_{260}H_{170}Ce_{24}F_{30}N_{10}O_{96}$
**M [gmol^{-1}]**	10366.98	8903.07
**Crystal System**	monoclinic	triclinic
**Space Group**	P 2/c	P -1
**Size [mm]**	0.251 × 0.324 × 0.393	0.059 × 0.106 × 0.207
**Colour / Shape**	Brown block	Yellow block
**T [K]**	100.0(1)	100.0(1)
**a [Å]**	43.6537(3)	20.763300(14)
**b [Å]**	27.5054(2)	21.881899(14
**c [Å]**	40.9209(3)	38.498501(17)
**α [deg]**	90	84.2380(18)
**β [deg]**	103.9853(8)	82.4160(18)
**γ [deg]**	90	84.4420(18)
**V [Å^{-3}]**	47677.8(6)	17188.72(10)
**Z**	4	2
**θ range [deg]**	3.194 – 28.595	2.037 – 76.976
**Reflections**	522444	322782
**R int**	0.060	0.097
**data/restr/par**	63648/4926/4807	69426/782/3781
**R1 [I>2σ(I)]**	0.1140	0.090
**wR2 [all data]**	0.3748	0.3085
**GoF**	1.8700	1.0072
**Largest diff. peak and hole [eÅ^{-3}]**	10.28 & -2.83*	3.86 & -2.91*
**Flack**	N/A	N/A

**Compound**	Ce_{24}^{PhNH2}	Ce_{24}^{1Nap}	Ce_{24}^{2Nap}
**CCDC number**	N/A	N/A	2418677
**Formula**	$[Ce_{24}O_{28}(OH)_8(OOCPhNH_2)_{30}(Py)_4]_2(py)_x$	$[Ce_{24}O_{28}(OH)_8(OOCC_{10}H_7)_{30}(py)_4](py)_{3+x}$	$[Ce_{24}O_{28}(OH)_8(OOCC_{10}H_7)_{30}(py)_4](py)_{10}$
**X-ray source**	Mo Kα	Cu Kα	Mo Kα
**wavelength [Å]**	0.71073	1.5418	0.71073
**Empirical Formula**	$C_{400}H_{460}Ce_{24}N_{14}O_{96}$	$C_{365}H_{253}Ce_{24}N_7O_{96}$	$C_{400}H_{280}Ce_{24}N_{14}O_{96}$
**M [gmol^{-1}]**	10366.98	9635.89	10181.54
**Crystal System**	monoclinic	triclinic	orthorhombic
**Space Group**	P 2/m	P -1	Pbcn
**Size [mm]**	0.251 × 0.324 × 0.393	0.029 × 0.038 × 0.075	0.053 × 0.102 × 0.108
**Colour / Shape**	Brown block	Yellow block	Yellow block
**T [K]**	100.0(1)	100.0(1)	200.0(1)
**a [Å]**	23.8850(13)	25.3276(8)	26.0526(5)
**b [Å]**	41.3343(17)	29.1536(5)	42.5286(7)
**c [Å]**	24.0718(15	31.2965(5)	35.9018(6)
**α [deg]**	90	81.1151(15)	90
**β [deg]**	116.644(8)	82.178(2)	90
**γ [deg]**	90	82.311(2)	90
**V [Å^{-3}]**	47677.8(6)	22468.7(9)	39778.5(12)

**Z**	4	2	4
**θ range [deg]**	3.194 – 28.595	2.252 – 52.11	2.103 – 26.415
**Reflections**	522444	237510	158450
**R int**	0.060	0.181	0.075
**data/restr/par**	63648/4926/4807	20005 /0/1909	34778/1724/2406
**R1 [I>2σ(I)]**	0.1140	0. 1626	0.0777
**wR2 [all data]**	0.3748	0.4163	0.2566
**GoF**	1.8700	1.8911	0.9920
**Largest diff. peak and hole [eÅ^{-3}]**	105.10† & -26.34	6.87 & -5. 10	7.03 & -3.85*
**Flack**	N/A	N/A	N/A

† large residual electron density adjacent to Ce atoms.

**Compound**	**Ce_{24}^{Ph3F}**	**[Ce($O_2CCH_2{}^tBu$)$_3$(py)]$_\infty$**
**CCDC number**	2418676	2418701
**Formula**	[$Ce_{24}O_{28}(OH)_8(OOCC_6H_4(3\text{-}F))_{30}(py)_4](py)_{22}$	[$Ce(OOCCH_2{}^tBu)_3(py)]_4$
**X-ray source**	Mo Kα	Mo Kα
**wavelength [Å]**	0.71073	0.71073
**Empirical Formula**	$C_{340}H_{254}Ce_{24}F_{30}N_{26}O_{96}$	$C_{92}H_{152}Ce_4N_4O_{24}$
**M [gmol^{-1}]**	10172.70	2258.73
**Crystal System**	triclinic	hexagonal
**Space Group**	P -1	P 6_5 ‡
**Size [mm]**	0.106 × 0.119 × 0.282	0.042 × 0.107 × 0.200
**Colour / Shape**	yellow/brown prism	Yellow block
**T [K]**	100.0(1)	100.0(1)
**a [Å]**	20.8735(2)	14.11380(10)
**b [Å]**	21.7898(2)	14.11380(10)
**c [Å]**	23.7135(3)	93.9953(6)
**α [deg]**	63.3967(11)	90
**β [deg]**	73.8571(11)	90
**γ [deg]**	62.0940(12)	120
**V [Å^{-3}]**	8490.27(19)	16215.3(2)
**Z**	1	6
**θ range [deg]**	1.747 – 30.988	2.113 – 28.761
**Reflections**	225965	178301
**R int**	0.032	0.060
**data/restr/par**	44645/604/ 2463	25447/1/498
**R1 [I>2σ(I)]**	0.0387	0.0978
**wR2 [all data]**	0.1189	0.2642
**GoF**	1.0758	1.0644
**Largest diff. peak and hole [eÅ^{-3}]**	4.16 & -2.26	7.28 & -12.49
**Flack**	N/A	0.009(4)‡

‡ Most crystals collected were racemic twins one crystal (domain cleaved from crystal) with non-twinned structure is presented

References

[46] B. Zhao, R. J. Kashtiban, S. Huband, M. Walker, R. I. Walton, *Chem Asian J.* **2024**, *19*, e202401035.

[47] P. Makuła, M. Pacia, W. Macyk, *J. Phys. Chem. Lett.* **2018**, *9*, 6814-6817.

[48] T. Degen, M. Sadki, E. Bron, U. König, G. Nénert, *Powder Diffr.* **2014**, *29*, S13-S18.

[49] G. Winter, J. Beilsten-Edmands, N. Devenish, M. Gerstel, R. J. Gildea, D. McDonagh, E. Pascal, D. G. Waterman, B. H. Williams, G. Evans, *Protein Sci.* **2022**, *31*, 232-250.

[50] L. Palatinus, G. Chapuis, *J. Appl. Crystallogr.* **2007**, *40*, 786-790.

[51] P. W. Betteridge, J. R. Carruthers, R. I. Cooper, K. Prout, D. J. Watkin, *J. Appl. Crystallogr.* **2003**, *36*, 1487.

[52] G. M. Sheldrick, *Acta Crystallogr. Sect. A.* **2008**, *64*, 112-122.

[53] A. L. Spek, *Acta Crystallogr. Sect. C.* **2015**, *71*, 9-18.

[54] A. L. Spek, *Acta Crystallogr. Sect. D* **2009**, *65*, 148-155.

[55] aG. Christou, B. Russell-Webster, J. Lopez-Nieto, K. A. Abboud, *Angew. Chem. Int. Ed.* **2021**, 12591-12596; bK. J. Mitchell, K. A. Abboud, G. Christou, *Nat. Commun.* **2017**, *8*, 1445; cK. J. Mitchell, J. L. Goodsell, B. Russell-Webster, U. T. Twahir, A. Angerhofer, K. A. Abboud, G. Christou, *Inorg. Chem.* **2021**, *60*, 1641-1653.

[56] aW. J. Evans, T. J. Deming, J. M. Olofson, J. W. Ziller, *Inorg. Chem.* **1989**, *28*; bP. S. Gradeff, F. G. Schreiber, K. C. Brooks, R. E. Sievers, *Inorg. Chem.* **1985**, *24*, 1110-1111.

[57] B. Russell-Webster, J. Lopez-Nieto, K. A. Abboud, G. Christou, *Dalton Trans.* **2021**, *50*, 15524-15532.

[58] M. I. Naumova, E. A. Mainicheva, O. A. Gerasko, V. P. Fedin, *Russ. Chem. Bull.* **2009**, *58*, 1858-1865.

[59] V. Mereacre, A. M. Ako, M. N. Akhtar, A. Lindemann, C. E. Anson, A. K. Powell, *Helv. Chim. Acta* **2009**, *92*, 2507-2524.

[60] P. L. Roulhac, G. J. Palenik, *Inorg. Chem.* **2003**, *42*, 118-121.

[61] M. Schwanninger, J. C. Rodrigues, K. Fackler, *J. Near Infrared Spectrosc.* **2011**, *19*, 287-308.
